# Synthesis of homologous series of surfactants from renewable resources, structure–properties relationship, surface active performance, evaluation of their antimicrobial and anticancer potentialities

**DOI:** 10.1038/s41598-024-62905-3

**Published:** 2024-06-08

**Authors:** Shimaa A. Abdelaziz, Entesar M. Ahmed, M. Sadek

**Affiliations:** Chemistry Department, Faculty of Science, Al-Azhar University (Girls), Cairo, Egypt

**Keywords:** Surfactant, Cysteine, Antitumor activity, Glucosyl ester, Surface active performance, Hydrophile-lipophile balance (HLB), Carbohydrate chemistry, Glycoconjugates, Monosaccharides

## Abstract

Sugar esters display surface-active properties, wetting, emulsifying, and other physicochemical phenomena following their amphipathic nature and recognize distinct biological activity. The development of nutritional pharmaceuticals and other applications remains of great interest. Herein, three novel homologous series of several *N-*mono-fatty acyl amino acid glucosyl esters were synthesized, and their physicochemical properties and biological activities were evaluated. The design and preparation of these esters were chemically performed via the reaction of glucose with different fatty acyl amino acids as renewable starting materials, with the suggestion that they would acquire functional characteristics superior and competitive to certain conventional surfactants. The synthesized products are characterized using FTIR, ^1^H-NMR, and ^13^C-NMR spectroscopy. Further, their physicochemical properties, such as HLB, CMC, Γ_max_, γ_CMC_, and A_min,_ were determined. Additionally, their antimicrobial and anticancer efficiency were assessed. The results indicate that the esters' molecular structure, including the acyl chain length and the type of amino acid, significantly influences their properties. The measured HLB ranged from 8.84 to 12.27, suggesting their use as oil/water emulsifiers, wetting, and cleansing agents. All esters demonstrate promising surface-active characteristics, with moderate to high foam production with good stability. Notably, compounds 6-*O-*(*N*-dodecanoyl, tetradecanoyl cysteine)-glucopyranose (**34, 35**), respectively and 6-*O*-(*N-*12-hydroxy-9-octadecenoyl cysteine)-glucopyranose (**38**) display superior foamability. Wetting efficiency increased with decreasing the chain length of the acyl group. The storage results reveal that increasing the fatty acyl hydrophobe length enhances the derived emulsion's stability for up to 63 days. Particularly, including cysteine in these glucosyl esters improves wetting, foaming, and emulsifying potentialities. Furthermore, the esters exhibit antibacterial activity against several tested Gram-positive and Gram-negative bacteria and fungi. On the other hand, they show significant antiproliferative effects on some liver tumor cell lines. For instance, compounds 6*-O*-(*N-*12-hydroxy-9-octadecenoylglycine)-glucopyranose (**28**), 6-*O*-(*N*-dodecanoyl, hexadecanoyl, 9*-*octadecenoyl and 12-hydroxy-9-octadecenoylvaline)*-* glucopyranose (**29**, **31**, **32** and **33**), respectively in addition to the dodecanoyl, hexadecanoyl, 9-octadecenoyl and 12-hydroxy-9-octadecenoyl cysteine glucopyranose (**34**, **36**, **37** and **38**), respectively significantly inhibit the examined cancer cells.

## Introduction

Using sustainable starting substances as substitutes for fossil sources is now considered a fundamental principle of green chemistry^1–4^. In this regard, carbohydrates may be considered a suitable reserve for molecules based on carbon to prepare valuable industrial products. Carbohydrate esters produced from sugar and fatty acids are nonionic surfactants possessing unique, peculiar properties acting as functional ingredients in different applications, especially in food, cosmetic formulations^5–10^, agricultural and pharmaceutical fields^11–14^. For this reason, producing carbohydrate esters was a significant achievement of the sugar foundation^7,8,15,16^, they were approved first as food additives in Japan^17–19^ and as emulsifiers in different food products^20–24^. Their non-irritation and compatibility with skin suggested their valuable applications in cosmetics^18,25,26^, including hair treatments, eyelash products^27–29^, oil gels^28,30^ and deodorant formulations^25,28^.

Fatty acid esters of carbohydrates are crucial surfactants since they are recognized in broad fields, including biological and biochemical applications^15,31^, antibacterial, anti-inflammatory, and anticancer agents^32–34^. Additionally, they offered significant insights that contribute to a deeper comprehension of various biological processes, biochemistry, and therapeutics of glycoconjugate compounds. For instance, carbohydrate esters can be prepared by straightforward esterifying selected sugars with acylating compounds^35–37^, leading to products with different types of substitution.

Consequently, it is believed that upgrading certain low-priced sugars and sugar raw materials to bio-based derivatives could offer exceptionally acceptable new products in food and other industrial applications. The traditional sugar esters are typically simple structures, as the hydrophilic group consists of glucose or sucrose, and the fatty acyl chain is the hydrophobic moiety^38–40^. However, it is possible to prepare bio-based sugar ester surfactants from other functional materials, including polysaccharides, poly-alcohols, and amino acids. Such issues have attracted recent attention and motivated our imperatives to prepare and develop novel sugar ester-derived surfactants. In addition to non-toxicity, biodegradability, and renewable green substrates, their emulsifying, foaming, and wetting properties, these esters promote the interest in developing the preparation of novel products having opportunities for surfactants possessing a range of important surface activity and related performance. There is a growing emphasis on improving the surface active properties and further exploring the potential of sugar esters in such diversifying structures^39,41^. Additionally, the beneficial feasibility of synthesizing carbohydrate esters by simple chemical routes of reaction sugars with acylating compounds strongly motivated the development of sugar esters. Furthermore, novel green surfactants prepared from renewable green substrates have recently been essential targets due to increasing environmental protection consciousness and the pressing need for high-performance surfactants. Accordingly, this study involves modifying the simple sugar ester molecular structure firstly by incorporating specific other hydrophobic fatty acids (oleic and ricinoleic), secondly investigating the effect of introducing amino acids (glycine, valine, and specifically cysteine), as a linker between the hydrophobic acyl part and glucose moiety. The inclusion of oleic acid in these novel surfactants is the presence of an olefinic functionality in its molecular structure, which may confer surface-active effects, performance and biological potency such as wound healing^42^. Additionally, ricinoleic acid, a green vegetable substrate, possesses olefinic and hydroxyl groups, which could potentially influence the characteristics of these novel esters. For instance, it has been established that wetting characteristics are created when a hydrophilic group such as (OH) occurs at or near the center of a hydrophobic chain^43^. Moreover, cysteine, a significant component of the insulin hormone, and the integrity of the (–CH_2_SH) residue of this amino acid is crucial for its biological activity^44–46^ and probably other antimicrobial and antitumor effects^47^, consequently, it was introduced into the molecular structure of these newly sugar esters due to its unique properties.

Accordingly, because the different combinations of structure components may affect the physicochemical properties and performance-related potentialities, the ultimate goal of this study was to synthesize novel series of *N*-acyl amino acids-d-glucose esters using renewable glucose, fatty acids and amino acids substrates. Fourier transform infrared (FTIR) and nuclear magnetic resonance (^1^H-NMR & ^13^C NMR) spectroscopy prove the successful formation of these sugar esters. The physicochemical characteristics, including surface active properties, emulsification, foamability, and wetting power, were further examined for the possibility of their use in foods, cosmetics, and other industrial applications. The data of this study are correlated with a known commercial surfactant, sodium dodecyl sulfate (SDS). The antimicrobial and antiproliferative action against some tumor cell lines was also investigated.

## Materials and methods

### Materials

The starting materials and chemicals were of pure analytical grade; several fatty acids dodecanoic (lauric), tetra decanoic (myristic), hexadecanoic (palmitic), and 9-octadecenoic (oleic) were from Sigma-Aldrich (Germany). Glucose and amino acids (glycine, valine, and cysteine), castor oil from AL-Nasr Pharmaceutical Chemical Co., Egypt. Sodium hydroxide, acetone, ethyl alcohol, methanol, petroleum ether, SOCl_2,_ and chloroform were analytical grade chemicals. Silica gel, 200–300 mesh, was used for column chromatography. Melting points were determined using an electro-thermal LA 9000 SERIS Digital Melting Point Apparatus and are reported without correction. Ricinoleic acid was prepared from castor oil as follows:

### Preparation of ricinoleic acid^48^

Ricinoleic acid was prepared by refluxing 100 g of castor oil in a 200-ml ethanolic KOH solution under reflux for 1–2 h. After cooling, the ethanol was distilled, 250 ml of distilled water was added to the residue and extracted with 3 (20 ml) portions of diethyl ether in a separating funnel, and the organic layer was then separated. The aqueous solution was acidified with concentrated HCl to pH = 1, and the liberated free fatty acids were extracted by ethyl acetate, dried over anhydrous Na_2_SO_4_, filtered, and the extract obtained as an oily residue was mixed with *n*-hexane and cooled for 72 h. The organic solvent was removed by distillation under vacuum. Ricinoleic acid was obtained as a viscous yellow product b. p. = 246 °C, acid value = 179.5

### Methods of synthesis

Novel structure derivatives of fatty acyl amino acid glucose ester surfactants were synthesized by introducing an amino acid linker between the fatty acyl component and the glucose moiety. This was achieved via the esterification of glucose with *N*-fatty acyl amino acids obtained from the reaction of fatty acid chloride with selected amino acids. This strategy was believed to be effective in developing surface activity and related phenomena through structural modification of traditional sugar esters. Thus, for example, *O*-(*N*-fatty acyl amino)-d-glucose esters were prepared via the interaction of selected fatty acid chloride with the –NH_2_ function of the amino acid to produce *N*-fatty acyl amino acid, the –COOH of which is then bound with d-glucose. The route of the synthetic chemical procedures is described as follows, 6-*O-*(*N-*hexadecanoyl glycine)-glucopyranose was taken as an example for the preparation of the different glucose esters.

### Synthesis of the fatty acyl chloride^49^

Hexadecanoic acid (0.25 mol) was introduced in a dry 500-ml three-necked, round-bottomed flask fitted with a dropping funnel, a thermometer, and a water condenser. An exit tube is attached at the top of the condenser, allowing the HCl's evolution from the reaction to a gas-absorption trap. The flask is heated to 60 °C and thionyl chloride (0.5 mol) is added dropwise from the separating funnel with stirring using a magnetic stirrer. The reaction mixture was heated until no further evolution of HCl. The remaining excess thionyl chloride was distilled under vacuum, and the hexadecanoyl chloride was obtained and distilled off under reduced pressure, (yield 98%).

$${\text{RCOOH}} + {\text{SOCl}}_{2} \to {\text{RCOCl}} + {\text{SO}}_{2} + {\text{HCl}}$$

### Preparation of ***N***-fatty acyl amino acids (9-23 a)^50^

**Scheme 1:**
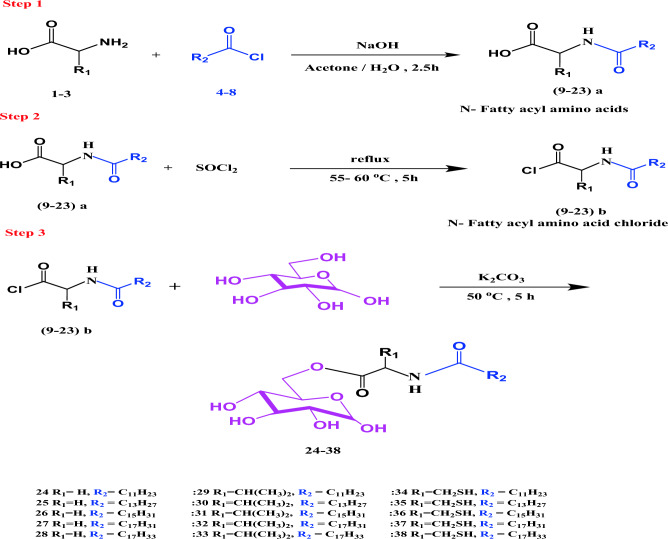
The general synthetic route of different glucosyl esters **24–38**.

*N*-fatty acyl amino acids are synthesized through the glycine, valine, or cysteine reactions, each with the selected fatty acyl chlorides, as presented in Scheme 1**.**

Compound **11** is an example of synthesizing the different fatty acyl amino acids.

#### Synthesis of N-hexadecanoyl glycine

Glycine **1** (4.5 g, 0.06 mol) is dissolved in a mixture of deionized water and acetone (1:2 v/v), 1 molar NaOH solution was added drop-wise into the mixture to reach a pH of 8.5–9.5. Hexadecanoyl chloride (10.48 g, 0.04 mol) was added drop-wise under stirring at 0–5 °C. The pH was kept at 7.5–11.5 using a NaOH (1 molar) aqueous solution. Then, the mixture was stirred at 0–5 °C for another 2.5 h and left overnight at room temperature. The solvent was distilled under vacuum, and then HCl solution (1 molar) was added until pH 1–2. The white solid ppt. was obtained by filtration, washed with deionized water until pH 7, and then with petroleum ether (20 ml) three times. The crude product was purified by crystallization from methanol to obtain **11a** (79.1%) m.p.: 120–122 °C. Compounds (**9–23a)** in **step 1** were obtained by the same method and yielded 71.5–81.5%. The free-COOH group of the obtained *N*-fatty acyl amino acids was activated by conversion into the corresponding acid chloride using thionyl chloride to obtain compounds **(9–23 b)** in **step 2**. The products were purified from petroleum ether and cold water, yielding 73.13–84.21%.

### Synthesis of glucosyl esters

6-*O-N*-acyl amino acids-d-glucosyl esters **24–38** were prepared as follows: glucose (1.0 mmol, as the acyl acceptor) and 1.5 mmol of *N*-fatty acyl amino acid chloride (as the acyl donor for the esterification reaction) were introduced into a 100 ml round-bottom flask together with 10 ml of *n*-hexane/THF (v/v, 1:1) under nitrogen flux, and 0.05 g anhydrous K_2_CO_3_ was then added. The reaction was carried out at 50 °C for 5 h. The reaction process was followed by silica gel thin layer chromatography with chloroform/methanol (v/v, 2:1).

After cooling, the reaction mixture was filtered, and the precipitate was washed three times with CHCl_3_. Finally, the product was purified by silica gel column chromatography using ethyl acetate: methanol (4:1).

The yield of each sample (**24–38)** was calculated according to the ratio of actual to theoretical production. All samples were characterized by FTIR, ^1^H-NMR, and ^13^C-NMR spectroscopy.

### Structural confirmation of the synthesized compounds

The chemical structures of the synthesized fatty acyl amino acid esters were assessed by IR spectra (KBr, υ cm) and were recorded on the CARY 630 FT-IR spectrometer (Agilent, Santa Clara, CA, USA). Additionally, NMR spectra were measured in (DMSO-d6) on a Bruker Avance (III) NMR spectrometer at 400 MHz; ^1^H & ^13^C-NMR at 400 and 100 MHz respectively (Bruker, Switzerland).

### Surface tension measurements and CMC values

Surface tension is an essential parameter of air–liquid interfacial properties. Thus, surface tension and related characteristics are determined by preparing aqueous solutions containing different sample concentrations. The surface tension is measured using the Theta optical tensiometer, Biolin Scientific Company, Finland, using the pendant drop technique. The surface tension is measured at 25 °C (298 K). Three values were determined for each sample, and the average was calculated to eliminate any possible contradictions.

The intersection of the measured isotherms in the surface tension versus concentration relationship graph is the surfactants' critical micelle concentration (CMC).

The surface tension at the critical micelle concentration used to calculate ***effectiveness*** (the surface pressure)^51^

1 $$\pi_{CMC \, = } \gamma_{\square } - \, \gamma_{CMC}$$

where: γ_□_: surface tension of pure water at 25 °C.

γ_CMC_: surface tension of the solution at the critical concentration of micelle formation.

The evaluation of the surface excess concentration (*Γ*_*max*_) is calculated from^52^ the Gibbs adsorption equation:

2 $$\Gamma_{\max } = \, - \left( {1/2.303nRT} \right) \, \left( {d\gamma /d\log \, C} \right)$$

where n defines the number of molecules at the interface between air and water, which changes as the concentration of the surfactant varies.

R = (8.314 J/(mol k^−1^).

T represents the absolute temperature (K).

(dγ/dlog C) denotes the variation of surface tension/variation of log concentration, which is the slope in the surface tension isotherm.

Area/molecule (A_min_)^53^ at the air /water interface can be determined via the following equation:

3 $$A_{\min } = \, 1 \, / \, (N_{A} \Gamma_{\max } )$$

where NA is Avogadro's number.

### The efficiency (PC_20_)^52^

The efficiency, represented by Pc_20_, refers to the concentration (in mol/L) of the surfactant solution that can reduce the surface tension by 20 mN/m and is obtained by the next equation:

$$Pc_{20} = \, - \log \, C_{20}$$

### The hydrophile–lipophile balance (HLB) values^54^

The HLB values of nonionic surfactants **24**–**38** can be calculated using the following formula:

$${\text{HLB }} = { 2}0\frac{{\text{Hydrophilic group molecular weight}}}{{\text{Total surfactant molecular weight}}}$$

### Foaming properties^22,55^

Foaming height (ml) was measured according to vigorous shaking of 25 ml of sample solution (1 wt.% and 0.1 wt.%) in a 100 ml glass stoppered graduated cylinder for 20 s.

### Wetting power

The Draves–Clarkson skein test is a frequently utilized method to evaluate the wetting capability of surfactants^56^. The procedure was adopted in this work at pH of slightly acidic (6.5) and slightly basic (7.5).

### Drop-penetration (D.P.T) method^57^

The drop penetration time was followed as an evaluation method and was applied to local commercial ground peatmoss, which was used to form a thickness of about 3–4 mm and added to a watch glass. A medicine dropper was used to apply two drops of a 0.1% solution of the tested surfactant ester to the peatmoss surface. The time taken for the penetration of liquid through peatmoss was recorded. After drying overnight, the samples were rewetted with distilled water.

### Emulsifying power^22^

The glucosyl ester solution of 3 ml (0.1 wt%) and sunflower oil (2 ml) were mixed in a 100-ml stopper cylinder by giving the cylinder 20 vigorous shakes. The test was employed at pH of 6.5 and 7.5. The emulsion volume was measured at 0, 5, 15, and 30-min intervals, and the time taken for any phase separation was recorded.

### Biodegradability^22^

The biodegradability was evaluated using the die-away in river water method by measuring the change in surface tension values versus biodegradation time.

### Biological activity

The antimicrobial activity of glucosyl esters against some selected pathogenic Gram-positive and Gram-negative bacteria and a fungus strain was evaluated using the nutrient agar well diffusion method^58^. The zone of inhibition in mm and the minimum inhibitory concentration (MIC) in μg/ml were measured for each sugar ester derivative. Different organisms were examined, including *Staphylococcus* *aureus* (ATCC 25923), *Bacillus* *cereus* as examples of Gram-positive bacteria, *Escherichia* *coli* (ATCC-25955), *Enterobacter* *cloacae* (ATCC-23355) as Gram-negative bacteria, in addition to *Saccharomyces* *cerevisiae* (ATCC-9763) and *Candida* *albicans* (ATCC-10231) as fungi. The minimum inhibitory concentration method (MIC) is defined as the lowest concentration of an antimicrobial that inhibits the visible growth of a microorganism after overnight incubation. The MIC of the synthesized GS was evaluated using the broth dilution method^59,60^.The procedure is described in detail in the Supplementary Materials.

### Cytotoxicity assay^61^

The HepG-2 cell line was used in this study to investigate the inhibition effects of the tittle esters on cell growth using the (3-[4,5-dimethylthiazol-2-yl]-2,5 diphenyl tetrazolium bromide) assay. Based on this colorimetric assay, the tetrazolium bromide (MTT) yellow derivative is changed into a formazan derivative with a purple color by mitochondrial succinate dehydrogenase in living cells. The method involves growing cell lines in an RPMI-1640 medium with 10% fetal bovine serum in an incubator at 37 °C with CO_2_. Penicillin 100 units/mL and 100 g/mL streptomycin were added. The cell lines were seeded at a density of 1.0 × 10^4^ cells per well in a 96-well plate at 37 °C for two days, followed by incubation. The cells were treated with varying amounts esters and re-incubated for one day. 20 μL of MTT solution containing 5 mg/mL was introduced and incubated for 4 h. The formation of the purple formazan in each well was dissolved by adding dimethyl sulfoxide (DMSO) (100 μL). The absorbance was recorded at 570 nm, and the viability was determined by multiplying the ratio of treated to untreated samples by 100.

## Results and discussion

Carbohydrate fatty acid esters, mainly sucrose, and glucose, are nonionic surfactants with significant and variable properties due to the presence of hydrophobic and hydrophilic moieties within the same molecule framework. Consequently, they play essential roles in the food, cosmetics, and pharmaceutical industries. Several conventional esters are synthesized by reaction under Fischer-type conditions using the relevant acid with the alcohols, but sugar esters cannot be obtained in such a direct way^62^. Accordingly, establishing relatively simple steps and inexpensive chemical synthesis of sugar fatty esters is particularly challenging given the multi-hydroxyl groups associated with carbohydrates^63^. However, synthesizing carbohydrate esters remains relatively simple because they are usually formed from glucose, sucrose, and fructose. Therefore, the conventional sugar esters' molecular framework was further developed by introducing other chemical substrates to obtain new molecular structures of sugar esters. Accordingly, in this work, three homologous series of glucosyl fatty esters were developed by incorporating an amino acid linker between the fatty acyl component and the glucose moiety of the fatty acyl glucose molecule. This was accomplished via a simple, straightforward method. Firstly, the fatty acyl chlorides **4–8** were reacted with the amino function of selected amino acids (glycine **1**, valine **2,** and cysteine **3**) to produce the corresponding *N*-acyl amino acids **9–23 a**. Finally, the carboxyl function of the latter products was bound to a hydroxyl group of d-glucose to afford 6-*O*-(*N*-fatty acyl amino)-d-glucose **24–38**, as illustrated in Scheme 1. The hydrophobic moiety consists of the long chain fatty acids (C_12_–C_16_, C_18:1_ and 12-hydroxy-9-C_18:1_) (**4**–**8**).

In theory, esterification with equimolecular quantities of the reactants could afford different possible sugar esters due to the presence of both primary and secondary alcoholic hydroxyl groups. However, these different types of hydroxyls react differently, and in practice, it is assumed that the primary OH groups react in preference to the secondary OH. Therefore, within the glucose molecule, the primary hydroxyl group in carbon 6 should be generally more reactive than 2^ry^ other-OH groups^64–66^. Consequently, esterification of the carboxyl component of the acyl amino acid took place in the OH group at C_6_ of glucose. IR, NMR, and ^13^C spectral results proved and confirmed this regioselective esterification. Furthermore, glucose is considered a suitable acyl acceptor for sugar ester synthesis, parallel to its better solubility than other sugars^67–69^. In order to make the leaving group move more quickly, fatty acyl chloride was introduced to replace fatty acid for the preparation of fatty acyl amino acid^70^.

### Identification and product analysis

**Figure 1:**
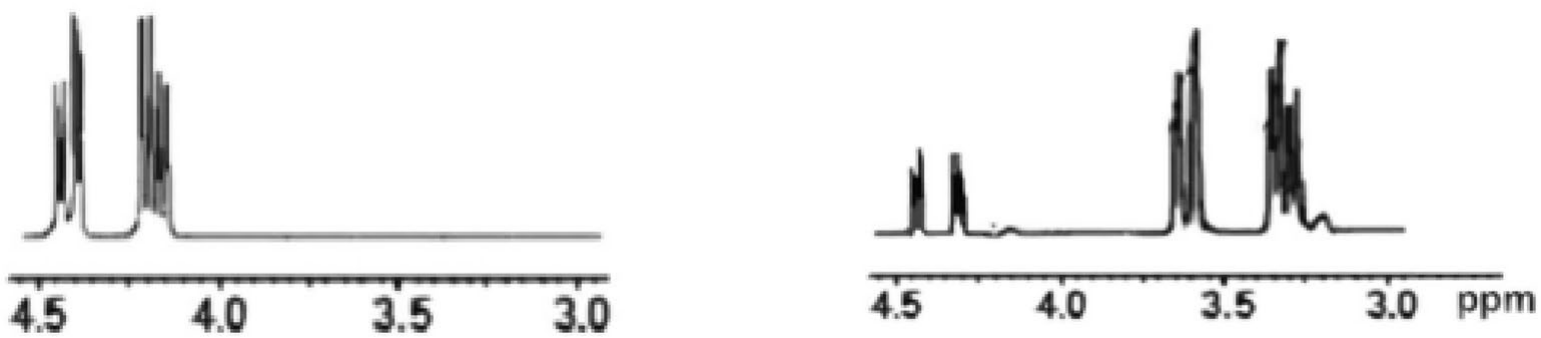
^1^H-NMR spectra of the synthesized glucose ester. (The signals at 4.11 and 4.45 ppm were obtained from the –CH_2_OCO– group.)

**Figure 2:**
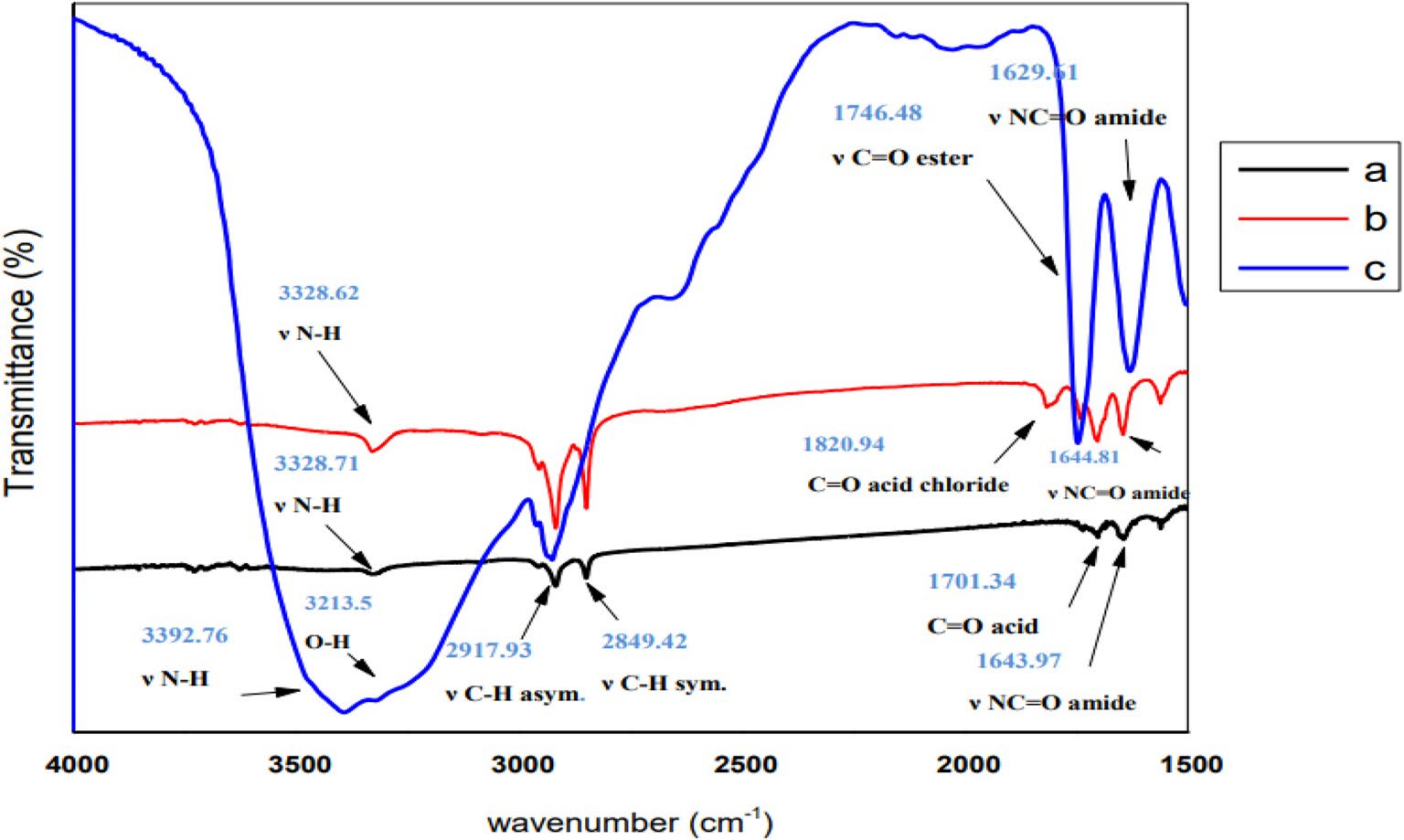
FTIR of (**a**) *N*-fatty acyl amino acid, (**b**) *N*-fatty acyl amino acid chloride, and (**c**) *N*-fatty acyl amino glucosyl ester.

The transformation of reactants into products that indicate the extent of the achievement of the reaction was confirmed via NMR monitoring. The ^1^H-NMR recorded additional multiple signals at 4.11 ppm and 4.45 ppm, as displayed in Fig. 1, which matched the –CH_2_OCO– of glucose ester, which indicated that the essential product may be identified as Glc-(CH_2_ ‘‘–6-O.COR)–. The signal intensity at 4.11 and 4.45 ppm derived from the –CH_2_OCO– group confirms the accomplishment of the esterification reaction at carbon 6. These data agree with the results reported by El-Baz et al*.*^71^, indicating that the esterification of the acylating agent usually occurs at the carbon 6 of the glucose molecule. Additionally, FTIR spectroscopy confirmed the chemical structures of the prepared 6*-O*-(*N-*fatty acyl)*-*glucopyranose, as shown in Fig. 2. FTIR of (**a**) *N*-fatty acyl amino acid shows a stretching vibration band at 3328.71 cm^−1^, indicating the presence of ν -N–H group. The sharp bands at 2917.93 cm^−1^ and 2849.42 cm^−1^ are due to the C–H. The firm peaks at 1643.97 cm^−1^ indicate the stretching vibration of the C=O group in the amide part, and the band at 1701.34 cm^−1^ is due to the C=O group of the carboxyl group of amino acid, which is shifted to the left at 1820.94 cm^−1^ due to the conversion to acid chloride in Figure (**b**), in the case of (**C**) this band was shifted to the right at 1746.48 cm^−1^ due to the formation of ester. The appearance of a broad band at 3213.5 cm^−1^ (**C**) is due to the O–H group of the sugar.

FTIR, ^1^H-NMR, and ^13^C-NMR spectroscopy confirmed the successful synthesis.

#### Chemical structure of *N*-hexadecanoyl glycine 11a

FT-IR (KBr, ν max cm^−1^) (Supplementary Fig. S1): 3328.71(ν NH stretch), 2917.93, 2849.42 (ν C–H asym. and sym. stretch), 1701.34 (ν C = O, acid) 1643.97 (ν C = O, amide).

**Figure 3:**
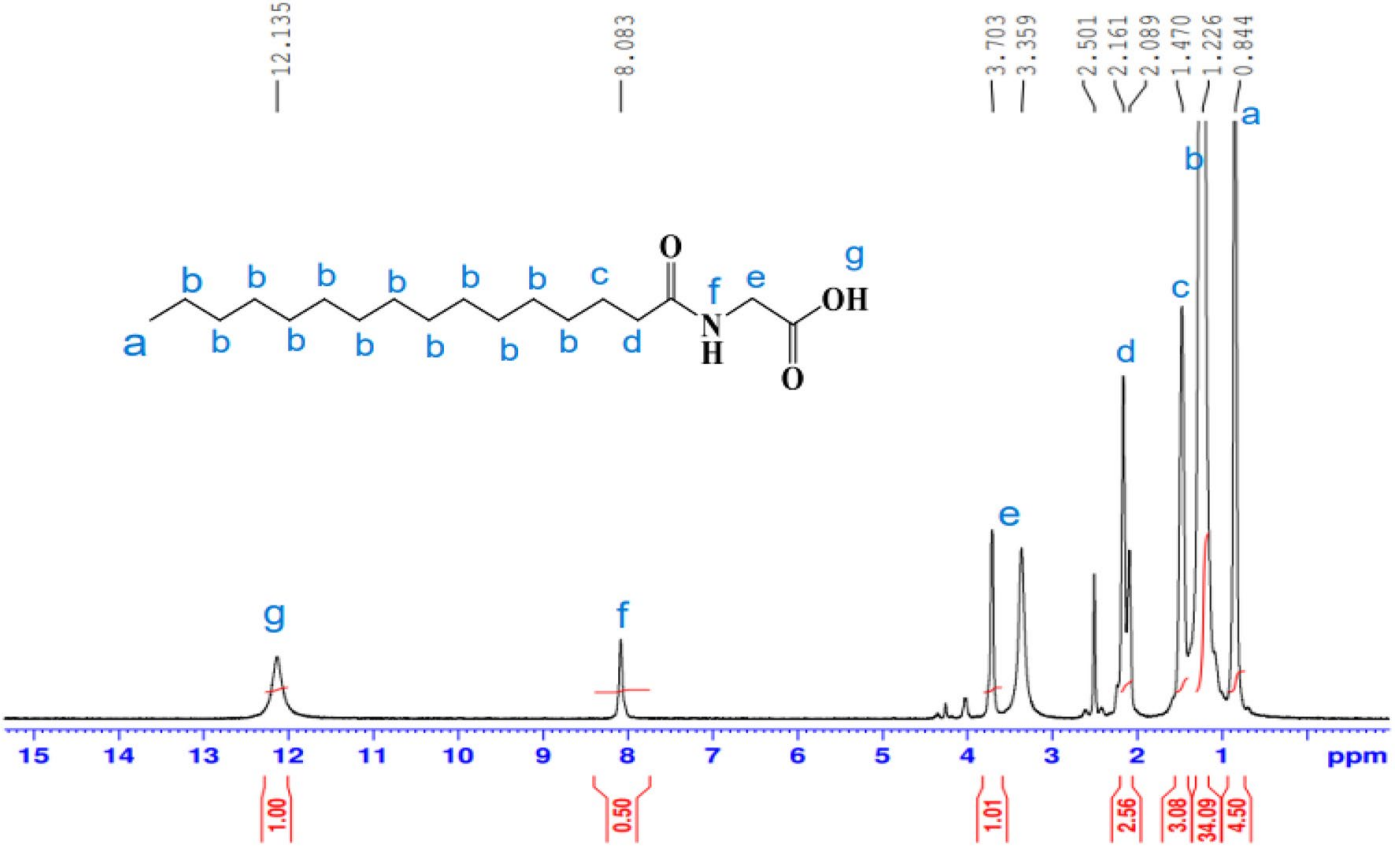
^1^H-NMR spectrum of *N-*hexadecanoyl glycine **11a.**

^1^H-NMR spectrum (400 MHz, DMSO, δ ppm): (Fig. 3) demonstrated various peaks at 0.84 (t, 3H, C**H**_3_); 1.22(s, 24H, C_12_**H**_24_); 1.47 (m, 2H, C**H**_2_CH_2_CO); 2.16 (t, 2H, CH_2_ C**H**_2_CO); 3.70 (d, 2H, NHC**H**_2_CO); 8.08 (t, 1H, N**H–**); 12.13 (s, 1H, –COO**H**). Other compounds were synthesized by the above method in the experimental section as white solids, and their structure confirmed was provided in supporting information.

**Figure 4:**
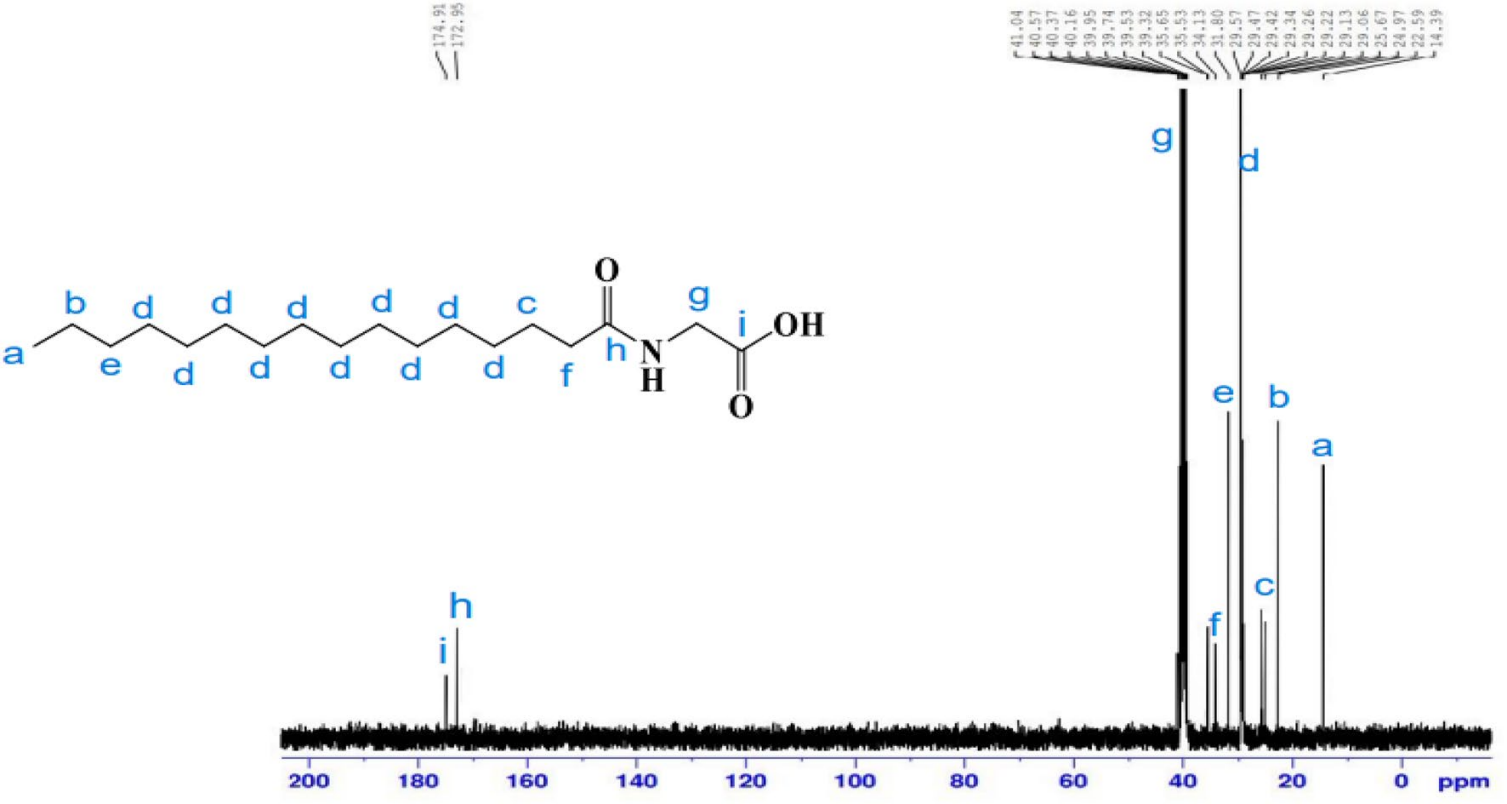
^13^C-NMR spectrum of *N-*hexadecanoyl glycine **11a.**

^13^C-NMR (100 MHz, DMSO, δ/ppm): spectrum (Fig. 4) illustrates peaks at 14.39 attributable to carbon of (**C**H_3_); 22.59(CH_3_**C**H_2_CH_2_); 24.97 (**C**H_2_CH_2_CO NH); 31.80, 29.57, 29.47, 29.42, 29.34, 29.26, 29.22, 29.13, 29.06, 25. 67 ((**C**H_2_)_10_ chain; 34.13 (CH_3_CH_2_**C**H_2_); 35.65 (CH_2_**C**H_2_CO NH); 41.04 (**C**H_2_-NH); 172.95 (NHC=O); 174.91(HO-**C**=O).

#### Chemical structure of *N*-hexadecanoyl glycine chloride 11b

FT-IR (KBr, ν max cm^−1^) (Supplementary Fig. S2): 3328.62 (ν NH stretch), 2917.54, 2849.16(ν C–H asym. and sym. stretch), 1820.94 (ν C=O, acid chloride) 1644.81 (ν C = O, amide).

**Figure 5:**
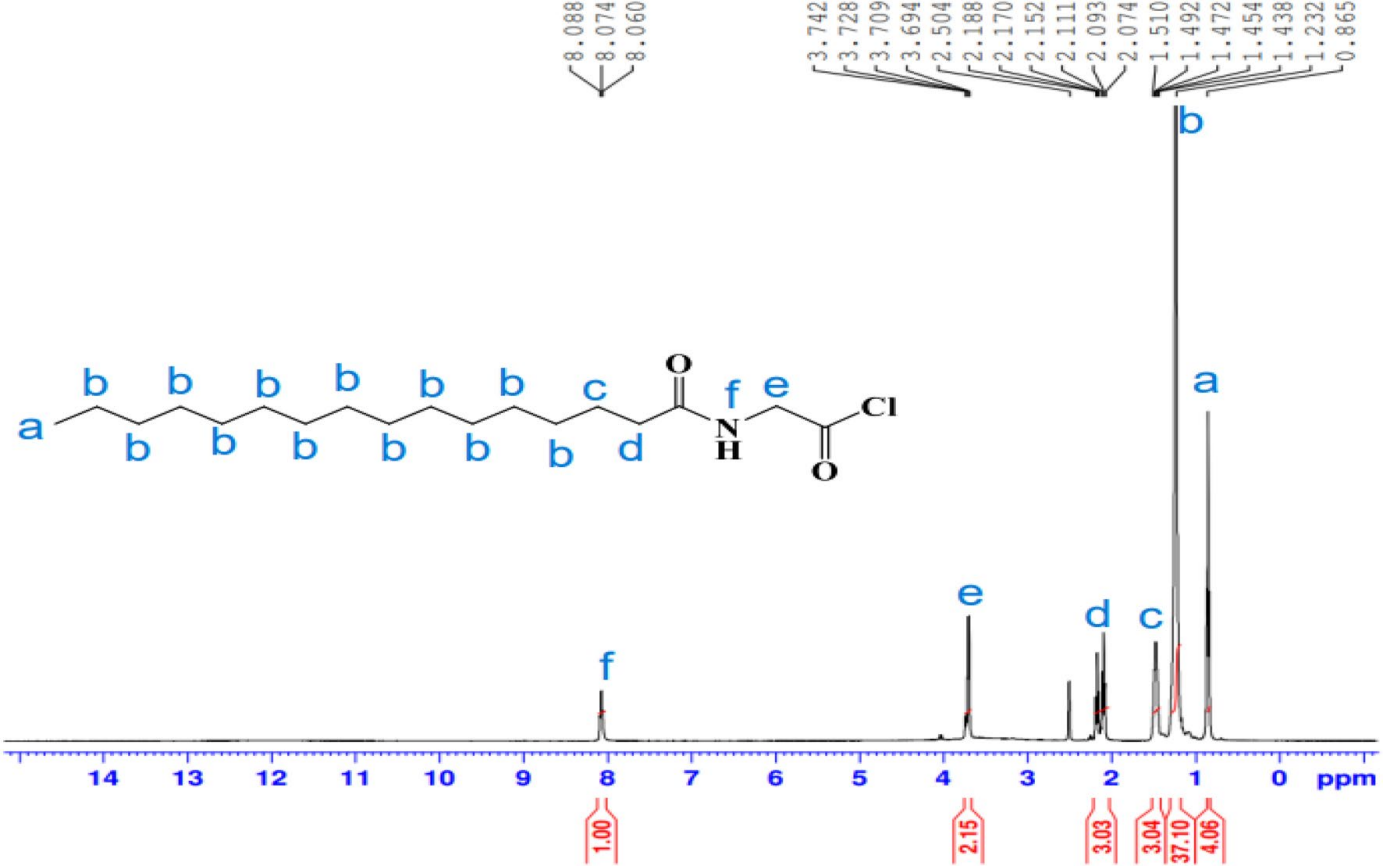
^1^H-NMR spectrum of *N-*hexadecanoyl glycine chloride **11 b.**

^1^H-NMR spectrum (400 MHz, DMSO, δ ppm): (Fig. 5) demonstrated various peaks at 0.86 (t, 3H, C**H**_3_); 1.23(m, 24H, C_12_**H**_24_); 1.51 (m, 2H, C**H**_2_CH_2_CO); 2.18 (t, 2H, CH_2_ C**H**_2_CO); 3.74 (d, 2H, NHC**H**_2_CO); 8.08 (t, 1H, N**H–**).

**Figure 6:**
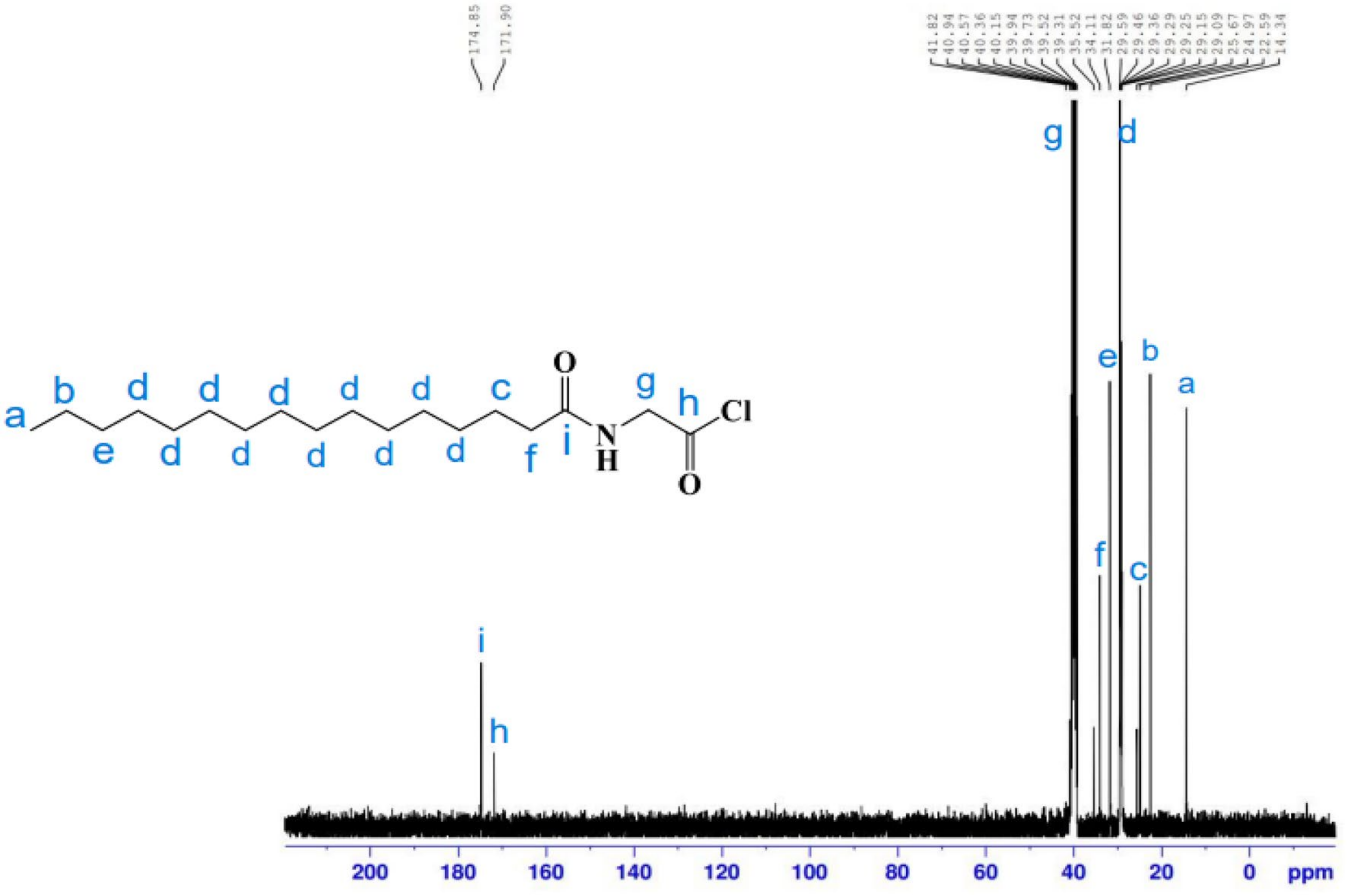
^13^C-NMR spectrum of *N-*hexadecanoyl glycine chloride **11 b.**

^13^C-NMR (100 MHz, DMSO, δ/ppm): spectrum (Fig. 6) illustrates peaks at 14.34 attributable to the carbon of (**C**H_3_); 22.59(CH_3_**C**H_2_CH_2_); 24.97(**C**H_2_CH_2_CO NH); 31.82, 29.59, 29.46, 29.36, 29.29, 29.25, 29.15, 29.09, 29.06, 25. 67 ((**C**H_2_)_10_ chain; 34.11 (CH_3_CH_2_**C**H_2_); 35.52 (CH_2_**C**H_2_CO NH); 41.82 (**C**H_2_-NH); 171.90 (Cl-**C**=O) 174.85(NHC=O).

#### Chemical structure of 6-*O*-(*N*-hexadecanoyl glycine)-glucopyranose (26)

**Figure 7:**
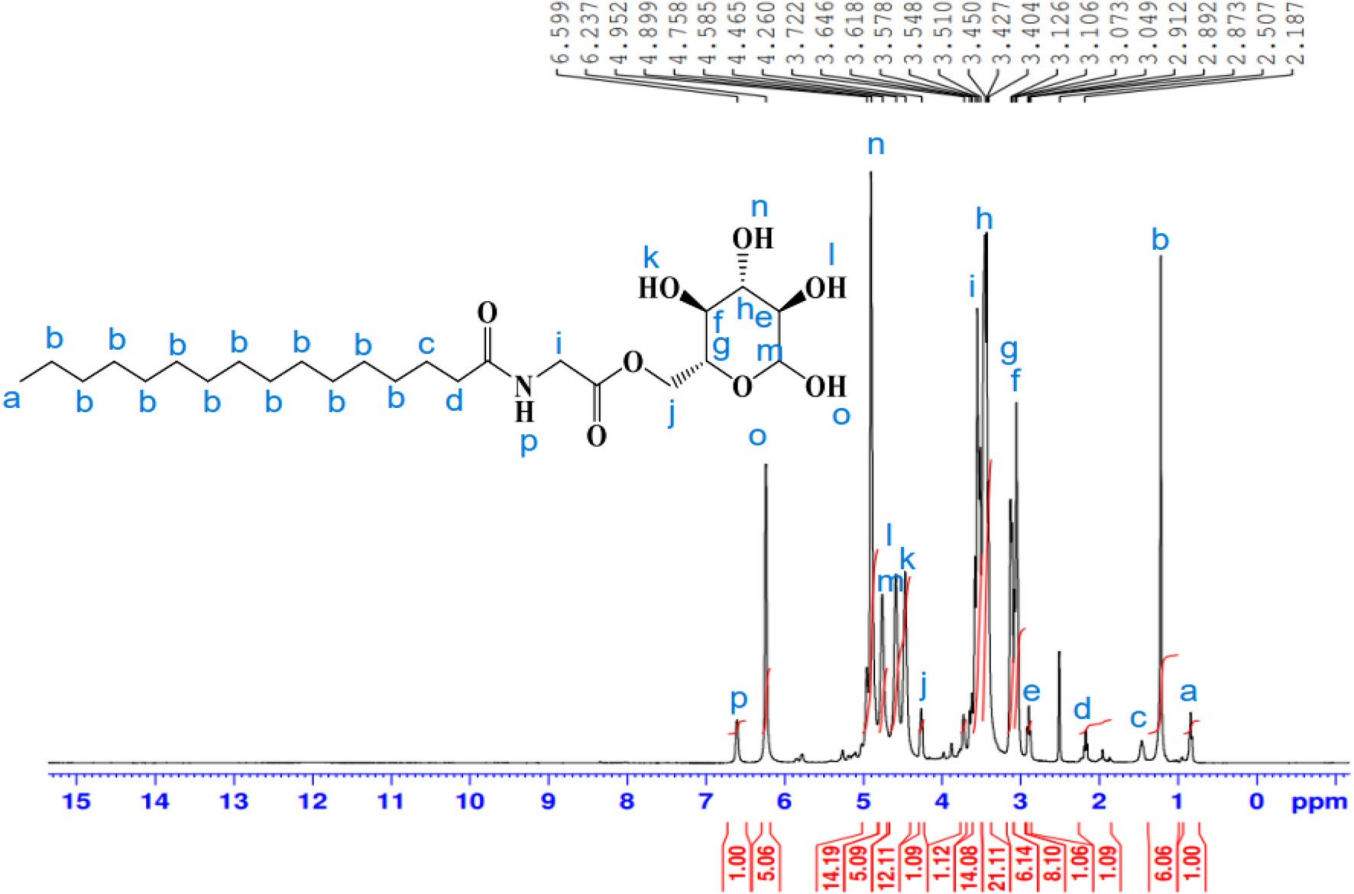
^1^H-NMR spectrum of 6*-O*-(*N-*hexadecanoyl glycine)-glucopyranose (**26**).

**Figure 8:**
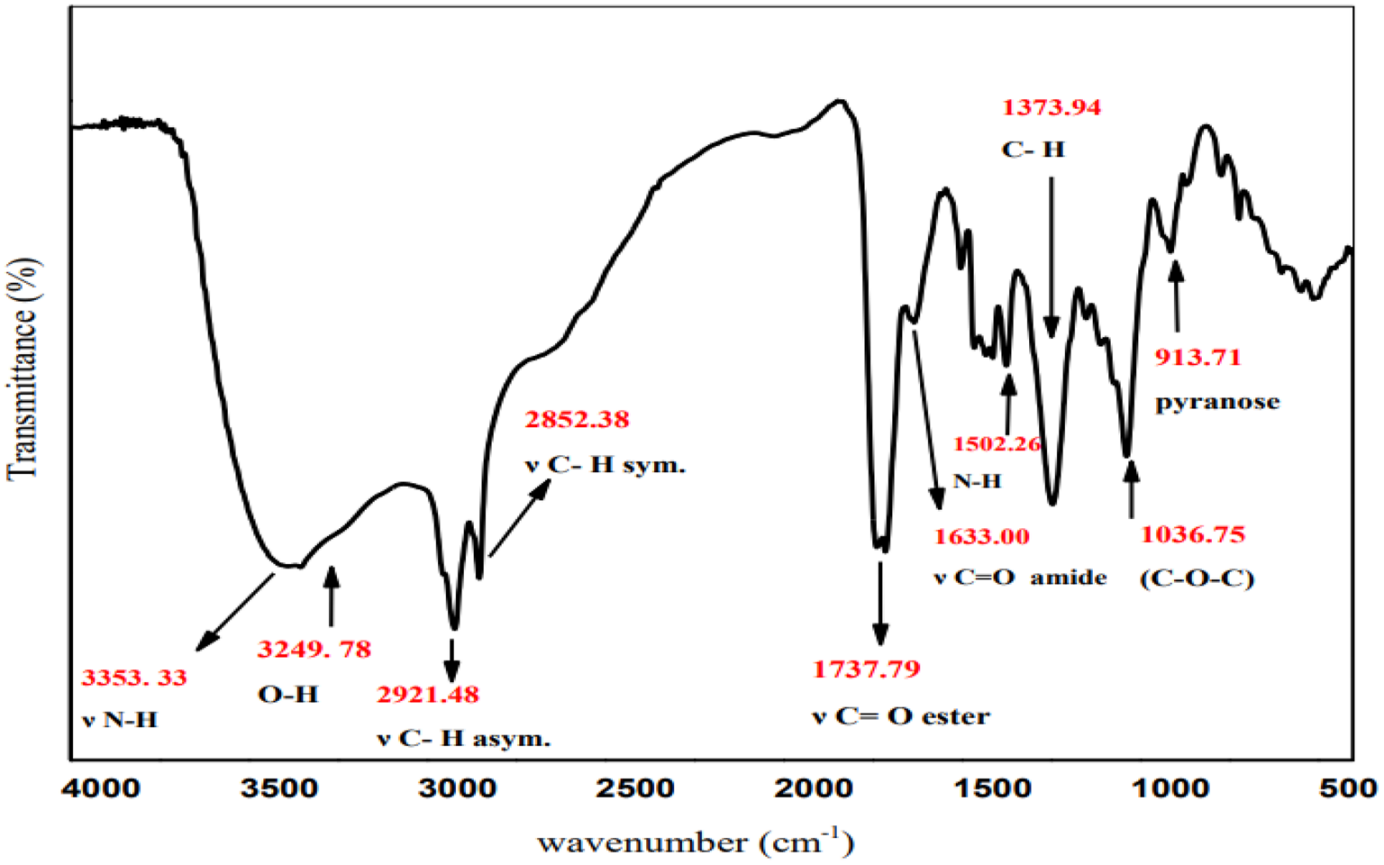
IR spectrum of 6*-O*-(*N-*hexadecanoyl glycine)-glucopyranose (**26**).

**Figure 9:**
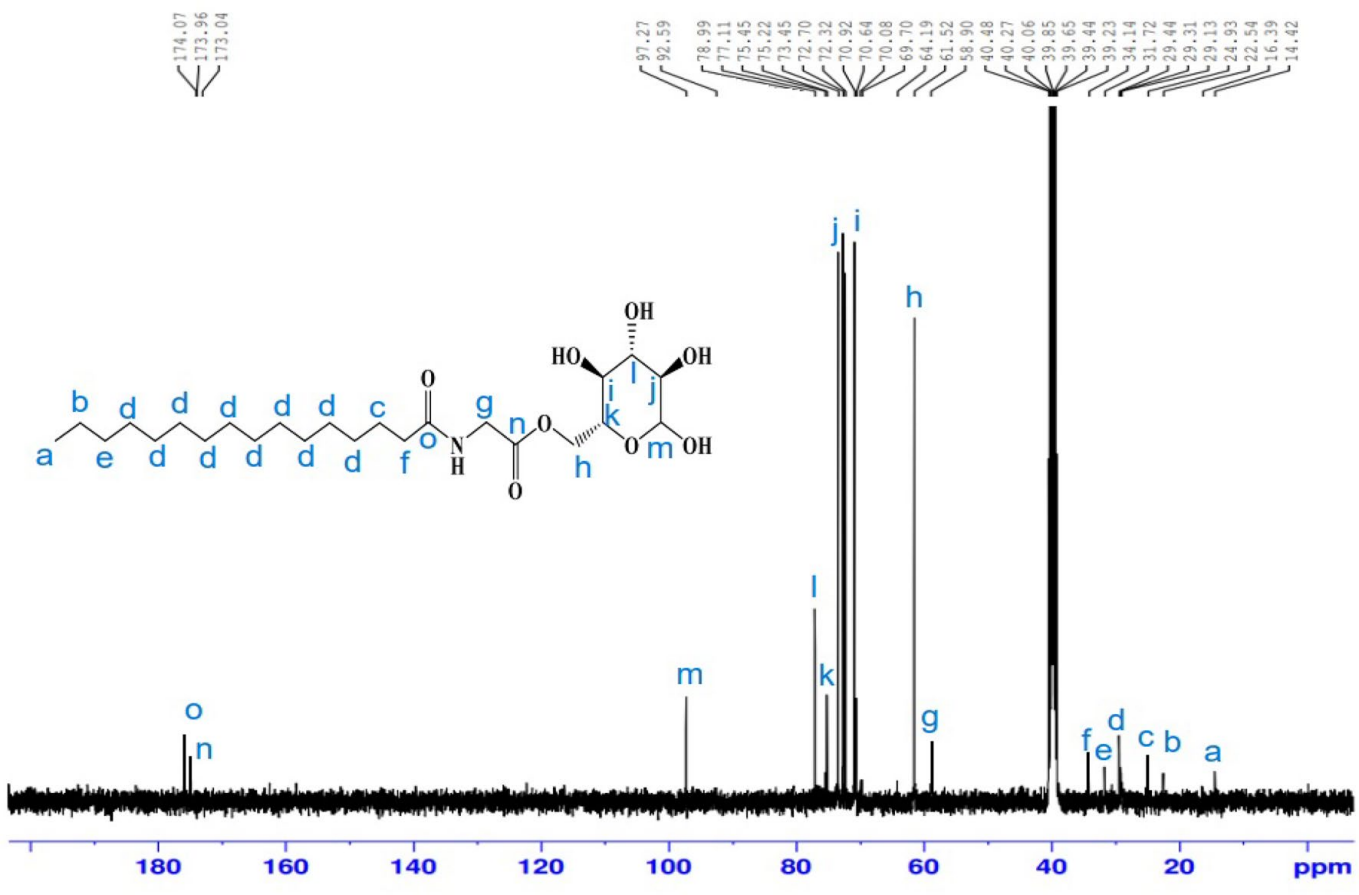
^13^C-NMR spectrum of 6*-O*-(*N-*hexadecanoyl glycine)-glucopyranose (**26**).

A white solid (86.2%); m.p.: 82–84 °C. ^1^H-NMR (400 MHz, DMSO-d6, δ/ppm) (Fig. 7): demonstrated various peaks at 0.85 (3H, C**H**_3_); 1.22(24H, (C**H**_2_)_12_); 1.47 (2H, C**H**_2_CH_2_CO); 2.18 (2H, C**H**_2_CO); 3.45(1H, **H**-2); 3.51 (1H, **H**-4); 3.61 (1H, **H**-5); 3.64 (1H, **H**-3); 3.72 (2H,NHC**H**_2_CO); 4.26 (2H, **H**-6); 4.46 (1H, O**H**-4); 4.58 (1H, O**H**-2); 4.75 (1H, **H**-1); 4.89 (1H, O**H**-3); 6.23 (1H, O**H**-1); 6.59 (1H, N**H–**). FT-IR (KBr, cm^−1^) (Fig. 8): 3353.33 (NH), 3249.78 (O–H), 2921. 48(C–H), 2852.38 (C–H), 1737.79 (C=O), 1633.00 (NC=O), 1502.26 (N–H), 1373.94 (C–H), 1036.75 (C–O–C), 913.71 pyranose ring. ^13^C-NMR (100 MHz, DMSO, δ/ppm) (Fig. 9): demonstrated various peaks at 14.42 attributable to carbon of (**C**H_3_); 22.54 (CH_3_**C**H_2_CH_2_); 24.93 (**C**H_2_CH_2_CO NH); 29.13, 29.31, 29.44 ((**C**H_2_)_10_ chain; 31.72 (CH_3_CH_2_**C**H_2_); 34.14 (CH_2_**C**H_2_CO NH); 58.90 (OCO **C**H_2_-NH); 64.19 (**C** 6); 72.32 (**C**-4); 72.70 (**C**-2); 75.22 (**C**-5); 77.11 (**C**-3); 97.27 (**C**-1); 173.04 (O–**C**=O); 173.30 (NHC=O).

#### Chemical structure of 6-*O*-(*N*-9-octadecenoyl glycine)-glucopyranose (27)

A white semi-solid (85.6%); mp.: 78–80 °C. ^1^H NMR (400 MHz, DMSO-d6, δ/ppm) (Supplementary Fig. S25): demonstrated various peaks at 0.83 (3H, C**H**_3_); 1.23 (20H, C**H**_2_ chain); 1.28 (2H, C**H**_2_CH_2_CO); 1.98 (4H, C**H**_2_CH=CHC**H**_2_); 2.17 (2H,–C**H**_2_CO); 3.45 (1H**, H**-2); 3.47 (1H, **H**-4); 3.52 (1H, **H**-5); 3.54 (1H, **H**-3); 3.59 (2H, C**H**_2_-NH–);4.40 (2H, **H**-6); 4.50 (1H, O**H**-4); 4.51 )1H, O**H**-2); 4.70(1H,**H**-1); 4.84 (1H, O**H**-3); 4.90 )2H, C**H**=C**H**); 5.32)1H, O**H**-1); 6.20 (1H, N**H**–).FT-IR (KBr, cm^−1^) (Supplementary Fig. S7): 3392.18 (N–H), 3252.79 (O–H), 2926.83 (C–H), 2855.18 (C–H), 1711.72 (C=O), 1629.30 (NC=O), 1461.22 (N–H), 1385.43 (C–H), 1052.58 (C–O–C), 924.80 pyranose ring.

#### Chemical structure of 6-*O*-(*N*-tetradecanoyl valine)-glucopyranose (30)

A white solid (79.7%); ^1^H NMR (400 MHz, DMSO-d6, δ/ppm) (Supplementary Fig. S20): demonstrated various peaks at 0.84 (3H, C**H**_3_); 0.97 (6H, (C**H**_3_)_2_); 1.23 (20H, C**H**_2_ chain); 1.47 (2H,–C**H**_2_CH_2_CO); 2.19 (1H, C**H** (CH3)_2_); 2.89(2H,–C**H**_2_CO); 3.11 (1H, **H**-2); 3.53 (1H, **H**-4); 3.55 (1H, **H**-5); 3.64 (1H, **H**-3); 4.26 (1H, COC**H**-NH); 4.40 (2H, **H**-6); 4.45 (1H, O**H**-4); 4.69 (1H, O**H**-2); 4.84 (1H, **H**-1); 4.89 (1H, O**H**-3); 4.90 (1H, O**H**-1); 8.20 (1H,N**H**–). FT-IR (KBr, cm^−1^) (Supplementary Fig. S10): 3368.04 (N–H), 3245.54 (O–H), 2937.92 (C–H), 2884.80 (C–H), 1730.05 (C=O), 1663.89 (NC=O), 1607.65 (N–H), 1364.22 (C-H), 1050.52 (C–O–C), 925.67 pyranose ring.

#### Chemical structure of 6-*O*-(*N*-12-hydroxy-9-octadecenoylvaline)-glucopyranose (33)

A white—semi solid (82.1%); ^1^H NMR (400 MHz, DMSO-d6, δ/ppm) (Supplementary Fig. S21): demonstrated various peaks at 0.84 (3H, C**H**_3_); 0.96 (6H*,* 2xCH_3_); 1.22(16H, C**H**_2_ chain); 1.47 (2H, CH=CHCH_2_CHOH C**H**_2_);1.87 (2H,–C**H**_2_CH_2_CO), 2.00 (2H,C**H**_2_CH=CH); 2.07(1H,–C**H**(CH3)_2_); 2.22 (2H,–C**H**_2_CO); 2.33 (2H, CH=CHC**H**_2_CHOH); 3.47 (1H,** H**-2); 3.57 (1H,** H**-4); 3.63 (1H, **H**-5); 3.64(1H, **H-**3); 3.65 (1H, CH_2_C**H**OH); 3.90 (2H, **H**-6); 4.26 (1H,CONHC**H**–); 4.40 (1H, O**H**-4); 4.55 (1H, O**H**-2); 4.69 (1H, O**H**-3); 4.84 (1H, **H-**1); 4.87 (2H, C**H**=C**H**); 4.90 (1H, O**H**-1); 4.94 (1H, –CHO**H**); 6.21 (1H, –N**H–**). FT-IR (KBr, cm^−1^) (Supplementary Fig. S13): 3368.28 (N–H), 3246.70 (O–H), 2935.18 (C–H), 2863.70 (C–H), 1737.46 (C=O), 1663.94 (NC=O), 1550.31 (N–H), 1365.02 (C–H), 1077.87 (C–O–C), 925.91 pyranose ring.

#### Chemical structure of 6-*O*-(*N*-dodecanoyl cysteine)-glucopyranose (34)

A white solid (90.1%); ^1^H NMR (400 MHz, DMSO-d6, δ/ppm) (Supplementary Fig. S22): demonstrated various peaks at 0.83 (3H, C**H**_3_); 1.23 (16H, C**H**_2_ chain); 1.47 (2H, C**H**_2_ CH_2_CO); 1.84 (1H, S**H**); 2.19 (2H, –C**H**_2_CO); 2.91 (2H, CHC**H**_2_SH); 3.52 (1H, **H-**2); 3.54 (1H, **H-**4); 3.63 (1H, **H-**5); 3.74 (1H, **H-**3); 4.26 (2H*,* **H-**6); 4.27 (1H, O**H-**4); 4.68 (1H, NHC**H**CH_2_); 4.69 (1H, O**H-**2); 4.66 (1H, **H-**1); 4.74 (1H, O**H-**3); 4.91 (1H, O**H-**1); 7.50 (1H, N**H–**). FT-IR (KBr, cm^−1^) (Supplementary Fig. S14): 3391.17 (N–H), 3297.03 (O–H), 2920.26 (C–H), 2851.26 (C–H), 2673.99 (S–H), 1729.50 (C=O), 1622.76 (NC=O), 1584.14 (N–H), 1383.88 (C–H), 1056.44 (C–O–C), 919.88 pyranose ring. ^13^C-NMR (100 MHz, DMSO, δ/ppm) (Supplementary Fig. S26): demonstrated various peaks at 14.43 attributable to carbon of (**C**H_3_); 22.54 (CH_3_**C**H_2_CH_2_); 24.94 (**C**H_2_CH_2_CO NH); 29.13 (HS**C**H_2_CH), 29.32, 29.45 ((**C**H_2_)_10_ chain; 31.73 (CH_3_CH_2_**C**H_2_); 34.15 (CH_2_**C**H_2_CO NH); 58.92 (OCO **C**H-NH); 64.21 (**C** 6); 70.91 (**C**-4); 72.32 (**C**-2); 75.46 (**C**-5); 78.95 (**C**-3); 97.28 (**C**-1); 174.99 (O–**C**=O); 175.98 (NHC=O).

### Chemical structure of 6-*O*-(*N*-hexadecanoyl cysteine)-glucopyranose (36)

A white solid (85.1%); ^1^H NMR (400 MHz, DMSO-d6, δ/ppm) (Supplementary Fig. S23): demonstrated various peaks at 0.84 (3H, CH_3_); 1.22 (24H, C**H**_2_ chain); 1.46 (2H, C**H**_2_ CH_2_CO–);1.96(1H, S**H**); 2.17 (2H,C**H**_2_CO–);3.03 (2H, CHC**H**_2_SH); 3.45; (1H*,* **H-**2); 3.51 (1H*,* **H-**4); 3.54 (1H*,* **H-**5); 3.55 (1H, **H-**3); 3.58 (2H, **H-**6); 4.04 (1H*,* O**H-**4); 4.41 (1H, NHC**H**CH_2_); 4.52 (1H, O**H-**2); 4.71 (1H, **H-**1); 4.85 (1H, **HO-**3); 4.91 (1H, O**H-**1); 6.21(1H, N**H)**. FT-IR (KBr, cm^−1^) (Supplementary Fig. S16): 3368.20 (N–H), 3248.13 (O–H), 2921.10, 2850.55 (C–H), 2657.96 (S–H), 1738.62 (C=O), 1664.15 (NC=O), 1461.93 (N–H), 1384.43 (C–H), 1050.11 (C–O–C), 925.76 pyranose ring.

#### Chemical structure of 6-*O*-(*N*-12-hydroxy-9-octadecenoyl cysteine)-glucopyranose (38)

Yellowish white—semi (79.7%); ^1^H NMR (400 MHz, DMSO-d6, δ/ppm) (Supplementary Fig. S24): demonstrated various peaks at 0.82 (3H, C**H**_3_); 1.23 (16H, C**H**_2_chain); 1.47 (2H, OHCH C**H**_2_); 1.53 (2H, C**H**_2_CH_2_CO); 1.96 (1H, S**H**); 2.17(2H, C**H**_2_CH=CH); 2.23 (2H,–C**H**_2_CO); 2.25(2H, CH=CHC**H**_2_CHOH); 3.05 (2H, C**H**_2_SH); 3.37 (1H, **H-**2); 3.40 (1H, **H-**4); 3.43 (1H, **H-**5); 3.52 (1H, **H-**3); 3.54 (1H, CH_2_C**H**OH); 3.58 (2H, **H-**6); 4.03 (1H, O**H**-4); 4.41 (1H, NHC**H**CH_2_); 4.50 (1H,O**H-**2); 4.74 (1H, **H-**1); 4.84 (1H, O**H-**3); 4.90(2H,C**H**=C**H**); 5.31 (1H, O**H-**1); 5.43 (1H,–CHO**H**); 6.20 (1H.N**H–**). FT-IR (KBr, cm^−1^) (Supplementary Fig. S18): 3432.02 (N–H), 3245.39 (O–H), 2932.33, 2858.45 (C–H), 2555.71 (S–H), 1737.98 (C=O), 1663.37 (NC=O), 1461.11 (N–H), 1365.09 (C–H), 1077.91 (C–O–C), 925.91 pyranose ring. The spectral data figures of the other prepared (*N*-fatty acyl amino)-glucosyl esters are also provided in ESI.

As expected, the hydrophobic character increases parallel to the acyl chain length within the same amino acid-bearing ester. The results emphasize the feasibility of the simple chemical methods to accomplish these novel structures of sugar ester derivatives.

### Surface active properties

As expected, the performance of the glucose acyl amino esters having 12 or more hydrophobic acyl chain lengths displays surface active function due to the amphiphilic nature of these homologs' series of surfactants.

### Surface tension

Surfactant characteristics such as CMC and other surface parameters are determined in order to evaluate the surface action efficiency of these synthesized molecular structures. The surface tension of aqueous solutions of the esters **24–38** was examined versus different concentrations of the esters, enabling exploration of the behavior of these surfactants towards the formation of emulsions, foamability, and other essential properties for food and other industrial applications. The CMC, γ_CMC_, Γ_max_, A_min_, and *PC*_20_ values of glucosyl esters **24–38** at 25 °C are listed in Table 1. These results were compared to a commercial surfactant, sodium dodecyl sulfate (SDS). The potential for surface tension reduction in all candidates increases significantly compared to pure distilled water.

**Table 1:** Surface active parameters of compounds **24–38** aqueous solution at 25 °C.

Surfactant	CMC (mmol/L)	ϒCMC (m N/m)	π_CMC_ (m N/m)	Pc_20_	Γ_max_ (μmol/m ^2^)	A_min_ (nm^2^)
SDS	4.67	33	39	5.1	2.02	0.82
24	3.16	38	34	4.5	1.83	0.91
25	2.34	37.9	34.1	4.6	1.65	1.01
26	1.66	37.2	34.8	4.8	1.59	1.05
27	1.82	36.9	35.1	4.9	1.28	1.29
28	1.79	36.5	35.5	4.9	1.25	1.33
29	2.39	37.1	34.9	4.55	1.51	1.10
30	1.94	36.5	35.5	4.8	1.42	1.17
31	1.74	35.5	36.5	4.82	1.33	1.25
32	1.66	35.3	36.7	4.91	1.24	1.34
33	1.62	35.1	36.9	4.95	1.22	1.36
34	2.82	37.5	34.5	4.6	1.67	0.997
35	2.46	37.1	34.9	4.7	1.51	1.1
36	1.86	36.8	35.2	4.75	1.40	1.18
37	1.78	36.3	35.7	4.8	1.28	1.30
38	1.74	36	36	4.9	1.24	1.34

*S.D.S* Sodium dodecyl sulfate.

**Figure 10:**
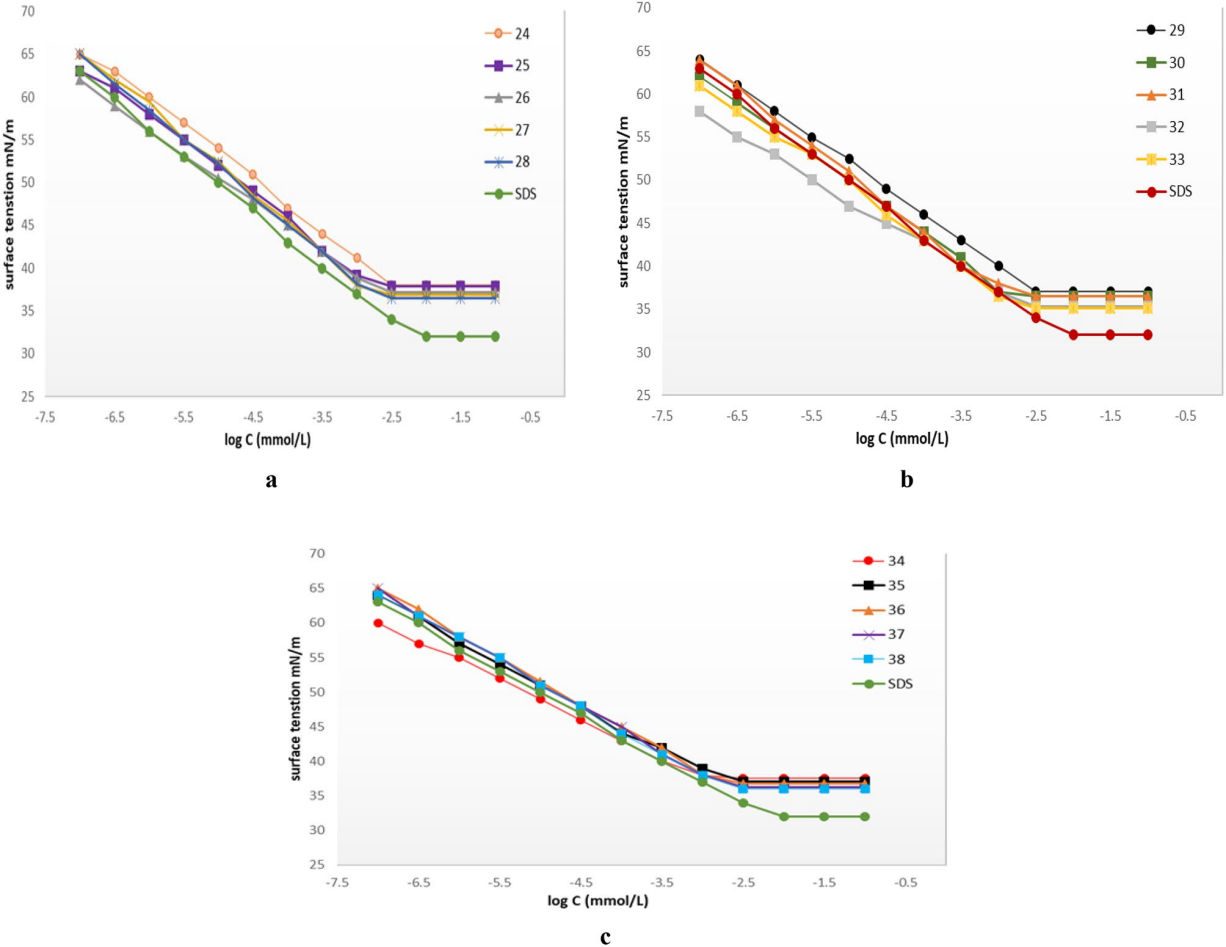
(**a**–**c**) Surface tension (**ϒ)** Vs log (**c**) of prepared glucosyl esters **24–38***.*

The determined critical micelle concentrations (CMC) are ensured by relating the surface tension versus concentration isotherms, where their values decrease with increasing the chain length of the hydrophobic acyl chain, as illustrated in Fig. 10a–c. At this specific concentration (CMC), the surfactants aggregate to build up micelle structures. These results are of great practical value since they indicate the surfactant needed to solubilize hydrophobic materials^18^. Generally, CMC values are related to the hydrophobic chain length of the surfactant in such a way that the greater the chain length, the smaller the values of CMC. The glycine-bearing homologs series attained CMC values of 3.16, 2.34, and 1.66 mmol/l for dodecanoyl, tetradecanoyl, and hexadecanoyl fatty acyl side chains, respectively. Those recorded for valine-and cysteine-based candidates were 2.39, 1.94, 1.74 and 2.82, 2.46, and 1.86 mmol/l, respectively. This range was effective as compared to SDS (4.67 mmol/l)^51^. The octadecenoyl and hydroxy-octadecenoyl derivatives have slightly lower CMC values (1.62, 1.82 mmol/l), irrespective of the type of amino acids in these homologs' series. The minimum surface tension at CMC (γ_CMC_) of these candidates is not significantly affected by variations in the associated side chain length of the glucosyl esters. All members of the synthesized esters display satisfactory surfactant characteristics, which may make them very attractive nonionic products. In contrast to the notable trends mentioned before, the unsaturation of the hydrophobic fatty acyl substituent has a negligible effect on the surface activity of the esters, as demonstrated by the octadecenoyl and hydroxy-octadecenoyl congeners. The results suggest that a single unsaturation in acyl chain length greater than C_12_ does not considerably affect the principal properties of these surfactants.

The PC_20_ values increased while CMC and **γ**_CMC_ decreased as the fatty acyl chain length was longer within the same amino acid homologous series. The adsorption of these esters in solution showed positive values parallel to Γ_max_ values. The calculated values were 1.83, 1.65, 1.59, 1.28, 1.25 and 1.51, 1.42, 1.33, 1.24, 1.22, and 1.67, 1.51, 1.40, 1.28, and 1.24 μmol/m^2^ for compounds **24–38**, respectively, which explains that the molecular concentration at the surface layer is higher than that in the bulk solution^53^. As predicted, it is reasonable that the minimum cross-sectional area (A_min_) is affected by the molecular framework of the surfactants, particularly the type of amino acid and the fatty acyl chains. It may be observed that A_min_ values increase with the length of the hydrophobic chain.

### Foaming properties

**Figure 11:**
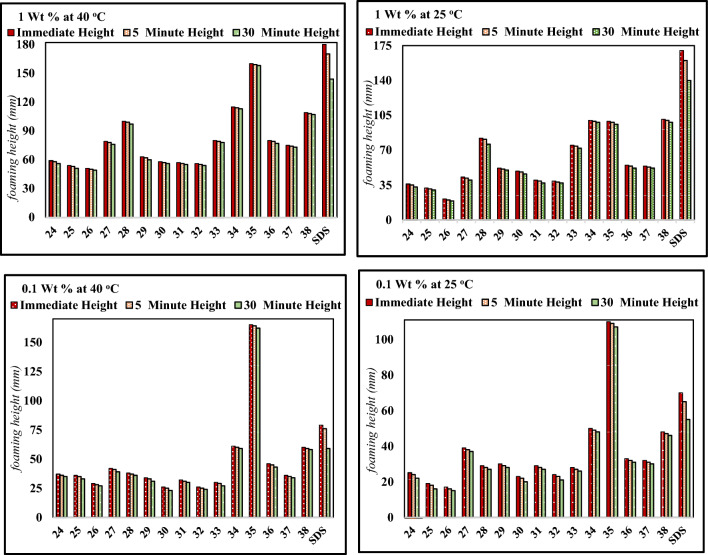
Foam height of *N*-fatty acyl amino glucopyranosyl esters at concentrations of 1 and 0.1 wt % at 25 °C and 40 °C.

Foaming is another essential function of aqueous surfactant solutions. Food products with aerated textures are everywhere, such as bread and cakes. This aerated food material acquires particular features, making the food products readily acceptable for widespread consumption^72,73^. Incorporating gas bubbles as a whole or partial replacement for dispersed fat droplets makes creating and stabilizing foams an essential aspect of commercial food processing. Introducing air bubbles as replacements for dispersed fat droplets in food processing generates and stabilizes valuable foam in some commercial food manufacturing industries. Surfactants and low-molecular-weight proteins are helpful materials for foam generation. Accordingly, the foam produced by the newly synthesized esters is determined at 0.1% and 1.0% (w/w) at 25 °C and 40 °C (Fig. 11). The different esters in this work, display moderate to high foam heights that persist for varying periods compared to a commercial surfactant, sodium dodecyl sulfate (SDS), a trend similar to other nonionic surfactants^74,75^. A positive correlation exists between foamability and ester concentration. However, the latter (0.1% and 1.0%) are above the CMCs (Table 1) for these esters, yet foam production varies with increasing concentration, which indicates that the CMC and the molecular weight of these esters determine their adsorption rates. The foam volume generated throughout shearing or agitation of the aqueous solutions of the esters is a measure of foamability. This may be attributed to the ability of surfactant molecules to reduce the surface tension of the solvent in which they are dissolved, thus facilitating aeration and foam formation.

Generally, the foamability of the ester solution decreases as the acyl side-chain length becomes more extended, resulting in higher molecular weight and greater lipophilicity, reducing the adsorption of molecules and their migration rates at the air/water interface^76^. These results are consistent with the prediction that larger molecules adsorb less rapidly and, consequently, their foamabilities are weaker^77^. Generally, esters embodying the shorter acyl chain length (dodecanoyl, C_12_) display superior foamabilities, attaining 36 mm, 52 mm, and 100 mm for esters incorporating glycine, valine, and cysteine (1 wt%.) at 25 °C, respectively as shown in Fig. 11. Further, those bearing unsaturated acyl chain length with one double bond have no significant effect on their foamabilities. Specifically, compound **28,** which possesses a 12-hydroxy-9-octadecenoyl side-chain length, exhibits the highest foam (100 mm) in the glycine homologous series. Excellent foamabilities were also recognized in all candidates of the surfactant series bearing cysteine (compounds **34, 35,** and **38),** which produce foams reaching 115 mm, 160 mm, and 109 mm, respectively, compared to SDS (180 mm), (1 Wt%.) at 40 °C as shown in Fig. 11. As expected, increasing temperature enhances foam formation, as presented in Fig. 11. In general, foam stability varies significantly with the carbon chain length of the hydrophobic part and the structure of the amino acid. The foam stability is measured by the decrease in foam height on standing from the time of foam generation (zero time) until the foam height that remains on standing for 30 min. The stability of foam is enhanced by surfactant adsorption at the air/water interface and the transmission rate of air bubbles to the bulk solution^77^. The results reported here suggest that these glucose esters bearing glycine, valine, or cysteine may act as promising surfactants. They furnish great and varying potential that may be suitable as foaming agents in the food manufacturing and detergent industries and possibly other applications.

### Wetting

Wetting and other peculiar phenomena are among the critical properties that most surfactants exhibit and are of great value in many industrial processes^78–80^. Herein, wetting properties as determined by the Draves-wetting test^81^ for these series of (*N*-fatty acyl amino)-glucosyl esters are shown in Table 2. The data reveal that all candidates display practical wetting ability at 0.01% and 0.1% (w/w) concentrations compared to distilled water as a control (194 s). Their effectiveness is not readily significant at lower (0.001%) concentration. As expected, the rewet time is considerably shorter than wetting times. The same trend was observed with water rewetting (96 s). In general, wetting ability, surface tensions, and hydrophile–lipophile balance (HLB) are all related to molecular structure and ascertain the most proper configuration concerning this specific function. For instance, the hydrophobic acyl side-chain determines, to a large extent, its water solubility, as indicated by HLB values. Thus, shorter acyl chain length renders an ester more hydrophilic, consequently increasing its solubility in water, thereby enhancing its wetting capability. The structure of the acyl side-chain obviously, has varying effects on the wetting efficiency of these compounds. Draves-wetting properties of these esters bearing shorter side-chain length (12 carbons) display attractive wetting capability. The dodecanoyl esters resulted in a Draves-wetting time of the cotton skein of around 65 s at a 0.1% (w/w) solution of the ester bearing glycine. Thus, wetting time decreases as the chain length of the fatty acyl substituent becomes shorter (from C_16_ to C_12_).

**Table 2:** Wetting time of different *N*-fatty acyl glucopyranosyl esters **24–38** at pH 6.5 and 7.5.

Samples no.	0.1 %		0.01 %		Peatmoss 0.1 %	
	Wetting sec. pH 6.5/pH 7.5	Re wetting sec. pH 6.5/pH 7.5	Wetting sec. pH 6.5/pH 7.5	Re wetting sec. pH 6.5/pH 7.5	Wetting sec. pH 6.5/pH 7.5	Re wetting sec. pH 6.5/pH 7.5
Dis. H_2_O	194	96			24 sec	
SDS	13	7	15	8	6	4
24	65/35	32/18	66/43	33/19	9/8	3/2
25	71/38	35/19	74/44	36/20	15/13	4/5
26	74/39	38/20	75/46	39/21	16/14	5/6
27	75/43	39/24	76/47	41/25	17/8	6/4
28	50/38	25/19	52/43	26/20	17/7	5/4
29	59/33	25/17	67/43	33/18	12/7	3/2
30	60/34	27/18	69/44	35/19	13/8	4/3
31	61/37	29/20	73/46	37/22	15/9	5/4
32	64/40	31/21	75/48	39/23	16/11	4/5
33	46/36	22/19	67/41	31/21	14/10	5/4
34	47/32	23/18	49/42	25/19	9/7	3/2
35	49/35	28/19	52/45	30/21	10/8	4/3
36	59/39	29/21	60/47	31/22	12/9	5/3
37	62/41	30/22	63/49	33/24	14/9	5/4
38	48/34	24/20	50/44	25/21	13/8	4/3

Further, regarding the different combinations of structures may affect wetting ability, it is important to investigate the impact of the amino acid linker (glycine, valine, and cysteine) on changing wetting properties. Thus, the effect of introducing an amino acid into the glucosyl ester molecules was suggested to represent an efficient approach, probably by enhancing the surface activity of these esters through structure modification. For instance, the effect of introducing valine as a linker between the hydrophobic acyl chain and the glucose head group significantly improves the wetting efficiency of this molecular framework compared to those esters bearing glycine. Once again, the incorporation of cysteine in the mentioned glucosyl esters further significantly increases the wetting capabilities of this series, which may be attributed to the hydrophilic nature of the methyl thiol (–CH_2_SH) group of the amino acid residue by increasing the hydrophilic character of these molecular structures and consequently the solubility and wetting potentiality. Therefore, considerable changes in Draves-wetting behavior may follow slight variations in the structure of the substrates.

Furthermore, the results in Table 2 reveal that varying relations exist between the wetting ability and acyl chain length of the hydrophobic moiety. Moreover, congeners bearing a 12-hydroxy-octadecenoyl side-chain display the most efficient wetting ability, regardless of the amino acid binder. The excellent wetting efficiency for this type of molecular structure, in which a hydroxyl group is located at the center of the hydrocarbon chain, is consistent with fulfilling the functionality of a satisfactory wetting agent. It has been established that wetting characteristics are created when a hydrophilic group such as (OH) occurs at or near the center of a hydrophobic chain^43^. The data also show that wetting time is greatly enhanced at pH 7.5 compared to pH 6.5, with shorter times recorded in wetting and rewetting values.

Notably, the most prominent single uses of wetting agents are in the textile industry, for example, as assistants to enable dyestuffs to be easily and evenly adsorbed by the textile fibers, as leveling agents in mercerization, and as other commercial finishing treatments to provide rapid and uniform action. Furthermore, it is a common practice to add water-soluble surfactants to insecticide sprays to improve spreading and covering on plant surfaces. Additionally, wetting agents are of value in cleaners, polishes, and cosmetics to control their roles in some consumer products. On the other hand, water-repellent soils, which are hydrophobic, occur worldwide and are generally treated with conditioners to enhance water infiltration. The early literature, cited for the use of some organic compounds as soil conditioners, was reported by Micich et al.^82^. Later, this application of surface active agents gained further considerable importance^82^. The utility of these compounds to soils was found to develop growth and enhance their performance properties by increasing water absorption. Moreover, the literature includes different reports on surfactants as soil for facilitating fertility and growth, with little publication relating wetting properties to chemical structure^82,83^. Accordingly, the drop penetration test was examined to evaluate the possible employment of the tittle esters in soil treatment and remediation.

In this work, the data obtained on the potentiality of wetting by the drop-penetration method is found to follow the same trend as those determined by the Draves test on cotton skeins (Table 2). Thus, the drop penetration test used in this study consistently confirms Drave's wetting data, which occurs even with water control. Wetting properties are related to the acyl-chain length of the *N*-acyl glucose ester structure. However, values obtained for wetting ability by the drop penetration test for peatmoss (which is a solid porous substance) are less than those determined by the Draves-wetting skein procedure, which is because wetting of soil involves first wetting of the soil particles and then percolation from this porous substrate, even though both the Draves test and drop penetration data follow the same trend, as mentioned previously. Thus, the different congeners of these homologous series provide different opportunities to find and select the proper and favorable product to attain the specific purpose. Therefore, current studies offer consistent and promising data, helping to study the mechanism of surfactant solution's percolation in relation with molecular structure. In this work, these nonionic *N*-acyl glucosyl esters' attractive properties suggest the feasibility of using different organic substrates of the esters to reach the appropriate wetting for different industrial uses.

### Emulsification properties

The glucosyl esters of this study possess an amphiphilic structure, which arises from the occurrence of hydrophilic hydroxyl groups associated with the glucose moiety, together with a hydrophobic acyl side-chain within the same molecule, which imparts peculiar surface-active properties to this type of molecule. So, this molecular structure reduces immiscible liquids' surface and interfacial tension, facilitating emulsions' formation by generating finely dispersed droplets^84,85^. The net result of the hydrophilicity or lipophilicity of these molecules is often indicated by the hydrophile-lipophile balance (HLB), which is a coding scale ranging from 0 to 20, where a high HLB has values above 11 and a low HLB is below 9. Accordingly, in the present work, the ability of emulsifying, in relation to HLB values was assessed to examine oil/water emulsification and to ensure emulsion stabilization.

The capability of emulsification, together with the time for the persistence and stability of the formed emulsion, are illustrated in (Tables 3 and 4). The results demonstrate important practical issues when an emulsifier is newly developed, especially in food, cosmetics, and pharmaceutical products, where it is required to avoid phase separation^86^. These three homologous series of glucosyl esters (Table 3) consist of surfactant products with HLB values ranging between 8.84 and 12.27, suggesting their possible utility in various practical applications. The esters prepared during this study display excellent emulsifying abilities with varying stability of the produced emulsions. Meanwhile, their HLB values (9–12.27) indicate their hydrophilicity and may serve as oil in water-type emulsifiers^87^. As shown in Table 3, esters embodying longer chain lengths have lower HLB values.

**Table 3:** Emulsion stability of *N*-fatty acyl amino glucopyranosyl esters at pH 6.5.

Compounds	Emulsion stability (ml)																			HLB value
	Min				Hour			Days												
	0	5	15	30	1	2	5	1	2	5	8	9	10	12	14	15	16	17	18	
24	5	5	5	3	2.5	2	1.5	1	Dis											12.27
25	5	5	5	4.5	4	3	3	3	2.5	1.5	Dis									11.32
26	5	5	5	4	3.5	3	2	2	2	1	0.5	Dis								10.37
27	5.5	5.5	5.5	5.5	5.5	5	5	4	3.5	2	1.5	1	1	1	Dis					9.42
28	5.5	5.5	5.5	5.5	5.5	5	4.5	4	3.5	3	2.5	2.5	2.5	2.5	0.5	Dis				11.09
29	5.5	5.5	5.5	5.5	5.3	5	3	2.9	3.5	Dis										10.85
30	5.5	5.5	5.5	5.5	5.2	4.5	3	3	3	1.5	1.5	1.5	Dis							9.99
31	5.5	5.5	5.5	5.5	5.5	5.3	5.3	4	4	2	2	2	1.5	Dis						8.95
32	5.5	5.5	5.5	5.5	5.5	5.2	5	4	4	2.5	2.5	2.5	2	1	1	Dis				8.84
33	5.5	5.5	5.5	5.5	5.5	5	4.5	4	4	2	2	2	1.5	1	1	0.5	Dis			9.67
34	5.5	5.5	5.5	5.5	5.5	5.5	5.5	5	4	2.5	Dis									11.38
35	5.5	5.5	5.5	5.5	5.5	5.5	5.4	5.2	4	3	3	2	2	Dis						10.72
36	6	6	6	6	5.9	5.8	5.5	5.3	4.2	3	3	3	3	1.5	Dis					10.14
37	5.5	5.5	5.5	5.5	5.5	5.5	5	4	4	3	2.5	2.5	2.5	2	1	1	1	Dis		9.65
38	5.5	5.5	5.5	5.5	5.5	5.5	5	5	5	4	3	3	3	2.5	2	2	2	1	Dis	8.84

**Table 4:** Emulsion stability of *N*-fatty acyl amino glucopyranosyl esters at pH 7.5.

Compounds	Emulsion stability (ml)																					
	Min				Hour			Days														
	0	5	15	30	1	2	5	1	4	9	13	16	38	43	45	48	49	50	52	60	62	63
24	5.5	5.5	5.5	5.5	5.5	5.3	5	4	3.5	2	Dis											
25	5.5	5.5	5.5	5.5	5.5	5.5	5.5	4.5	3	3	2.5	2.5	Dis									
26	5.5	5.5	5.5	5.5	5.5	5.3	5	4	3	3	2.5	2	0.5	Dis								
27	5.5	5.5	5.5	5.5	5.5	5	5	4	4	3.5	2.5	2	1	0.5	0.5	Dis						
28	5.5	5.5	5.5	5.5	5.5	5	5	4.5	3.5	3.5	3	2.5	2	1	1	0.5	Dis					
29	5.5	5.5	5.5	5.5	5.5	5.5	4	3	2.5	2	1	Dis										
30	5.5	5.5	5.5	5.5	5.5	5.5	4.5	4.5	3	3	3	2	0.5	0.5	Dis							
31	5.5	5.5	5.5	5.5	5.5	5.3	5	4.5	4	3	2.5	2	1	1	1	Dis						
32	5.5	5.5	5.5	5.5	5.5	5.5	5.5	5	3.5	3	2.5	2	1	1	1	0.5	0.5	Dis				
33	5.5	5.5	5.5	5.5	5.5	5.5	5	4.5	3.5	3	2	2	2	2	2	2	1	1	Dis			
34	5.5	5.5	5.5	5.5	5.5	5.5	5.5	4	3.5	3	2	2	1.5	Dis								
35	5.5	5.5	5.5	5.5	5.5	5.3	5	5	4.5	4	3.5	3.5	1	1	1	Dis						
36	5.5	5.5	5.5	5.5	5.5	5.5	5.5	5	4.5	3.5	2	2	0.5	0.5	0.5	0.5	0.5	0.5	0.5	Dis		
37	5.5	5.5	5.5	5.5	5.5	5.5	5	4.5	3.5	3.5	3	3	2	1.5	1.5	1.5	1.5	1.5	1.5	1	Dis	
38	5.5	5.5	5.5	5.5	5.5	5.5	5	5	3.5	3	2.5	2.5	2	1.5	1.5	1.5	1.5	1.5	1.5	1	0.5	Dis

*S.D.S* Sodium dodecyl sulfate control Dis.: 50 day^88^, *Dis.* disappearance.

Overall, the HLB values of glucosyl esters bearing glycine decreased from 12.27 to 10.37 with increasing acyl chain length from 12 to 16 C. Again, those with valine or cysteine amino acid linkers followed the same trend as the HLB decreased from 10.85 to 8.95 and from 11.38 to 10.14, respectively. Further, the structure of the acyl chain significantly affects the HLB and emulsifying properties of the corresponding esters. Thus, unsaturated acyl side chains such as octadecenoyl or hydroxy-octadecenoyl attain slightly higher HLB. The results of storage reveal that increasing the hydrophobic side-chain enhances the stability of the derived emulsion, as indicated by the persistence of the emulsion in some cases for very long periods, reaching up to 63 days as in the case of compound **38** compared with 50 days for (SDS)^88^.

It is noteworthy that, although the different esters prepared in this study possess remarkably stable emulsifying properties, it was noticed that members containing a cysteine amino acid, especially those having octadecenoyl or hydroxy-octadecenoyl side-chains in the ester molecule, showed better emulsifying potentiality (compounds **34–38)**. This result may be attributed to increased preferential solubilization of these molecules in the oil phase and some molecular association of the surfactant ester at the interface. Furthermore, the efficiency of stabilizing emulsions is greater at pH 7.5 than at pH 6.5.

In conclusion, the newly modified esters' molecular structure offers different meaningful opportunities to obtain a tunable range of surface activities by selecting the target structure–property profiles. As a result, a more significant number of products may be available for satisfying diverse needs in a wide range of food, cosmetics, pharmaceuticals, and other industrial products, not least because of their broad range of HLB values but also for their nontoxic nature and their biodegradation into readily digestible products.

### Biodegradation

Biodegradation is the breakdown of organic materials into more ecologically friendly molecules like water, carbon dioxide, and biomass by using naturally available living microorganisms (bacteria and fungi) under normal environmental conditions, eventually reintroducing the molecules into the environment. In this work, the progression of biodegradation was monitored by measuring the rise in surface tension as a function of time^89^ (Table 5). The biodegradation rate of the newly synthesized sugar esters was compared with that of sodium dodecyl sulfate (SDS) as a control. The results showed that complete loss of surface activity for the prepared glucosyl esters was attained within 6–7 days, demonstrating the accomplishment of the time required for biodegradation. The biodegradation rate shows no significant difference in the biodegradation period among the various members of these homologous series.

**Table 5:** Biodegradability of different *N*-fatty acyl amino glucopyranosyl esters **24–38.**

Sugar	*N*-fatty acyl amino acids	Days							
		1	2	3	4	5	6	7	8
R. W		71	71	71	71	71	71	71	71
S.D. S		41	48	57	62	69	71	71	71
Glucose	*N*-dodecanoyl glycine **24**	32	39	45	50	55	60	68	68
	*N*-tetradecanoyl glycine **25**	30	38	43	49	56	61	69	69
	*N*-hexadecanoyl glycine **26**	27	39	44	51	57	60	68	68
	*N*-octadecenoyl glycine **27**	38	45	51	58	67	71	71	71
	*N*-12-hydroxy-9-octadecenoyl glycine **28**	39	44	52	57	61	69	69	69
	*N*-dodecanoyl valine **29**	35	42	49	59	65	71	71	71
	*N*-tetradecanoyl valine **30**	34	44	50	60	66	70.5	70.5	70.5
	*N*-hexadecanoyl valine **31**	32	40	46	53	64	71	71	71
	*N*-octadecenoyl valine **32**	33	41	52	58	65	70	70	70
	*N*-12-hydroxy-9-octadecenoyl valine **33**	31	39	47	56	64	70.5	70.5	70.5
	*N*-dodecanoyl Cysteine **34**	33.5	41	50	58	63.5	70	70	70
	*N*-tetradecanoyl Cysteine **35**	34	43	52	59	65.5	71	71	71
	*N*-hexadecanoyl Cysteine **36**	33	39	49	54	66	71	71	71
	*N*-octadecenoyl Cysteine **37**	36	40	51	59	65	70.5	70.5	70.5
	*N*-12-hydroxy-9-octadecenoyl Cysteine **38**	39	45	48	58	61	71	71	71

*R.W* River water, *S.D.S* Sodium dodecyl sulfate control

### Antimicrobial effectiveness

Several investigations have studied the antibacterial action of carbohydrate ester derivatives, a phenomenon that can be utilized to maintain nutritional substances^90^. The mechanism of the antibacterial action was generally attributed to the rupture of microbial cell membranes^91^. The present study evaluated the relationship between the structure of the sugar esters and their antimicrobial potential. Accordingly, the inhibition zone diameter using the agar well diffusion method (Table 6), as well as the minimum inhibition concentrations (MIC) using the broth dilution method (Table 7) for these types of glucosyl esters were tested against pathogenic Gram-positive and Gram-negative bacteria as well as two fungi species. Parallel experiments were run on *Penicillin* *G* and *Ciprofloxacin* as standard Gram-positive and Gram-negative antibacterial agents, respectively, and the *ketoconazole* antifungal agent as a control. The results in Table 6 reveal that all microorganisms studied were significantly inhibited by the surfactants examined. All glucosyl esters tested had inhibition zones ranging from 6.67 ± 0.58 to 16 ± 1.00 mm for all types of bacteria and from 6.33 ± 1.16 to 14.0 ± 2.00 mm for fungi, respectively, which prove their antimicrobial activity^8^. For instance, compound **33** showed the greatest inhibition effect against *Gram* + *ve* *B.* *cereus*. Moreover, **24, 27, 32, 38,** and **32** had relatively high inhibition effects but less than **33.** However, compounds **28, 29,** and **34** displayed relatively lower inhibition potency against this *Gram* + *ve* microorganism. In addition, *Staph.* *aureus* was particularly inhibited by compounds **27, 29, 32, 34,** and **38*.*** On the other hand, the *Gram-ve* *E.* *coli* and *E.* *cloacae* were significantly susceptible to compounds **24, 27, 28, 29, 37,** and **38**. Furthermore, fungal growth was inhibited by most of the tested compounds. Surfactants **28, 33,** and **38** bearing a 12-hydroxy-9-octadecenoyl acyl hydrophobic chain displayed considerable antifungal effects against *S.* *cerevisiae.* It is noteworthy that, compounds containing cysteine (**34, 37,** and **38**) are significantly active against the tested fungi which may be due to the presence of methyl thiol side chain of cysteine which is suggested to be highly reactive and hypothetically initiates the first interaction toward the bacterial cell membranes^47^. In general, **24, 27, 28, 32, 37,** and **38** may be considered broad-spectrum compounds since they are significantly effective against Gram-positive and Gram-negative tested bacteria, showing a minimum inhibition concentration between 7.81 and 62.5 µg/ml.

**Table 6:** Inhibition zone diameter (mm) of glucopyranosyl esters **24, (27–29), (32–34), 37, 38,** as well as those of controls *Penicillin* *G,* *Ciprofloxacin,* *Ketoconazole,* *against* some selected bacteria and fungi. Value = mean ± SD, *n* = 3.

Glucosyl esters	Gram (+) bacteria		Gram (−) bacteria		Fungi	
	*B.* *cereus*	*Staph.* *aureus*	*E.* *coli*	*E.* *cloacae*	*S.* *cerevisiae*	*C.* *albicans*
Inhibition Zone diameter (mm)						
** 24**	11.33 ± 1.53	8.0 ± 1.00	16.0 ± 1.00	13.33 ± 0.58	10.33 ± 1.53	6.33 ± 1.56
** 27**	11.67 ± 1.53	9.67 ± 1.53	12.67 ± 1.16	12.0 ± 0.00	6.33 ± 1.16	11.0 ± 2.47
** 28**	7.33 ± 1.53	8.33 ± 0.58	13.0 ± 0.00	11.0 ± 1.00	11.0 ± 0.00	12.67 ± 1.53
** 29**	8.33 ± 0.58	13.33 ± 1.53	13.0 ± 1.00	16 ± 1.00	8.0 ± 0.00	13.0 ± 3.00
** 32**	10.0 ± 1.00	10.0 ± 0.00	9.0 ± 0.00	11.67 ± 0.58	8.67 ± 2.08	10.0 ± 0.00
** 33**	15.0 ± 2.00	8.0 ± 0.00	6.67 ± 0.58	9.0 ± 0.00	14.0 ± 2.00	7.33 ± 2.08
** 34**	6.67 ± 0.58	12.67 ± 1.53	10.0 ± 1.00	10.0 ± 0.00	11.0 ± 2.65	11.0 ± 2.00
** 37**	9.67 ± 1.16	9.0 ± 2.00	11.0 ± 1.73	10.33 ± 1.53	9.33 ± 1.53	10.0 ± 0.00
** 38**	10.0 ± 0.00	11.33 ± 1.15	12.0 ± 0.00	8.0 ± 1.00	12.33 ± 2.52	11.0 ± 1.73
Penicillin G	25.0 ± 0.10	24.0 ± 0.00	–	–	–	–
Ciprofloxacin	–	–	29.0 ± 0.00	23.0 ± 0.00	–	–
Ketoconazole	–	–	–	–	22.0 ± 0.10	23.0 ± 0.00

**Table 7:** Minimum inhibitory concentrations (MIC, in μg/ml) of glucopyranosyl esters **24, (27–29), (32–34), 37, and 38** against some selected bacteria and fungi. a: Gram-positive pathogens; b: Gram-negative pathogens; c : Fungi.

MIC (µg/ml)						
Glucosyl esters samples	*B.* *cereus*^a^	*Staph.* *aureus*^a^ *(ATCC25923)*	*E.* *coli*^b^ *ATCC-25955*	*E.* *cloacae*^b^ *ATCC-23355*	*S.* *cerevisiae*^c^ *ATCC-9763*	*C.* *albicans*^c^ *ATCC-10231*
24	62.5	125	15.6	15.6	62.5	125
27	62.5	31.3	62.5	31.3	31.3	62.5
28	125	62.5	15.6	7.81	62.5	125
29	31.3	125	31.3	15.6	125	31.3
32	15.6	62.5	31.3	7.81	62.5	15.6
33	62.5	125	31.3	31.3	31.3	62.5
34	125	31.3	62.5	31.3	125	31.3
37	31.3	15.6	7.81	15.6	125	31.3
38	31.3	125	15.6	62.5	62.5	15.6

### Antitumor activity

**Figure 12:**
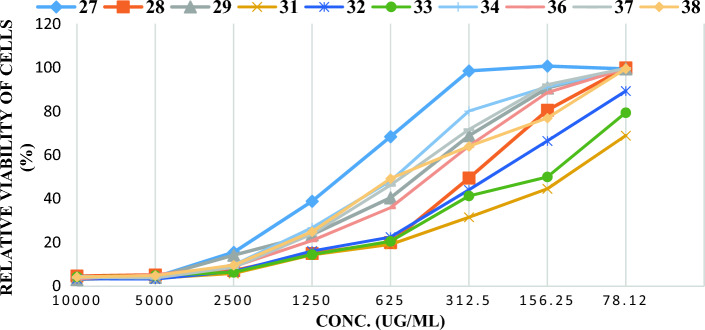
Comparison between *N*-fatty acyl amino glucopyranosyl esters and mean relative viability of (HePG-2) cell line (%).

**Figure 13:**
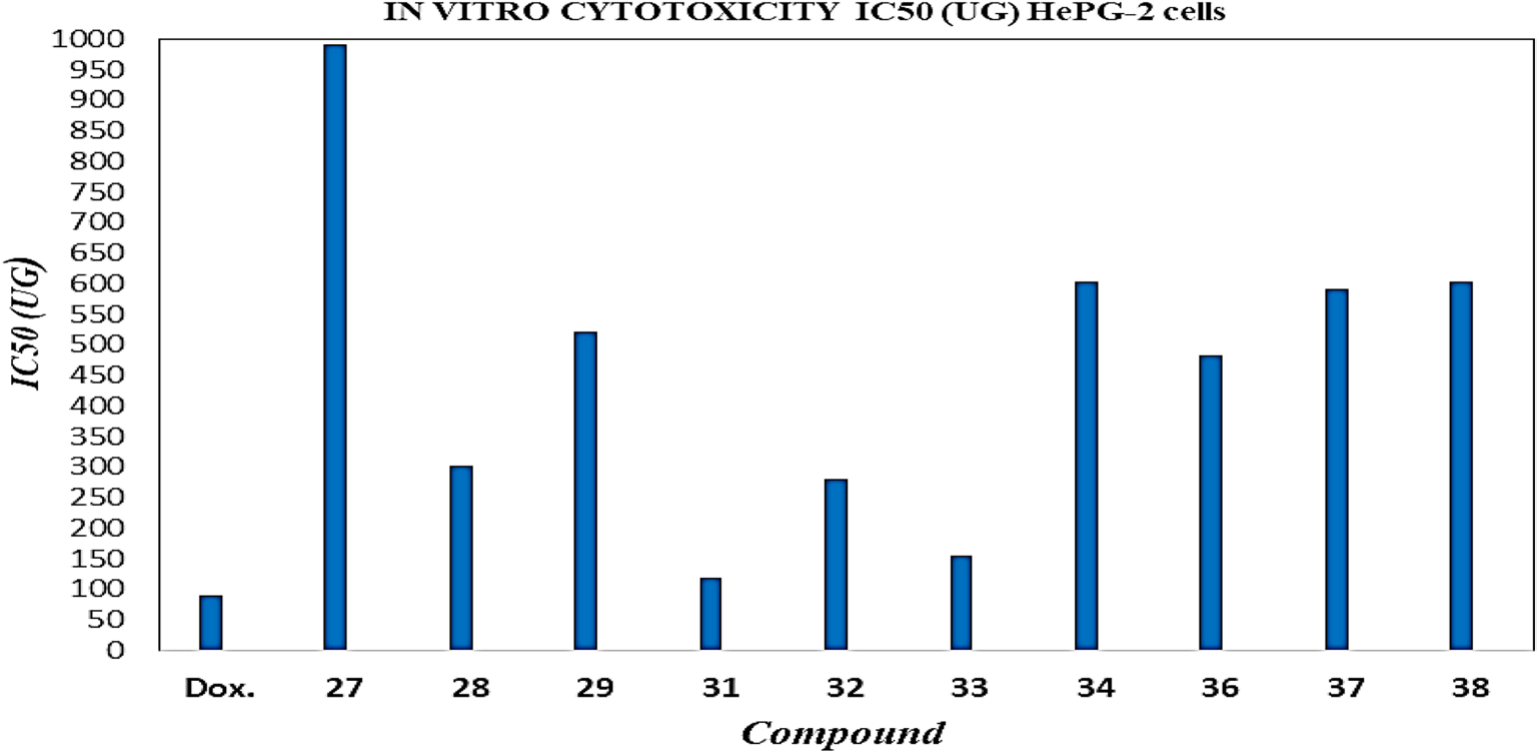
Comparison between *N*-fatty acyl amino glucopyranosyl esters and IC_50_ cancer cell lines (HePG-2).

Previous studies^92,93^ reported that some fatty acid derivatives have promising molecular structures as antitumor. Subsequently, many other studies included the antitumor and antimicrobial activities of sugar fatty acid derivatives^94,95^. Also, the inhibition of cancer cells was related to the nature of both sugar and fatty acyl chains of sugar esters^96^. Once again, many types of sugar esters, especially those of maltose^96^, lactose^40,41,97,98^, and sucrose^99,100^, display antiproliferative activities against cancer cell lines. In the current work, the studies were extended to examine the anticancer activity of the synthesized glucose fatty acyl esters bearing an amino acid component. The newly synthesized glucosyl esters are here assessed concerning their in vitro anticancer effect using 3-[4,5-dimethylthiazol-2-yl]-2,5 diphenyl tetrazolium bromide (MTT) method towards liver carcinoma (HePG-2). For comparison, doxorubicin was employed as a control anticancer drug. The half inhibitory concentration (IC_50_), calculated as the concentration that kills 50% of the cells relative to doxorubicin, is listed in Table 8, showing that all tested compounds indicate strong to moderate cell toxicity toward liver carcinoma (HePG-2). The cytotoxicity of liver carcinoma (HePG-2) is greatly affected by the concentration of these glucosyl esters (Tables 8 and 9). Thus, the non-viable cells correlate with the increased concentration of the tested compounds (Fig. 12), consistent with glucose esters exhibiting anticancer activity in a dose-and time-dependent fashion. For instance, compounds **31** and **33** strongly inhibit cancer cells, while compounds **28** and **32** are less effective. On the other hand, compounds **36, 29, 37, 38**, and **34** display comparatively moderate potentialities, whereas compound, **27** exhibited the weakest inhibitory effect. Figure 12 illustrates the relationship between the tittle esters and mean relative viability of (*HePG-2*) cell line (%) and Fig. 13 represent the effect of these esters on the in vitro IC_50_ cancer cell lines (*HePG-2*). The mode of action of the possible cytotoxic route was believed to be disrupting the cancer cell membranes, which occurs in a detergency mechanism^90^. The antitumor effect has been suggested to be due to esters themselves, rather than their metabolites^99^. It was also believed that the anti-cytotoxic effect may be attributed to the chemical molecular structure and the framework of the antitumor agent, since the structure–activity relationship analysis also suggests that lipophilic properties may be essential parameters affecting their anticancer activity^71^.

**Table 8:** IC_50_ of *N*-fatty acyl amino glucopyranosyl esters against liver carcinoma (HePG-2) cell.

NO	Comp	In vitro cytotoxicity IC_50_ (μg)
		HePG-2
–	DOX	88.75 ± 0.3
1	27	990.86 ± 6.30
2	28	300.90 ± 6.49
3	29	524.68 ± 3.07
4	31	118.17 ± 2.74
5	32	279.20 ± 3.91
6	33	153.69 ± 4.65
7	34	600.32 ± 16.68
8	36	482.63 ± 4.64
9	37	587.62 ± 6.15
10	38	603.79 ± 6.77

**Table 9:** The mean relative viability of cells (%) of *N*-fatty acyl amino glucopyranosyl esters for (HePG-2) cell line.

HePG-2								
Compounds	Conc. (μg) × 10^2^							
	100	50	25	12.5	6.25	3.125	1.56	0.78
DOX	23.00	63.89	165.39	321.65	563.7	857.6	977.36	988.68
27	3.63	4.17	15.5	38.82	68.37	98.46	100.66	99.34
28	4.67	5.16	7.13	14.99	19.98	49.42	80.45	99.84
29	3.07	4.12	14.17	23.56	40.42	68.81	90.66	99.39
31	3.62	3.57	5.82	14.33	19.11	31.52	44.54	68.86
32	3.40	3.24	6.69	16.09	22.46	44.04	66.44	89.24
33	4.28	4.88	6.37	14.49	20.53	41.29	49.97	79.35
34	4.49	4.18	9.65	26.75	48.09	80.26	91.14	99.26
36	3.40	5.27	8.89	20.86	35.91	64.08	88.52	99.51
37	4.00	3.73	8.62	23.88	46.24	71.66	72.14	99.62
38	4.23	4.88	9.61	24.93	49.20	63.97	76.88	99.45

## Conclusions

The production of industrial and nutritional surface-active agents remains an essential subject. Generally, the molecular structure of conventional sugar esters is still a problem that restricts their use in some applications. Consequently, this study aimed to synthesize novel modified chemical molecular structures of sugar esters using low-coast renewable substrates. Herein, this work is concerned with the design and synthesis of three novel homologous series of several *O*-(*N*-fatty acyl-amino acid)-d-glucosyl esters. The synthesized esters have a great potential to reduce the surface tension of solutions in which they are dissolved. Their CMC values indicate that they are promising in determining the appropriate concentrations at which this surfactant can solubilize insoluble compounds. All targeted compounds display efficient surface-active performance, including emulsification, foaming, and wetting capabilities. In the textile industry, assistants and other functions are the most prominent single uses of wetting agents. This wetting potentiality was evaluated using the Draves test method.

On the other hand, these esters were evaluated as wetting agents for hydrophobic soils using the drop-penetration method. The attractive properties of these nonionic *N*-acyl glucosyl esters suggest their feasibility in using different organic substrates of the synthesized esters to reach the appropriate wetting for different industrial uses. This unique structure of these esters makes them unaffected by Ca^2+^ and Mg^2+^ ions. Furthermore, the title esters are mainly characterized by their excellent capability of emulsifying immiscible liquids. Additionally, the storage results reveal that increasing the chain length of the esters enhances emulsion stability for long periods, reaching up to 63 days without any phase separations.

The recognition of the biological activity of sugar esters was extended to evaluate the esters in this work for their antibacterial and anticancer activities. The results demonstrate various antibacterial activities towards several human pathogenic Gram-positive and Gram-negative bacteria and some selected fungi. Additionally, they display significant antitumor properties against HePG-2 cell lines. Most effective compounds exhibit IC_50_ values of 118.17, 153.69, and 279.20 μg. These esters are promising agents for inhibiting tumor growth and acting as potential anticancer agents.

According to the previous results, evaluating the structure–property profile established through the present work should provide useful guides for developing and applying these modified sugar esters. Their functional characteristics may serve as novel emulsifiers, foam producers, and surface-active performance in food, cosmetics, and other valuable applications.

### Supplementary Information

Supplementary Information 1.

Supplementary Information 2.

## Data Availability

The data used and analyzed during the current study are available from the corresponding author upon reasonable request as long as the request does not compromise intellectual property interest. All data supporting this study's findings are available in the ESI data of this article.

## Abbreviations

24: 6-*O-*(*N*-Dodecanoyl glycine)-glucopyranose
25: 6-*O-*(*N*-Tetradecanoyl glycine)-glucopyranose
26: 6-*O-*(*N*-Hexadecanoyl glycine)-glucopyranose
27: 6-*O-*(*N*-9-Octadecenoyl glycine)-glucopyranose
28: 6-*O-*(*N*-12-Hydroxy-9-octadecenoylglycine)-glucopyranose
29: 6-*O-*(*N*-Dodecanoyl valine)-glucopyranose
30: 6-*O-*(*N*-Tetradecanoyl valine)-glucopyranose
31: 6-*O-*(*N*-Hexadecanoyl valine)-glucopyranose
32: 6-*O-*(*N*-9-Octadecenoyl valine)-glucopyranose
33: 6-*O-*(*N*-12-Hydroxy-9-octadecenoylvaline)-glucopyranose
34: 6-*O-*(*N*-Dodecanoyl cysteine)-glucopyranose
35: 6-*O-*(*N*-Tetradecanoyl cysteine)-glucopyranose
36: 6-*O-*(*N*-Hexadecanoyl cysteine)-glucopyranose
37: 6-*O-*(*N*-9-Octadecenoyl cysteine)-glucopyranose
38: 6-*O-*(*N*-12-Hydroxy-9-octadecenoyl cysteine)-glucopyranose
CMC: Critical micelle concentration
HLB: Hydrophile-lipophile balance
SDS: Sodium dodecyl sulfate
MIC: Minimum inhibitory concentration
