# Supplementary material for: Synthesis of homologous series of surfactants from renewable resources, structure–properties relationship, surface active performance, evaluation of their antimicrobial and anticancer potentialities
Source: Sci Rep. 2024 Jun 8;14:13201. doi: 10.1038/s41598-024-62905-3 (PMC11162424; doi:10.1038/s41598-024-62905-3)
Supplement: Supplementary file 2 — Supplementary Information 2. [file 41598_2024_62905_MOESM2_ESM.pdf]

Supplementary Materials

methods:

Biological activity:

Minimum Inhibitory Concentration Method (MIC) is defined as the lowest concentration of an antimicrobial that inhibits the visible growth of a microorganism after overnight incubation. The MIC of the synthesized GS was evaluated by broth dilution method. Suspensions of the tested strains were prepared (half McFarland), then serial dilutions of the stock solution of each GS were made ranging from 1000 µg/ml to 1.95 µg/ml. An equal volume of the prepared microbial suspension was added to each tube. Penicillin G and Ciprofloxacin were used as positive control. The lowest concentration (highest dilution) of each GS in the tubes with no visible microbial growth, indicated by the absence of turbidity, was regarded as the MIC.

Construction Explanation

N-dodecanoyl glycine 9 a

White powder solid 80.5% yield ^{1}H-NMR (400 MHz, DMSO-d6, δ/ppm) 0.84 (3H, C**H**$_3$); 1.22(16H, C$_8$**H**$_{16}$); 1.58 (2H, C**H**$_2$CH$_2$CO); 2.22 (2H, CH$_2$ C**H**$_2$CO); 4.06 (2H, NHC**H**$_2$CO); 7.16 (1H, N**H**-); 11.45(1H, - COO**H**).

N-tetradecanoyl glycine 10 a

White powder solid 77.5% yield ^{1}H-NMR (400 MHz, DMSO-d6, δ/ppm) 0.86 (3H, C**H**$_3$); 1.29(20H, C$_{10}$**H**$_{20}$); 1.54 (2H, C**H**$_2$CH$_2$CO); 2.24 (2H, CH$_2$ C**H**$_2$CO); 4.05 (2H, NH C**H**$_2$CO); 7.5 (1H, N**H**-); 11.60(1H, - COO**H**).

N-9-octadecenoyl glycine 12a

White semi- solid 76.2% yield ^{1}H-NMR (400 MHz, DMSO-d6, δ/ppm) 0.86 (3H, C**H**$_3$); 1.28 (20H, C$_{10}$**H**$_{20}$); 1.47 (2H, C**H**$_2$CH$_2$CO); 2.16 (4H, C**H**$_2$CH=CHC**H**$_2$); 2.24 (2H, -C**H**$_2$CO); 3.69 (2H, C**H**$_2$-NH-);5.33) 2H, C**H**=C**H**); 8.02 (1H, N**H**-); 12. 09 (1H, - COO**H**).

N-12-hydroxy-9-octadecenoyl glycine 13a

White semi- solid 74.3% yield ^{1}H-NMR (400 MHz, DMSO-d6, δ/ppm) 0.85 (3H, C**H**$_3$); 1.29(16H, C**H**$_2$ chain);1.49 (2H, CH=CHCH$_2$CHOH C**H**$_2$); 1.61(2H, -C**H**$_2$CH$_2$CO), 1.94(2H, C**H**$_2$CH=CH CH$_2$); 2.22(2H,-C**H**$_2$CO); 2.27(2H, CH=CHC**H**$_2$CHOH);3.77(1H, CH$_2$C**H**OH); 4.01(2H,COC**H**$_2$NH-); 5.46(2H, C**H**=C**H**); 6.23 (1H, -CHO**H**); 7.99 (1H, -N**H**-); 11.99 (1H, -COO**H**).

N-dodecanoyl valine 14 a

White powder solid 81.3% yield %; ^{1}H NMR (400 MHz, DMSO-d6, δ/ppm) 0.86 (3H, C**H**$_3$); 0.93 (6H, (C**H**$_3$)$_2$); 1.3 (16H, C$_8$**H**$_{16}$); 1.52 (2H, -C**H**$_2$CH$_2$CO); 2.00 (1H, C**H** (CH3)$_2$); 2.27(2H, -C**H**$_2$CO); 4.11 (1H, COC**H**-NH); 7.97 (1H, NH-); 12.01 (1H, - COO**H**).

N-tetradecanoyl valine 15 a

White powder solid 81.5% yield %; ^{1}H NMR (400 MHz, DMSO-d6, δ/ppm) 0.84 (3H, C**H**$_3$); 0.96 (6H, (C**H**$_3$)$_2$); 1.27 (20H, C$_{10}$**H**$_{20}$); 1.54 (2H, -C**H**$_2$CH$_2$CO); 1.90 (1H, C**H** (CH3)$_2$); 2.20(2H, -C**H**$_2$CO); 4.23 (1H, COC**H**-NH); 8.02 (1H, NH-); 12.70 (1H, -COO**H**).

N- hexadecanoyl valine 16 a

White powder solid 80.4% yield %; ^{1}H NMR (400 MHz, DMSO-d6, δ/ppm) 0.85 (3H, C**H**$_3$); 0.98 (6H, (C**H**$_3$)$_2$); 1.27 (24H, C$_{12}$**H**$_{24}$); 1.56 (2H, -C**H**$_2$CH$_2$CO); 1.96 (1H, C**H** (CH3)$_2$); 2.23(2H, -C**H**$_2$CO); 4.21 (1H, COC**H**-NH); 8.30 (1H, NH-); 12.23 (1H, - COO**H**).

N-9-octadecenoyl valine 17a

a white – semi solid (73.2 %);^{1}H NMR (400 MHz, DMSO-d6, δ/ppm) 0.82 (3H, C**H**$_3$); 0.95 (6H, (C**H**$_3$)$_2$); 1.26 (20H, C**H**$_2$ chain); 1.49 (2H, C**H**$_2$CH$_2$CO); 1.99 (4H, C**H**$_2$CH=CHC**H**$_2$); 2.01 (1H, C**H** (CH3)$_2$); 2.24 (2H, -C**H**$_2$CO); 4.21 (1H, COC**H**-NH); 5.36 (2H, C**H**=C**H**); 7.89 (1H, N**H**-);11.88 (1H, - COO**H**).

N-12-hydroxy-9-octadecenoyl valine 18 a

a white – semi solid (72.1 %);^{1}H NMR (400 MHz, DMSO-d6, δ/ppm) 0.86 (3H, C**H**$_3$); 0.95 (6H, 2xCH$_3$); 1.23(16H, C**H**$_2$ chain); 1.50 (2H, CHOH -C**H**$_2$): 1.56 (2H, -C**H**$_2$CH$_2$CO), 2.00(2H, C**H**$_2$CH=CH); 2.05(1H, -C**H**(CH3)$_2$); 2.24(2H,-C**H**$_2$CO); 2.34(2H, CH=CHC**H**$_2$CHOH); 3.75(1H, CH$_2$C**H**OH); 4.23(1H,CONHC**H**-);5.46 (2H, C**H**=C**H**); 6.23 (1H, -CHO**H**); 8.07 (1H, -N**H**-);12.30 (1H, - COO**H**).

N-dodecanoyl cysteine 19 a

a white solid (80.04 %);^{1}H NMR (400 MHz, DMSO-d6, δ/ppm) 0.86 (3H, C**H**$_3$); 1.26 (16H, C**H**$_2$ chain); 1.59 (2H, C**H**$_2$ CH$_2$CO); 1.82 (1H, S**H**); 2.10 (2H, -C**H**$_2$CO); 2.92 (2H, CHC**H**$_2$SH); 4.66 (1H, NHC**H**CH$_2$); 8.11 (1H, NH-);12.00 (1H, - COO**H**).

N-tetradecanoyl cysteine 20a

a white solid (79.11 %);^{1}H NMR (400 MHz, DMSO-d6, δ/ppm) 0.82 (3H, CH$_3$); 1.29 (20H, C**H**$_2$ chain); 1.52 (2H, CH$_2$ CH$_2$CO-);1.81(1H, S**H**); 2.26 (2H, CH$_2$CO-);2.93 (2H, CHCH$_2$SH); 4.50 (1H, NHC**H**CH$_2$); 8.30 (1H, N**H**-);12.04 (1H, - COO**H**).

N-hexadecanoyl cysteine 21 a

a white solid (79.10 %);^{1}H NMR (400 MHz, DMSO-d6, δ/ppm) 0.85(3H, CH$_3$); 1.29 (24H, C**H**$_2$ chain); 1.51 (2H, C**H**$_2$ CH$_2$CO-);1.82(1H, S**H**); 2.07 (2H, CH$_2$CO-);2.78 (2H, CHCH$_2$SH); 4.48 (1H, NHC**H**CH$_2$); 8.01(1H, N**H**-);12.10 (1H, - COO**H**).

N-9-octadecenoyl cysteine 22 a

a white – semi solid (76.7 %);^{1}H NMR (400 MHz, DMSO-d6, δ/ppm) 0.83 (3H, C**H**$_3$); 1.23 (20H, C**H**$_2$ chain); 1.53 (2H, C**H**$_2$CH$_2$CO); 1.80 (1H, S**H**); 1.99 (4H, C**H**$_2$CH=CHC**H**$_2$); 2.21 (2H, -C**H**$_2$CO); 2.97 (2H, CHCH$_2$SH); 4.65 (1H, NHC**H**CH$_2$); 5.36 (2H, C**H**=C**H**); 7.99 (1H, N**H**-);12.23 (1H, - COO**H**).

N-12-hydroxy-9-octadecenoyl cysteine 23a

a white – semi solid (74.8 %);^{1}H NMR (400 MHz, DMSO-d6,δ/ppm) 0.84 (3H, C**H**$_3$); 1.26 (16H, C**H**$_2$chain); 1.50 (2H, OHCH C**H**$_2$); 1.54 (2H, C**H**$_2$CH$_2$CO); 1.83 (1H, S**H**); 1.96(2H, C**H**$_2$CH=CH); 2.21 (2H, -C**H**$_2$CO); 2.27(2H, CH=CHC**H**$_2$CHOH); 2.99 (2H, C**H**$_2$SH); 3.81 (1H, CH$_2$C**H**OH); 4.48 (1H, NHC**H**CH$_2$); 5.42(2H,C**H**=C**H**); 5.49 (1H, -CHO**H**); 7.60 (1H.N**H**-);12.32 (1H, - COO**H**).

N-dodecanoyl glycine chloride 9 b

White semi- solid 83.20% yield ^{1}H-NMR (400 MHz, DMSO-d6, δ/ppm) 0.86 (t, 3H, C**H**$_3$); 1.22(m, 16H, C$_8$**H**$_{16}$); 1.53 (m, 2H, C**H**$_2$CH$_2$CO); 2.13 (t, 2H, CH$_2$ C**H**$_2$CO); 4.36 (d, 2H, NHC**H**$_2$CO); 7.05 (t, 1H, N**H**-).

N-tetradecanoyl glycine chloride 10 b

White semi- solid 79.14% yield ^{1}H-NMR (400 MHz, DMSO-d6, δ/ppm) 0.84 (t, 3H, C**H**$_3$); 1.26(m, 20H, C$_{10}$**H**$_{20}$); 1.58 (m, 2H, C**H**$_2$CH$_2$CO); 2.17 (t, 2H, CH$_2$ C**H**$_2$CO); 4.44 (d, 2H, NHC**H**$_2$CO); 7.32 (t, 1H, N**H**-).

N-9- octadecenoyl glycine chloride 12 b

White semi- solid 73.4% yield ^{1}H-NMR (400 MHz, DMSO-d6, δ/ppm) 0.83 (t, 3H, C**H**$_3$); 1.24 (m, 20H, C$_{10}$**H**$_{20}$); 1.53 (m, 2H, C**H**$_2$CH$_2$CO); 1.99 (m, 4H, C**H**$_2$CH=CHC**H**$_2$); 2.23 (t, 2H, -C**H**$_2$CO); 4.35 (d, 2H, C**H**$_2$-NH-);5.36 (m, 2H, C**H**=C**H**); 7.02 (t,1H, N**H**-).

N-12-hydroxy-9-octadecenoyl glycine chloride 13 b

White semi- solid 73.13% yield ^{1}H-NMR (400 MHz, DMSO-d6, δ/ppm) 0.85 (t, 3H, C**H**$_3$); 1.28(m, 16H, C**H**$_2$ chain);1.50 (m, 2H, CH=CHCH$_2$CHOH C**H**$_2$); 1.52(m, 2H, -C**H**$_2$CH$_2$CO), 1.91(m,2H,C**H**$_2$CH=CHCH$_2$); 2.20(t,2H,-C**H**$_2$CO); 2.26(m, 2H, CH=CHC**H**$_2$CHOH);3.74(m,1H, CH$_2$C**H**OH); 4.36(d,2H,COC**H**$_2$NH-); 5.48(m, 2H, C**H**=C**H**); 6.20 (d,1H, -CHO**H**); 7.10 (t,1H, -N**H**-).

N-dodecanoyl valine chloride 14 b

White semi- solid 78.06% yield %; ^{1}H NMR (400 MHz, DMSO-d6, δ/ppm) 0.87 (t,3H, C**H**$_3$); 0.92 (dd,6H, (C**H**$_3$)$_2$); 1.26 (s,16H, C_8**H**$_{16}$); 1.54 (m,2H, -C**H**$_2$CH$_2$CO); 2.08 (m,1H, C**H** (CH3)$_2$); 2.25(t,2H, -C**H**$_2$CO); 4.18 (dd,1H, COC**H**-NH); 7.38 (d,1H, NH-).

N-tetradecanoyl valine chloride 15b

White semi- solid 83.2% yield %; ^{1}H NMR (400 MHz, DMSO-d6, δ/ppm) 0.86 (t,3H, C**H**$_3$); 0.97 (dd,6H, (C**H**$_3$)$_2$); 1.27 (s,20H, C_{10}**H**$_{20}$); 1.56 (t,2H, -C**H**$_2$CH$_2$CO); 2.09 (h,1H, C**H** (CH3)$_2$); 2.26(t,2H, -C**H**$_2$CO); 4.16 (dd,1H, COC**H**-NH); 7.35 (d,1H, NH-). FT-IR (KBr, cm^{-1}): 3290.73 (NH), 2917.83(C-H), 2850.12 (C-H), 1820.38 (C=O acid chloride), 1636.12(NC=O amide).

N- hexadecanoyl valine chloride 16 b

White semi- solid 79.06% yield %; ^{1}H NMR (400 MHz, DMSO-d6, δ/ppm) 0.83 (t,3H, C**H**$_3$); 0.98 (dd,6H, (C**H**$_3$)$_2$); 1.29 (s,24H, C_{12}**H**$_{24}$); 1.57 (t,2H, -C**H**$_2$CH$_2$CO); 1.99 (h,1H, C**H** (CH3)$_2$); 2.25(t,2H, -C**H**$_2$CO); 4.17 (dd,1H, COC**H**-NH); 7.30 (d,1H, NH-). FT-IR (KBr, cm^{-1}): 3288.26 (NH), 2919.34(C-H), 2849.78 (C-H), 1823.36 (C=O acid chloride), 1635.92(NC=O amide).

N-9-octadecenoyl valine chloride 17 b

a white semi –solid (75.2 %);^{1}H NMR (400 MHz, DMSO-d6, δ/ppm) 0.85 (t,3H, C**H**$_3$); 0.96 (dd,6H, (C**H**$_3$)$_2$); 1.28 (m,20H, C**H**$_2$ chain); 1.51 (t,2H, C**H**$_2$CH$_2$CO); 1.97 (h,4H, C**H**$_2$CH=CHC**H**$_2$); 2.09 (h,1H, C**H** (CH3)$_2$); 2.23 (t,2H, -C**H**$_2$CO); 4.20 (dd,1H, COC**H**-NH); 5.30 (m, 2H, C**H**=C**H**); 7.29 (d,1H, N**H**-).

N-12-hydroxy-9-octadecenoyl valine chloride 18b

a white semi –solid (74.31 %);^{1}H NMR (400 MHz, DMSO-d6, δ/ppm) 0.83 (t,3H, C**H**$_3$); 0.95 (dd,6H, 2xCH$_3$); 1.28(m,16H, C**H**$_2$ chain); 1.51 (m,2H, CHOH -C**H**$_2$);1.59 (m,2H, -C**H**$_2$CH$_2$CO);1.95(2H, C**H**$_2$CH=CH); 2.09(h,1H, -C**H**(CH3)$_2$); 2.23(t,2H,-C**H**$_2$CO); 2.27(m,2H, CH=CHC**H**$_2$CHOH); 3.73(m,1H, CH$_2$C**H**OH); 4.15(dd,1H,CONHC**H**-);5.49 (m,2H, C**H**=C**H**); 6.20 (d,1H, -CHO**H**); 7.13 (d,1H, -N**H**-).

N-dodecanoyl cysteine chloride 19 b

a white semi –solid (84.21 %);^{1}H NMR (400 MHz, DMSO-d6, δ/ppm) 0.83 (t,3H, C**H**$_3$); 1.25 (s,16H, C**H**$_2$ chain); 1.55 (s,1H, S**H**); 1.59 (m,2H, C**H**$_2$ CH$_2$CO); 2.22 (t,2H, -C**H**$_2$CO); 2.94 (d,2H, CHC**H**$_2$SH); 4.54 (d,1H, NHC**H**CH$_2$); 7.61 (d,1H, NH-).

N-tetradecanoyl cysteine chloride 20 b

a white semi – solid (83.12 %);^{1}H NMR (400 MHz, DMSO-d6, δ/ppm) 0.85 (t,3H, CH$_3$); 1.26 (s,20H, C**H**$_2$ chain); 1.56 (s,1H, S**H**); 1.58 (m,2H, **CH$_2$** CH$_2$CO-);2.24 (t,2H, CH$_2$CO-);2.97 (d,2H, CHCH$_2$SH); 4.56 (d,1H, NHC**H**CH$_2$); 7.59 (d,1H, N**H**-).

N-hexadecanoyl cysteine chloride 21 b

a white semi – solid (82.14 %);^{1}H NMR (400 MHz, DMSO-d6, δ/ppm) 0.85(t,3H, CH_3); 1.27 (s,24H, C**H**$_2$ chain); 1.54(s,1H, S**H**); 1.57 (m,2H, C**H**$_2$ CH_2CO-);2.25 (t,2H, CH_2CO-);2.79 (d,2H, $CHCH_2SH$); 4.53 (d,1H, NHC**H**CH_2); 7.41(d,1H, N**H**-).

N-9-octadecenoyl cysteine chloride 22 b

a white – semi solid (78.71 %);^{1}H NMR (400 MHz, DMSO-d6, δ/ppm) 0.83 (t,3H, C**H**$_3$); 1.26 (m,20H, C**H**$_2$ chain); 1.54 (s,1H, S**H**); 1.58 (m,2H, C**H**$_2$$CH_2CO$); 1.98 (h,4H, C**H**$_2$CH=CHC**H**$_2$); 2.22 (t,2H, -C**H**$_2$CO); 2.96 (d,2H,CHC**H**$_2$SH); 4.54 (d,1H, NHC**H**CH_2); 5.33 (m, 2H, C**H**=C**H**); 7.59 (d,1H, N**H**-).

N-12-hydroxy-9-octadecenoyl cysteine chloride 23 b

a white – semi solid (77.18 %);^{1}H NMR (400 MHz, DMSO-d6,δ/ppm) 0.84 (t,3H, C**H**$_3$); 1.26 (m,16H, C**H**$_2$chain); 1.51 (m,2H, CH=CHCH$_2$CHOH C**H**$_2$); 1.55 (s,1H, S**H**); 1.58 (m,2H, C**H**$_2$$CH_2CO$); 1.94(m,2H, C**H**$_2$CH=CH); 2.23 (t,2H, -C**H**$_2$CO); 2.27(m,2H, CH=CHC**H**$_2$CHOH); 2.97 (d,2H, C**H**$_2$SH); 3.75 (m,1H, CH$_2$C**H**OH); 4.57 (d,1H, NHC**H**CH_2); 5.46(m,2H,C**H**=C**H**); 6.23 (d,1H, -CHO**H**); 7.60 (d,1H.N**H**-).

6-*O*-(*N*-dodecanoyl glycine)-glucopyranose 24

a white solid (95.6%); mp.: 97 -98.5 ˚C. ^{1}H-NMR (400 MHz, DMSO-d6,δ/ppm) 0.83 (3H, C**H**$_3$); 1.22(16H, (C**H**$_2$)$_8$); 1.45 (2H, C**H**$_2$$CH_2CO$); 2.17 (2H, C**H**$_2$CO); 3.47(1H, **H**-2); 3.54 (1H, **H**-4); 3.64 (1H, **H**-5); 3.69 (1H, **H**-3); 3.81 (2H,NHC**H**$_2$CO); 4.26 (2H, **H**-6); 4.48 (1H, O**H**-4); 4.59 (1H, O**H**-2); 4.76 (1H, **H**-1); 4.90 (1H, O**H**-3); 5.56 (1H, O**H**-1); 7.49 (1H,N**H**-). FT-IR (KBr, cm^{-1}): 3392.76 (NH), 3213.5 (O-H), 2959.78(C-H), 2925.85 (C-H), 1746.48 (C=O), 1629.61(NC=O), 1503.65(N-H), 1384.34(C-H), 1055.43(C-O-C), 919.25 pyranose ring.

6-*O*-(*N*-tetradecanoyl glycine)-glucopyranose 25

a white solid (86.4%); mp.: 94.5 -96°C. ^{1}H-NMR (400 MHz, DMSO-d6, δ/ppm) 0.85 (3H, C**H**$_3$); 1.22(20H, (C**H**$_2$)$_{10}$); 1.47 (2H, C**H**$_2$$CH_2CO$); 2.17 (2H, C**H**$_2$CO); 3.48(1H, **H**-2); 3.52 (1H, **H**-4); 3.62 (1H, **H**-5); 3.67 (1H, **H**-3); 3.83 (2H,NHC**H**$_2$CO); 4.23 (2H, **H**-6); 4.44 (1H, O**H**-4); 4.61 (1H, O**H**-2); 4.77 (1H, **H**-1); 4.85 (1H, O**H**-3); 5.51 (1H, O**H**-1); 7.42 (1H,N**H**-). FT-IR (KBr, cm^{-1}): 3397.15 (NH), 3251.34 (O-H), 2961.10(C-H), 2925.60 (C-H), 1717.78 (C=O), 1627.20 (NC=O), 1502.10(N-H), 1386.01(C-H), 1056.57(C-O-C), 918.48 pyranose ring.

6-*O*-(*N*-12-hydroxy-9-octadecenoylglycine)-glucopyranose 28

a white semi- solid (84.9%); mp.: 75.5 -77°C. ^{1}H NMR (400 MHz, DMSO-d6, δ/ppm) 0.86 (3H, C**H**$_3$); 1.27(16H, C**H**$_2$ chain);1.49 (2H, CH=CHCH$_2$CHOH C**H**$_2$); 1.59(2H, -C**H**$_2$$CH_2CO$), 1.9(2H, C**H**$_2$CH=CH CH_2); 2.2(2H,-C**H**$_2$CO); 2.25(2H, CH=CHC**H**$_2$CHOH); 3.45(1H, **H**-2); 3.52 (1H, **H**-4); 3.61(1H, **H**-5); 3.65(1H, **H**-3); 3.7(1H, CH$_2$C**H**OH); 3.87(2H,COC**H**$_2$NH-); 4.28(2H, **H**-6);4.42(1H, O**H**-4); 4.59(1H, O**H**-2); 4.72 (1H, **H**-1) 4.82 (1H, O**H**-3);4.93(2H, C**H**=C**H**); 4.98(1H, O**H**-1); 6.21 (1H, -CHO**H**); 7.4 (1H, -N**H**-). FT-IR (KBr, cm^{-1}): 3366.52

(N-H), 3248.82 (O-H), 2931.42 (C-H), 2857.94 (C-H), 1737.77 (C=O), 1625.53 (NC= O), 1460.97 (N-H), 1365.41(C-H), 1077.89(C-O-C), 925.91 pyranose ring.

6-*O*-(*N*-dodecanoyl valine)-glucopyranose 29

a white solid (84.2 %); ^{1}H NMR (400 MHz, DMSO-d6, δ/ppm) 0.84 (3H, C**H**$_3$); 0.95 (6H, (C**H**$_3$)$_2$); 1.26 (16H, C**H**$_2$ chain); 1.49 (2H, -C**H**$_2$CH$_2$CO); 2.1 (1H, C**H** (CH3)$_2$); 2.3(2H, -C**H**$_2$CO); 3.4 (1H, **H**-2); 3.50 (1H, **H**-4); 3.57 (1H, **H**-5); 3.66 (1H, **H**-3); 4.23 (1H, COC**H**-NH); 4.39 (2H, **H**-6); 4.42 (1H, O**H**-4); 4.67 (1H, O**H**-2); 4.81 (1H, **H**-1); 4.89 (1H, O**H**-3); 6.11 (1H, O**H**-1); 7.35 (1H,N**H**-). FT-IR (KBr, cm^{-1}): 3367.20 (N-H), 3204.39(O-H), 2937.26(C-H), 2883.47 (C-H), 1729.72 (C=O), 1664.84 (NC= O), 1605.98 (N-H), 1363.96(C-H), 1050.05(C-O-C), 925.20 pyranose ring.

6-*O*-(*N*- hexadecanoyl valine)-glucopyranose 31

a white solid (74.5 %);^{1}H NMR (400 MHz, DMSO-d6, δ/ppm) 0.95 (3H, C**H**$_3$); 0.98 (6H, (C**H**$_3$)$_2$); 1.27 (24H, C**H**$_2$ chain); 1.44 (2H, -C**H**$_2$CH$_2$CO); 2.02 (1H, C**H** (CH3)$_2$); 2.21(2H, -C**H**$_2$CO); 3.43 (1H, **H**-2); 3.51 (1H, **H**-4); 3.58 (1H, **H**-5); 3.64 (1H, **H**-3); 4.25 (1H, COC**H**-NH); 4.46 (2H, **H**-6); 4.43(1H, O**H**-4); 4.57 (1H, O**H**-2); 4.77 (1H, **H**-1); 4.90 (1H, O**H**-3); 6.23 (1H, O**H**-1); 8.42 (1H,N**H**-). FT-IR (KBr, cm^{-1}): 3369.83 (N-H), 3206.82 (O-H), 2917.77(C-H), 2849.73 (C-H), 1729.45 (C=O), 1665.07(NC= O), 1606.82(N-H), 1363.94(C-H), 1077.69(C-O-C), 925.64 pyranose ring.

6-*O*-(*N*-9-octadecenoyl valine)-glucopyranose 32

a white – semi solid (83.5 %);^{1}H NMR (400 MHz, DMSO-d6, δ/ppm) 0.83 (3H, C**H**$_3$); 0.92 (6H, (C**H**$_3$)$_2$); 1.23 (20H, C**H**$_2$ chain); 1.45 (2H, C**H**$_2$CH$_2$CO); 1.97 (4H, C**H**$_2$CH=CHC**H**$_2$); 2.1 (1H, C**H** (CH3)$_2$); 2.22 (2H, -C**H**$_2$CO); 3.45 (1H**,** **H**-2); 3.5 (1H, **H**-4); 3.55 (1H, **H**-5); 3.58 (1H, **H**-3); 4.23 (1H, COC**H**-NH); 4.39 (2H, **H**-6); 4.43 (1H, O**H**-4); 4.6 (1H, O**H**-2); 4.70(1H,**H**-1); 4.82 (1H, O**H**-3); 4.99 (2H, C**H**=C**H**); 5.35(1H, O**H**-1); 7.4 (1H, N**H**-). FT-IR (KBr, cm^{-1}): 3396.68 (N-H), 3259.33 (O-H), 2960.41(C-H), 2868.25 (C-H), 1712.87 (C=O), 1632.67 (NC= O), 1457.78(N-H), 1363.95(C-H), 1055.27(C-O-C), 921.92 pyranose ring.

6-*O*-(*N*-tetradecanoyl cysteine)-glucopyranose 35

white solid (89.3 %);^{1}H NMR (400 MHz, DMSO-d6, δ/ppm) 0.85 (3H, CH$_3$); 1.27 (20H, C**H**$_2$ chain); 1.49 (2H ,C**H**$_2$ CH$_2$CO-);1.96(1H, S**H**); 2.21 (2H,C**H**$_2$CO-);2.98 (2H,CHC**H**$_2$SH); 3.45 (1H,**H**-2); 3.5 (1H,**H**-4); 3.57 (1H,**H**-5); 3.59 (1H,**H**-3); 4.2 (2H,**H**-6); 4.3 (1H, O**H**-4); 4.46 (1H, NHC**H**CH$_2$); 4. 64 (1H, O**H**-2); 4.72 (1H, **H**-1); 4.86 (1H, **HO**-3); 4.92 (1H, O**H**-1); 6.56 (1H, N**H**-). FT-IR (KBr, cm^{-1}): 3368.71 (N-H), 3248.72 (O-H), 2919.07, 2850.55 (C-H), 2692.46 (S-H), 1730.94 (C=O), 1625.75(NC= O), 1461.99 (N-H), 1364.22(C-H), 1049.73 (C-O-C), 926.10 pyranose ring.

6-*O*-(*N*-9-octadecenoyl cysteine)-glucopyranose 37

yellowish white – semi solid (82.7 %);^{1}H NMR (400 MHz, DMSO-d6, δ/ppm) 0.86 (3H, C**H**$_3$); 1.26 (20H, C**H**$_2$ chain); 1.48 (2H, C**H**$_2$CH$_2$CO); 1.97(1H, S**H**);1.98 (4H, C**H**$_2$CH=CHC**H**$_2$); 2.22 (2H, -C**H**$_2$CO); 2.98 (2H,CHC**H**$_2$SH);3.45 (1H**, H**-2); 3.49 (1H, **H**-4); 3.54 (1H, **H**-5); 3.57 (1H, **H**-3); 4.10(2H, **H**-6); 4.39(1H, O**H**-4); 4.41 (1H, NHC**H**CH$_2$);4.52 (1H, O**H**-2); 4.70(1H,**H**-1); 4.86 (1H, O**H**-3); 5.10 (2H, C**H**=C**H**); 5.32(1H, O**H**-1); 7.10 (1H, N**H**-). FT-IR (KBr, cm^{-1}): 3430.72 (N-H), 3247.93 (O-H), 2923.22(C-H), 2851.70 (C-H), 2657.67 (S-H), 1738.79 (C=O), 1655.18 (NC= O), 1463.43(N-H), 1365.11(C-H), 1049.97(C-O-C), 926.36 pyranose ring.

Table S 1 FT-IR data of compounds **24-28**

FT-IR Data(cm^{-1})						
**Surfactant**	**(ν N–H)**	**O-H**	**ν C-H Asym.**	**ν C-H sym.**	**ν C=O Amide**	**ν C=O ester**
24	3392.76	3213.5	2959.78	2925.85	1629.61	1746.48
25	3379.15	3251.34	2961.10	2925.60	1627.20	1717.78
26	3353.33	3249.78	2921.48	2852.38	1633.00	1737.79
27	3392.18	3252.79	2926.83	2855.18	1629.30	1711.79
28	3366.52	3248.62	2931.42	2857.94	1659.96	1737.77

Table S 2 FT-IR data of compounds **29-33**

FT-IR Data(cm^{-1})						
**Surfactant**	**(ν N–H)**	**O-H**	**ν C-H Asym.**	**ν C-H sym.**	**ν C=O Amide**	**ν C=O ester**
29	3367.20	3204.39	2937.26	2883.47	1664.84	1729.72
30	3368.04	3245.54	2937.92	2884.80	1663.89	1730.05
31	3369.83	3206.82	2917.77	2849.73	1665.07	1729.45
32	3396.68	3259.33	2960.41	2868.25	1632.67	1712.87
33	3368.28	3246.70	2935.18	2863.70	1663.94	1737.46

Table S 3 FT-IR data of compounds **34-38**

FT-IR Data(cm^{-1})							
**Surfactant**	**(ν N–H)**	**O-H**	**ν C-H Asym.**	**ν C-H sym.**	**S - H**	**ν C=O Amide**	**ν C=O ester**
34	3391.17	3297.03	2920 .26	2851.26	2673.99	1622.76	1729.50
35	3368.71	3248.72	2919.07	2850.55	2692.46	1625.75	1730.94
36	3430.85	3248.13	2921.10	2850. 55	2657.96	1664.15	1738.62
37	3368.20	3247.93	2923.22	2851.70	2657.67	1655.18	1738.79
38	3432.02	3245.39	2932.33	2858.45	2555.71	1663.37	1737.98

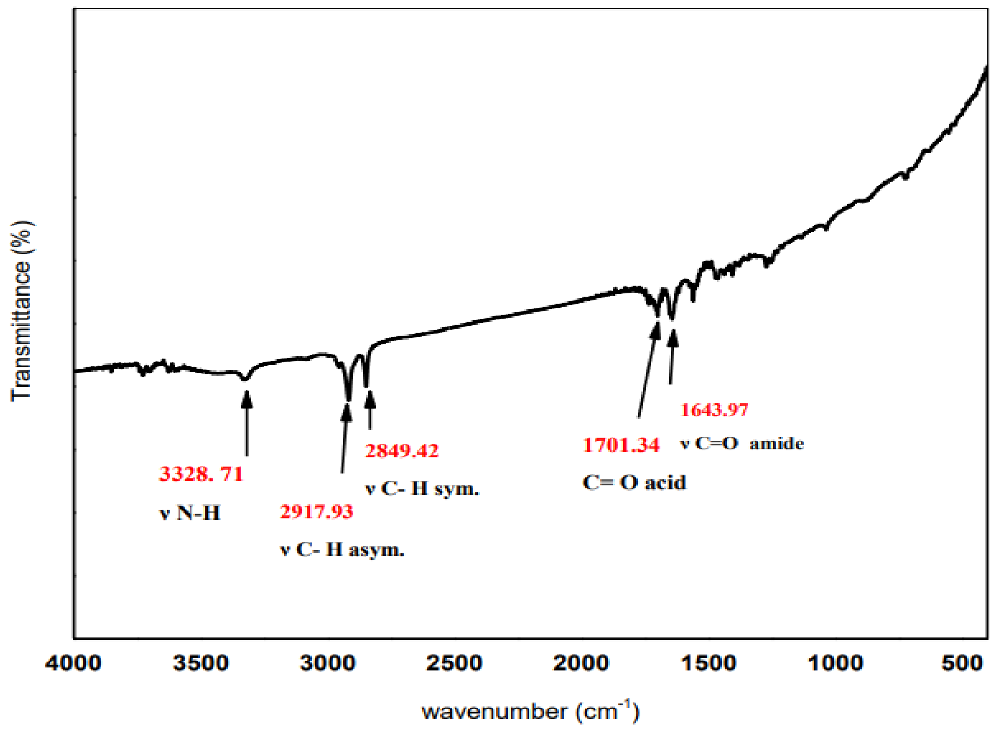

Figure S1. FT-IR Spectra for *N*- hexadecanoyl glycine.

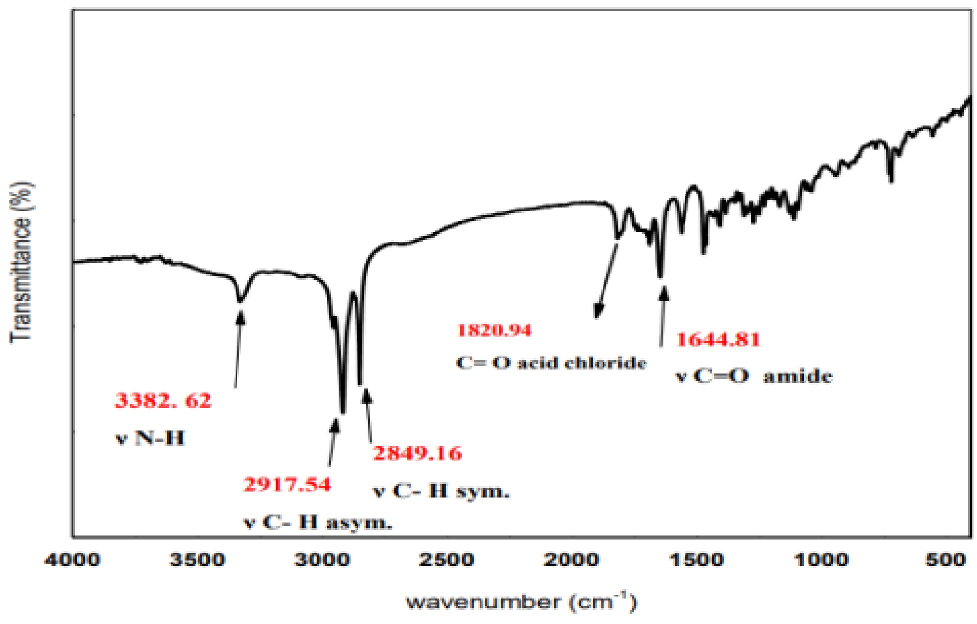

Figure S2. FT-IR Spectra for *N*- hexadecanoyl glycine chloride

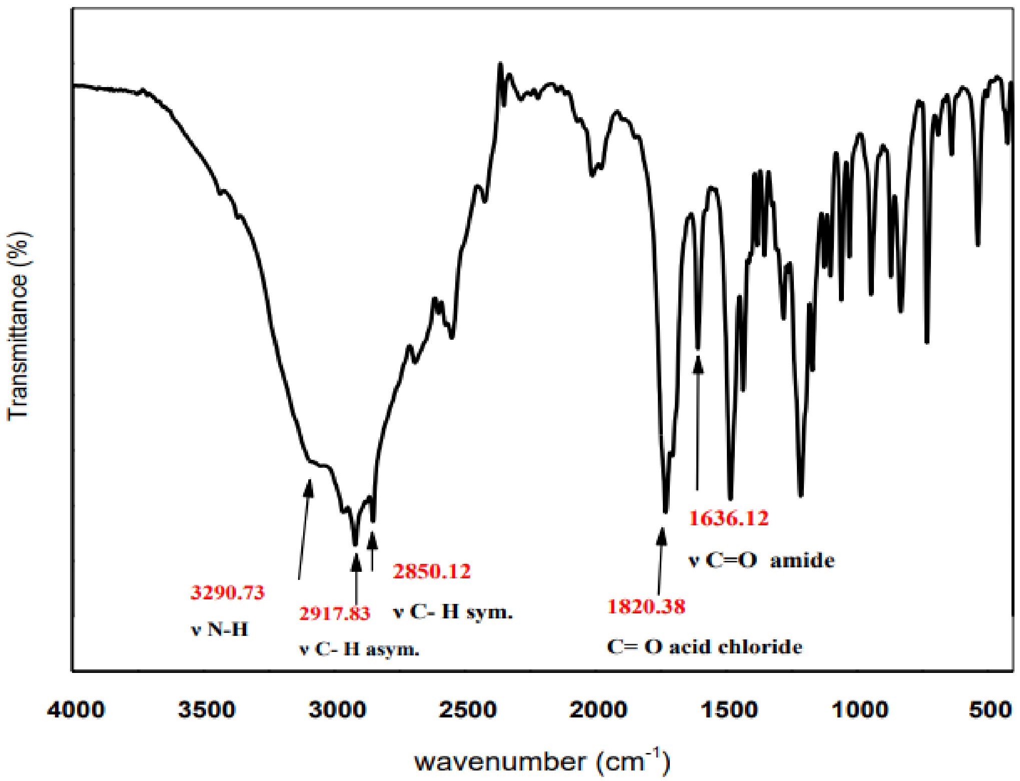

Figure S3. FT-IR Spectra for *N*- tetradecanoyl valine chloride

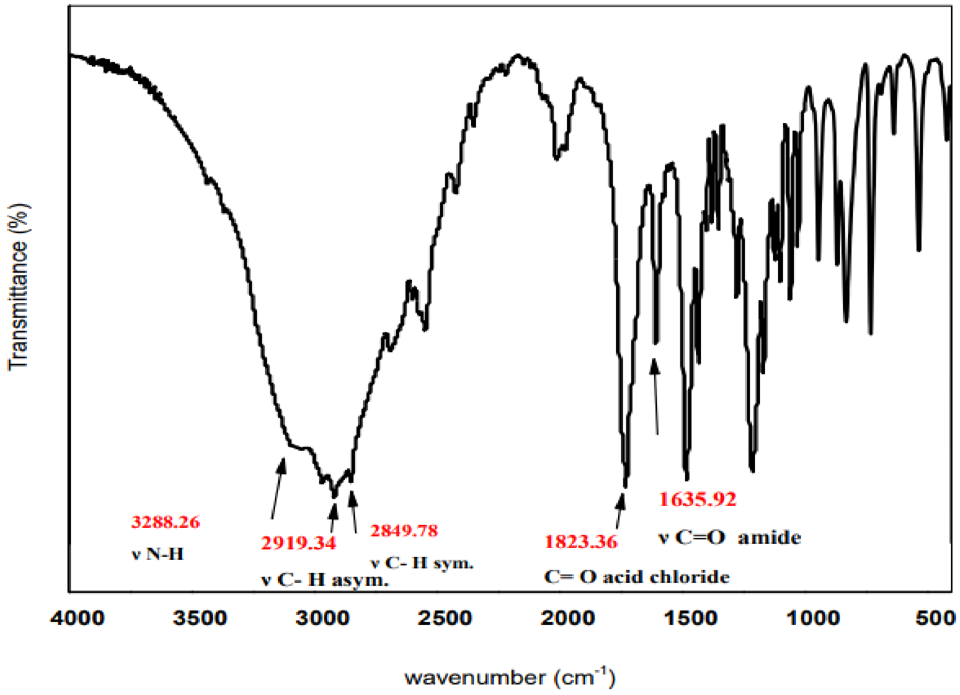

Figure S4. FT-IR Spectra for *N*- hexadecanoyl valine chloride

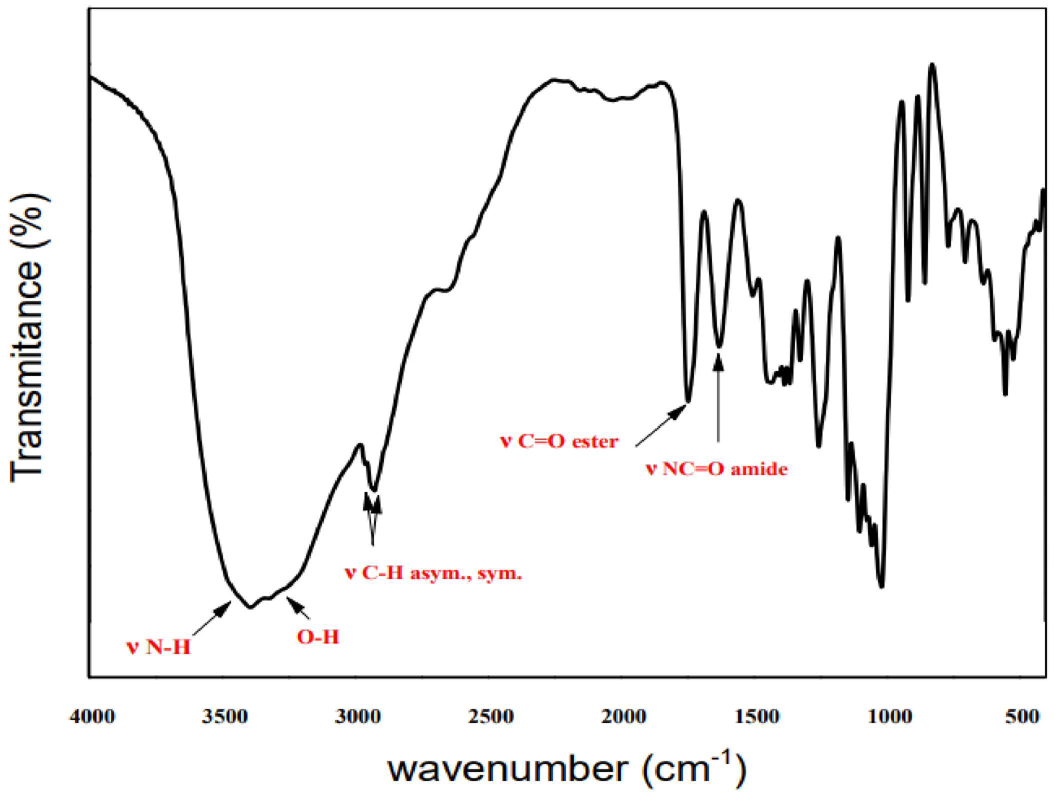

Figure S5. FT-IR Spectra for 6-*O*- (*N*-dodecanoyl glycine)-glucopyranose

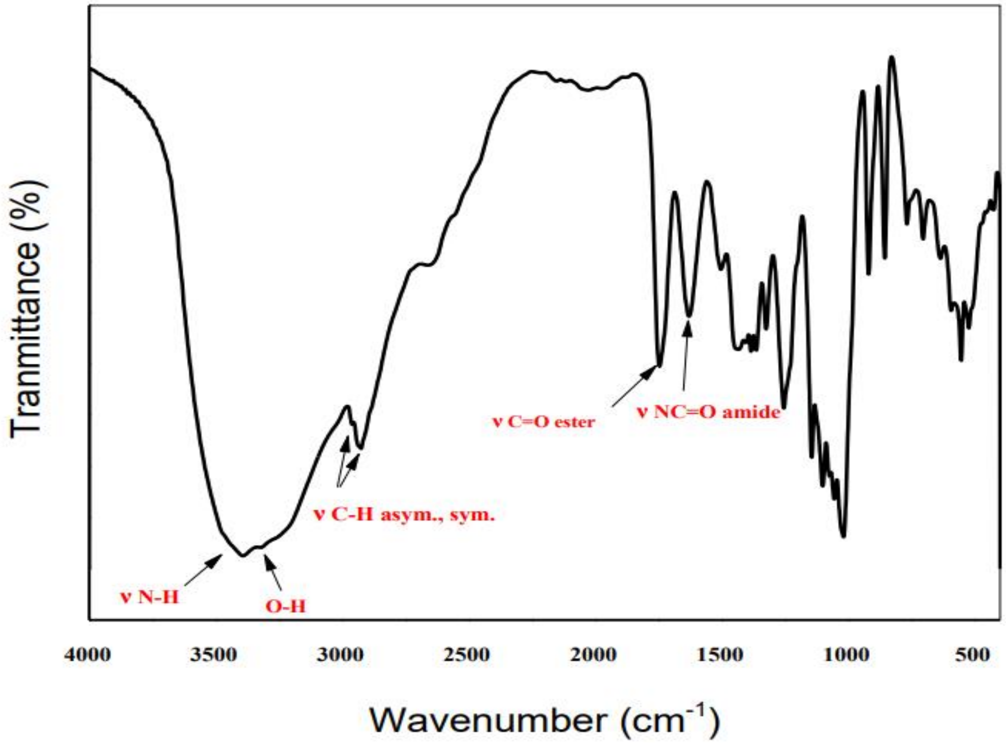

Figure S6. FT-IR Spectra for 6-*O*- (*N*-tetradecanoyl glycine)-glucopyranose

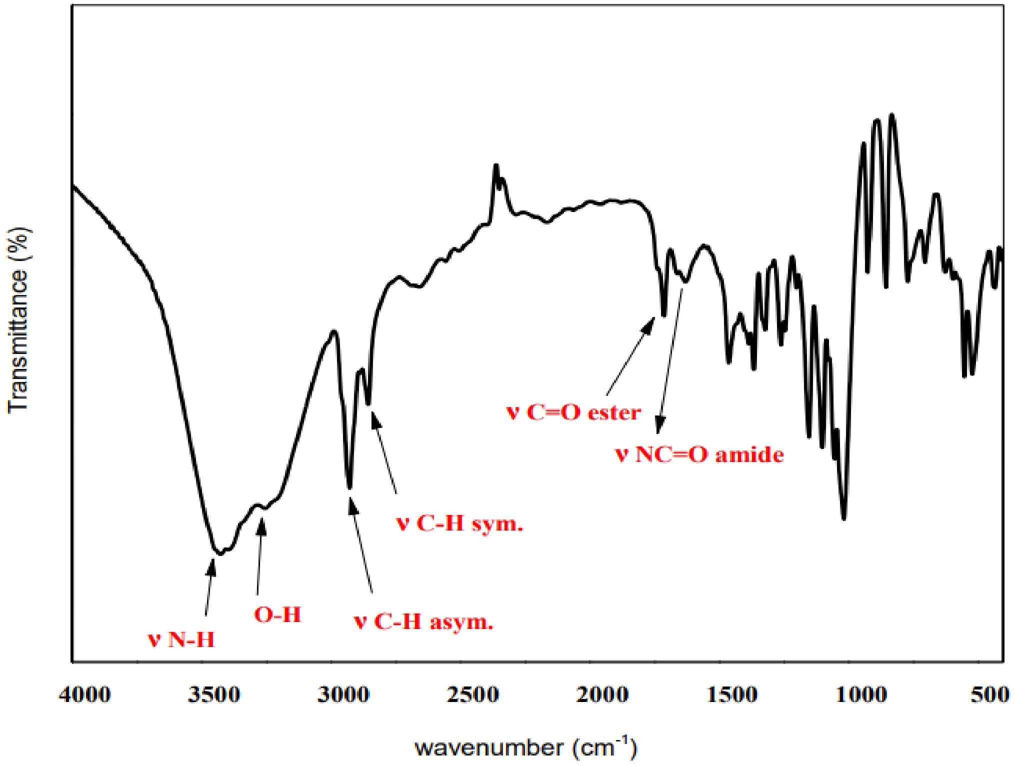

Figure S 7. FT-IR Spectra for 6-*O*-(*N*-9-octadecenoyl glycine)-glucopyranose

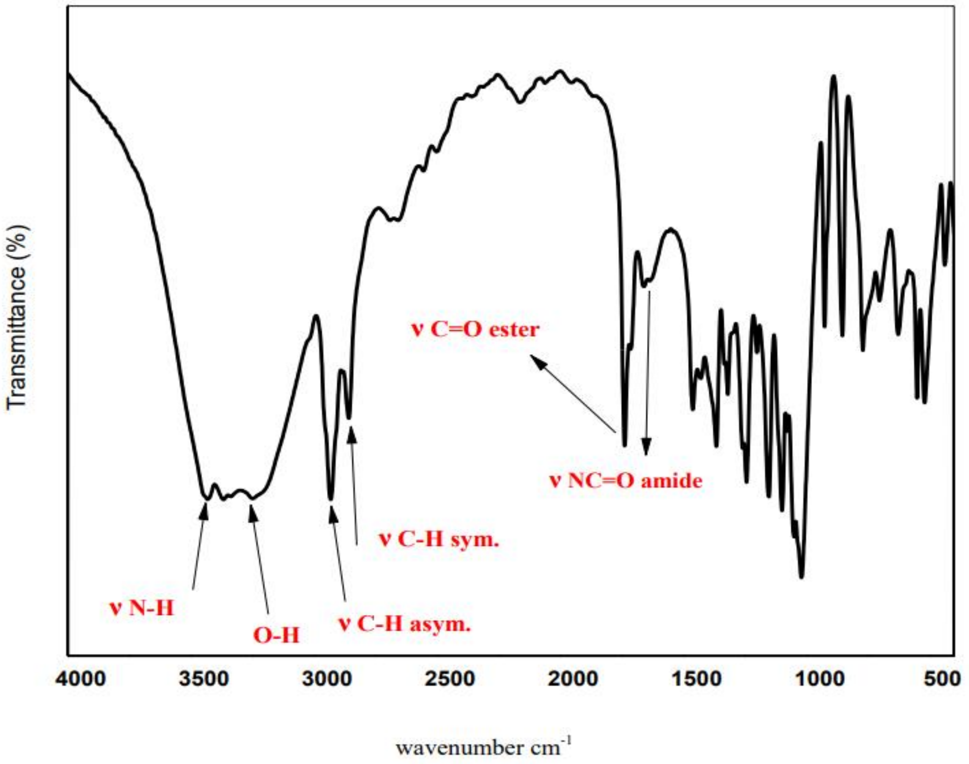

Figure S 8. FT-IR Spectra for 6-*O*-(*N*-12-hydroxy-9-octadecenoylglycine)-glucopyranose

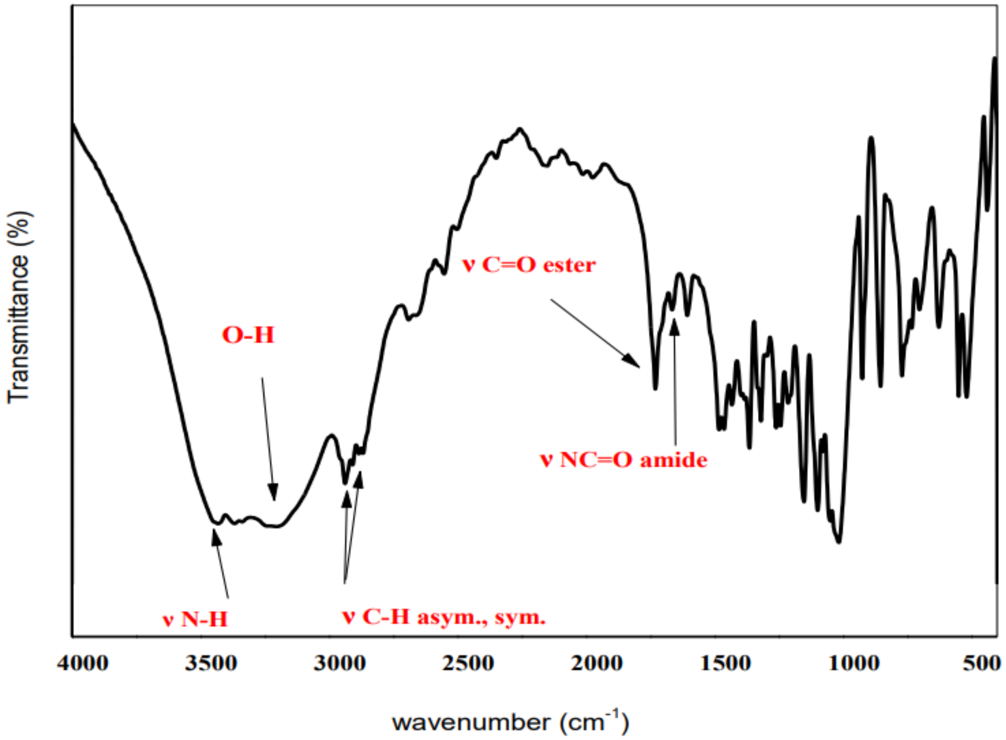

Figure S 9. FT-IR Spectra for 6-*O*- (*N*-dodecanoyl valine)-glucopyranose

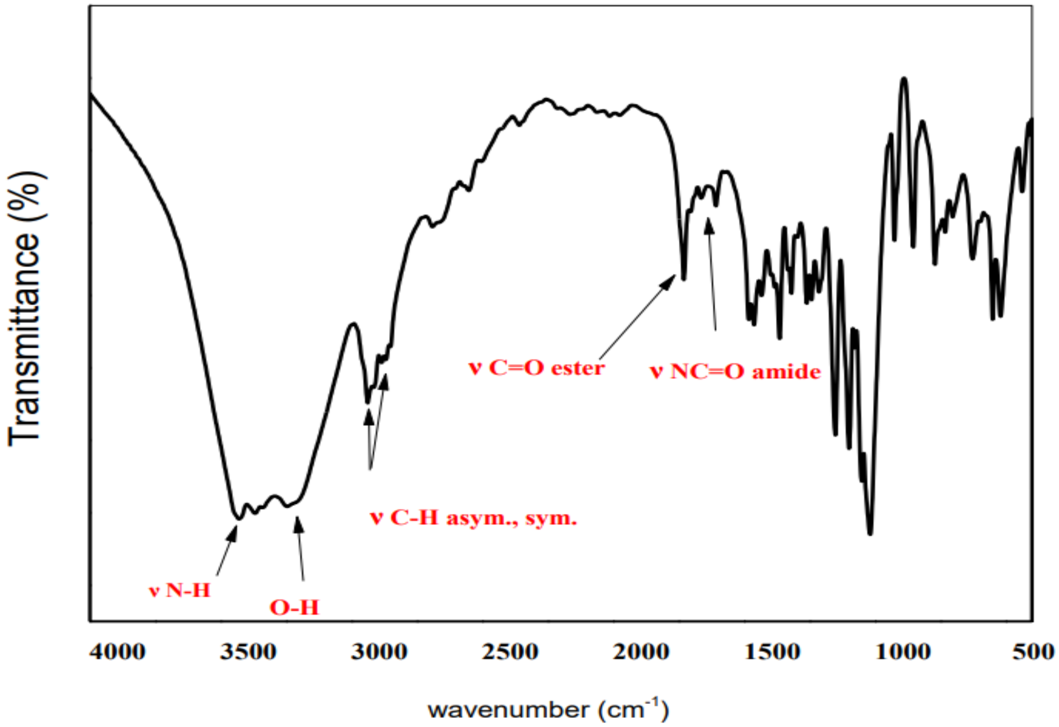

Figure S10. FT-IR Spectra for 6-*O*- (*N*-tetradecanoyl valine)-glucopyranose

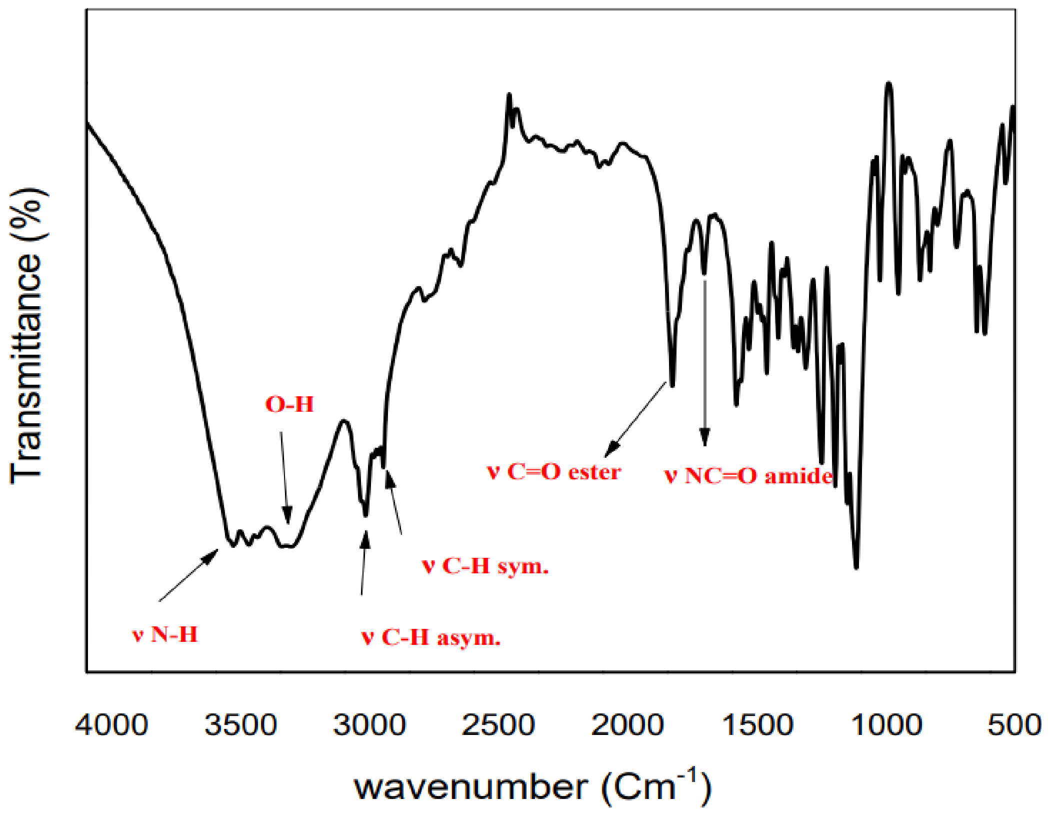

Figure S11. FT-IR Spectra for 6-*O*- (*N*-hexadecanoyl valine)-glucopyranose

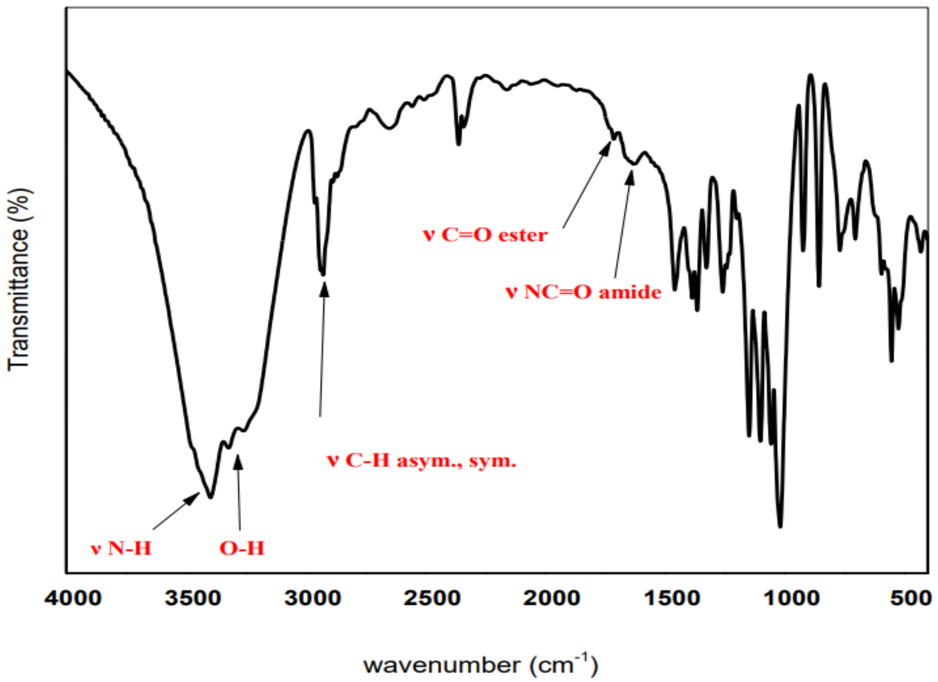

Figure S12. FT-IR Spectra for 6-*O*-(*N*-9-octadecenoyl valine)-glucopyranose

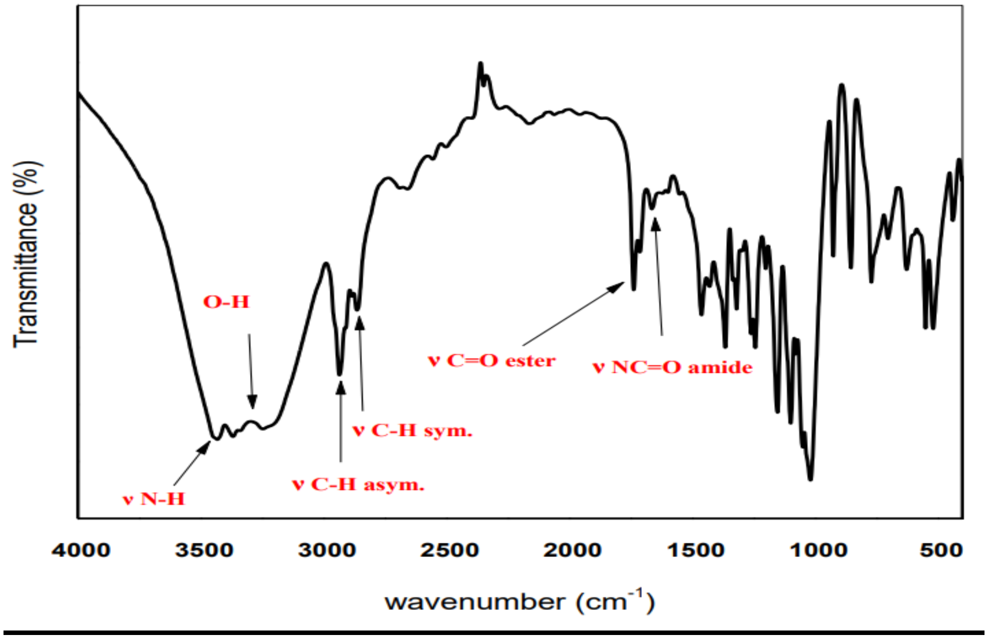

Figure S13. FT-IR Spectra for 6-*O*-(*N*-12-hydroxy-9-octadecenoylvaline)-glucopyranose

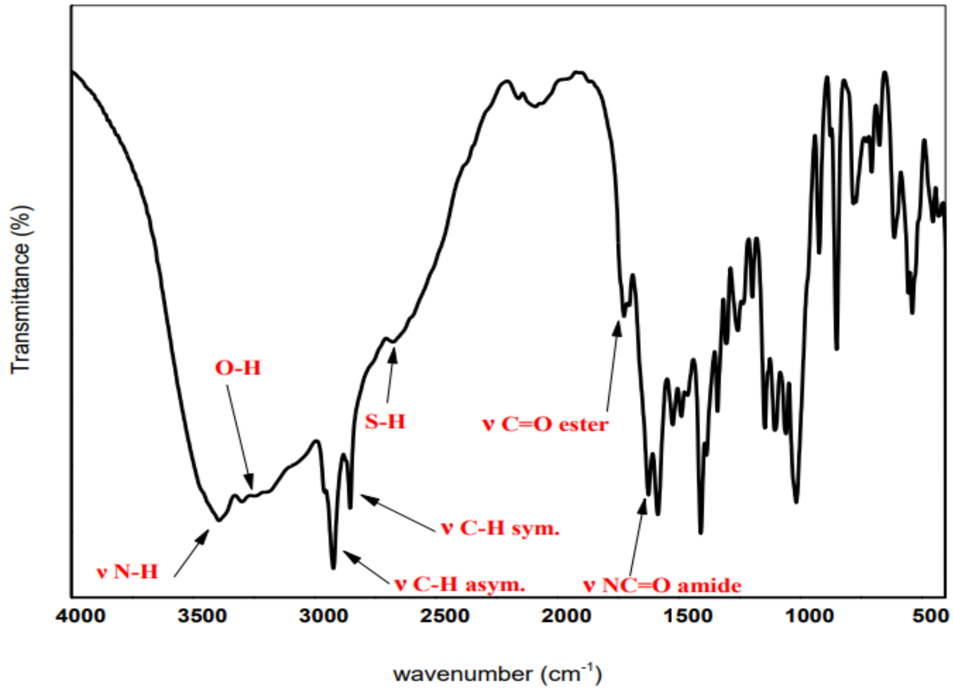

Figure S14. FT-IR Spectra for 6-*O*- (*N*-dodecanoyl cysteine)-glucopyranose

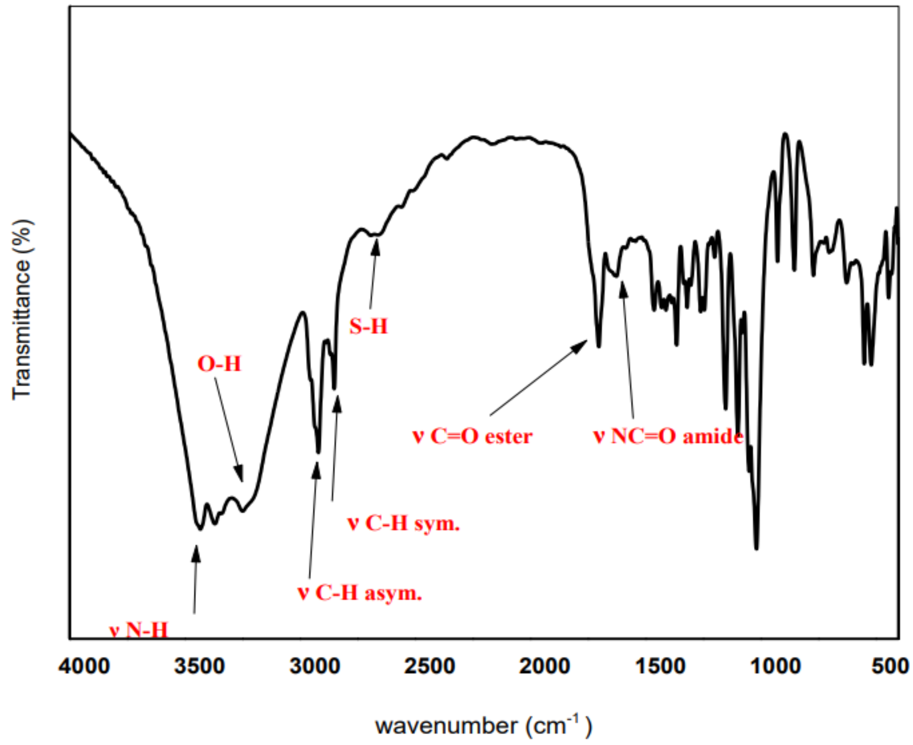

Figure S15. FT-IR Spectra for 6-*O*- (*N*-tetradecanoyl cysteine)-glucopyranose

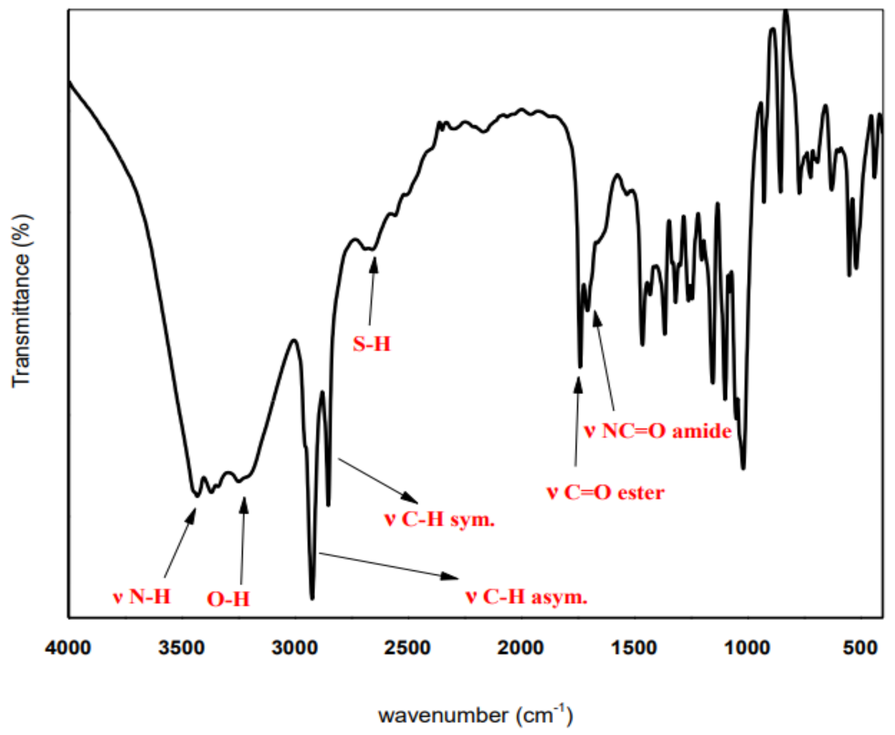

Figure S16. FT-IR Spectra for 6-*O*- (*N*-hexadecanoyl cysteine)-glucopyranose

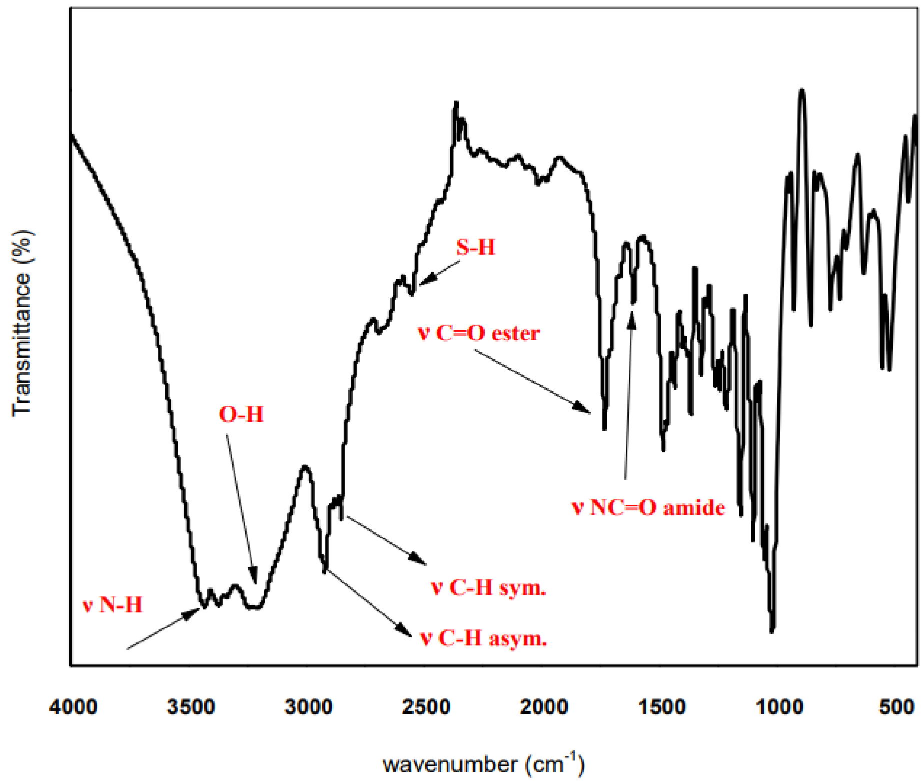

Figure S17. FT-IR Spectra for 6-*O*- (*N*-9-octadecenoyl cysteine)-glucopyranose

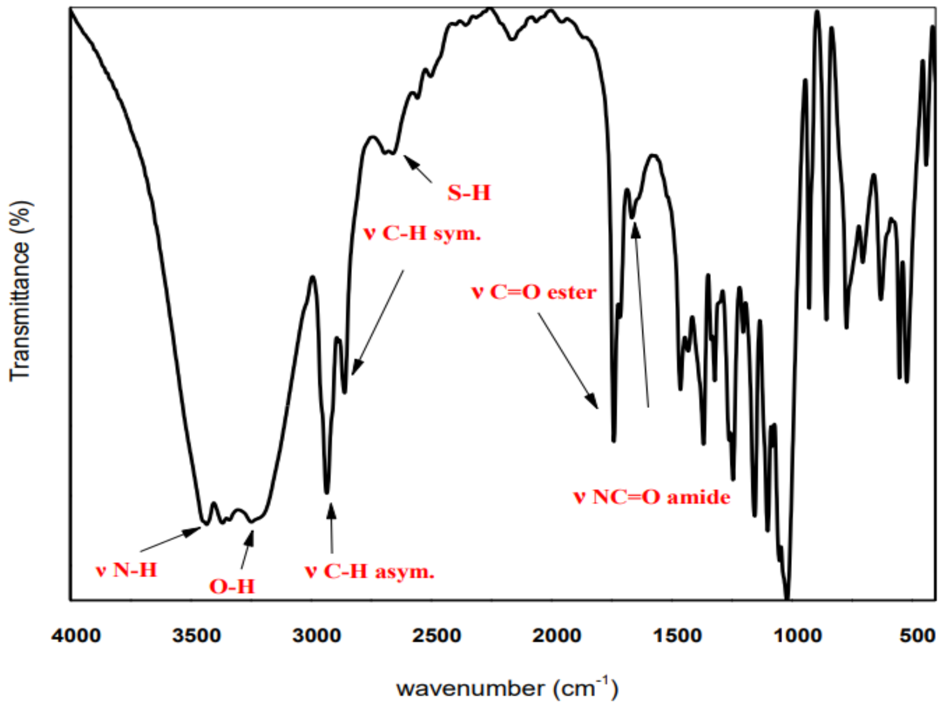

Figure S18. FT-IR Spectra for 6 -*O*-(*N*-12-hydroxy-9-octadecenoyl cysteine)-glucopyranose

Proton Nuclear Magnetic Resonance (^{1} H NMR)

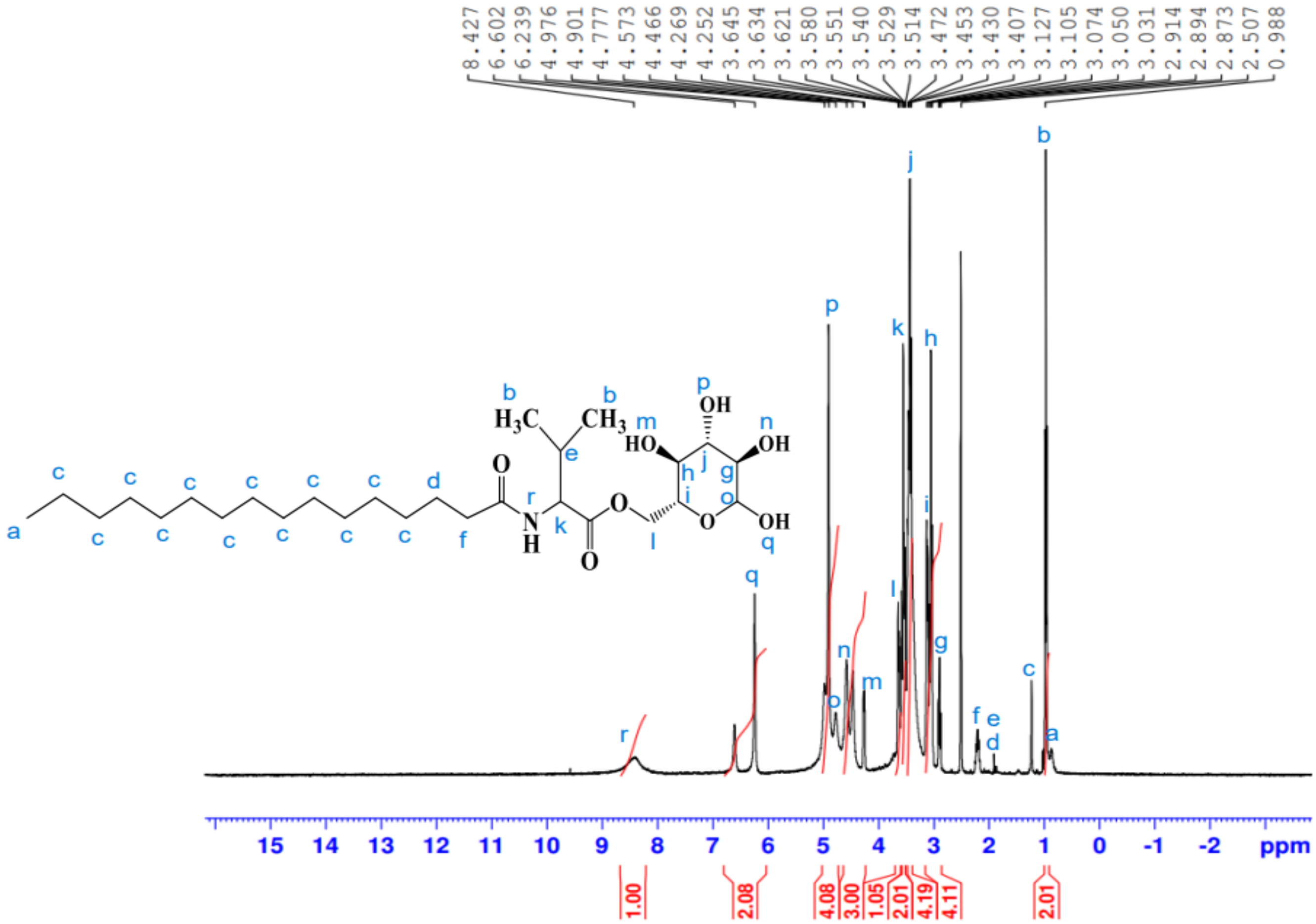

Figure S19. ^{1}H-NMR Spectra for 6-*O*- (*N*-hexadecanoyl valine)-glucopyranose

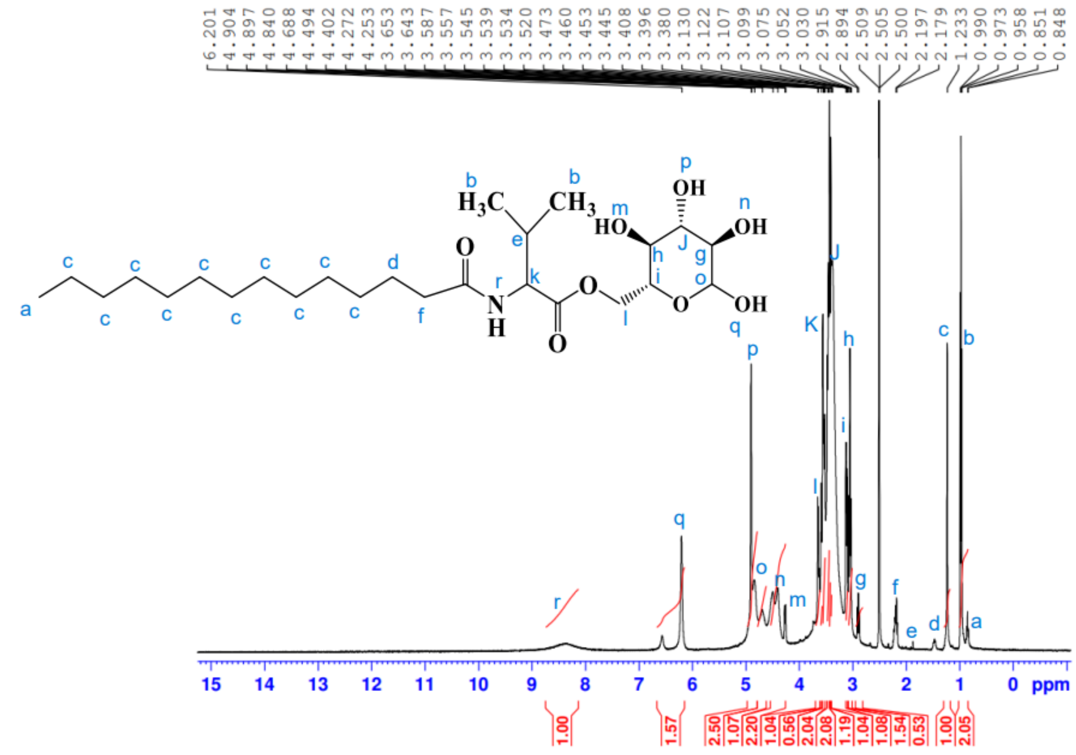

Figure S20. ^{1}H-NMR Spectra for 6-*O*- (*N*-tetradecanoyl valine)-glucopyranose

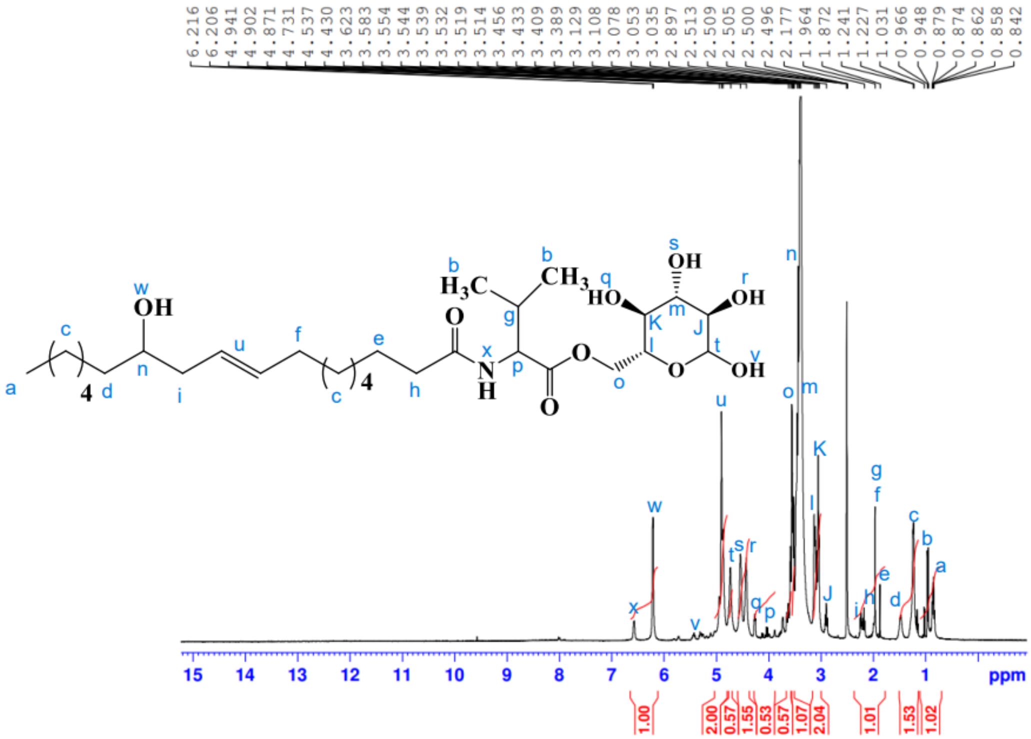

Figure S 21. ^{1}H-NMR Spectra for 6-*O*-(*N*-12-hydroxy-9-octadecenoylvaline)-glucopyranose

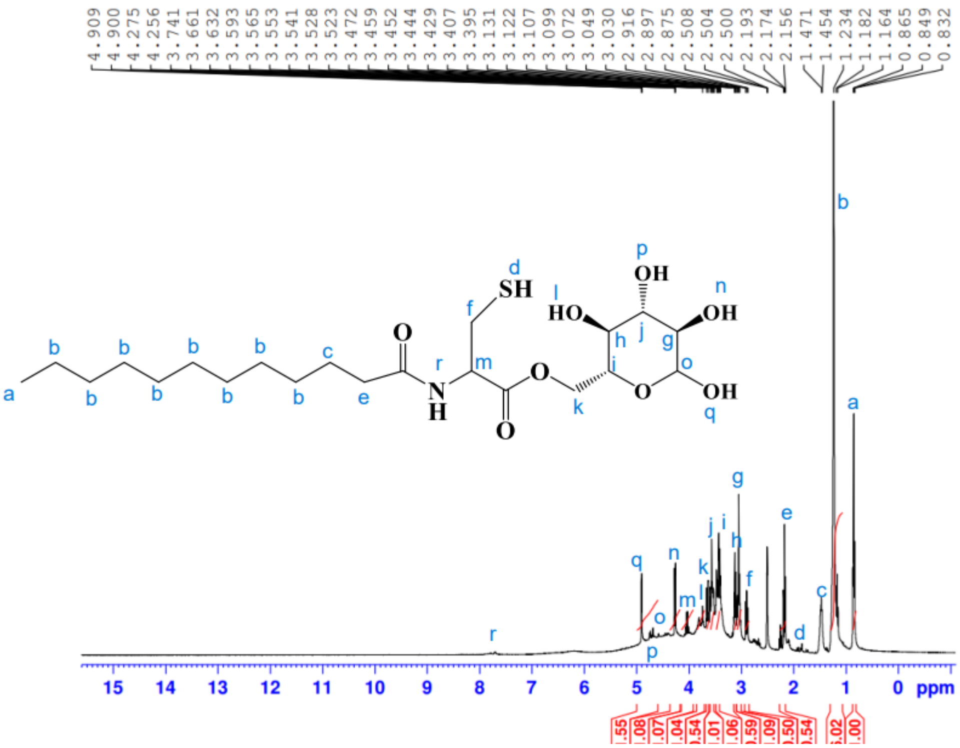

Figure S22. ^{1}H-NMR Spectra for 6-*O*- (*N*-dodecanoyl cysteine)-glucopyranose

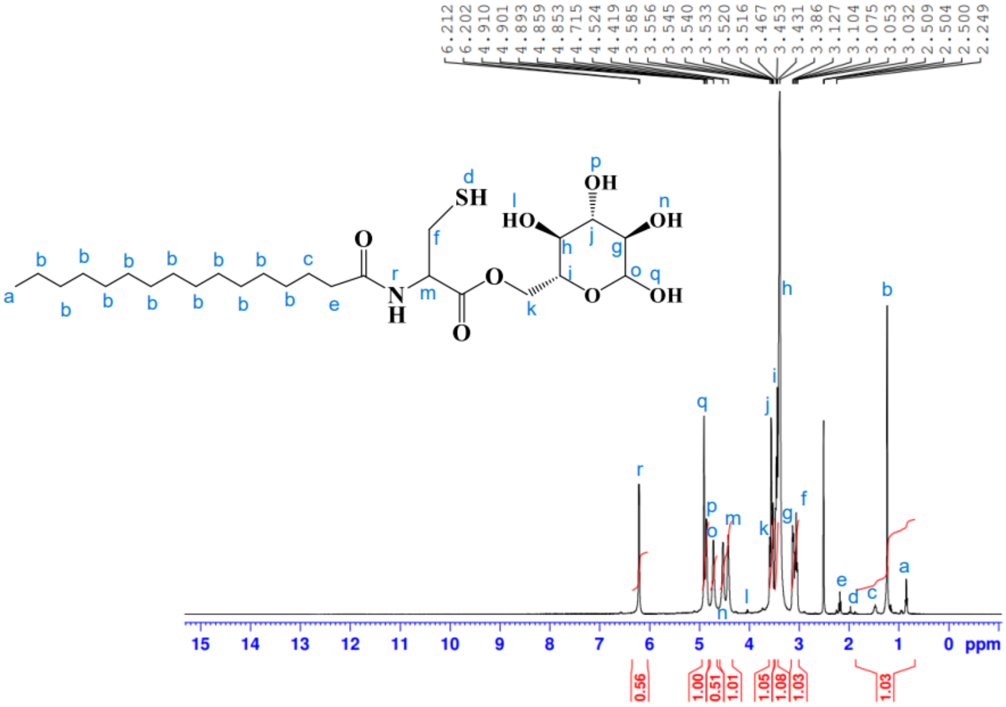

Figure S23. ^{1}H-NMR Spectra for 6-*O*- (*N*-hexadecanoyl cysteine)-glucopyranose

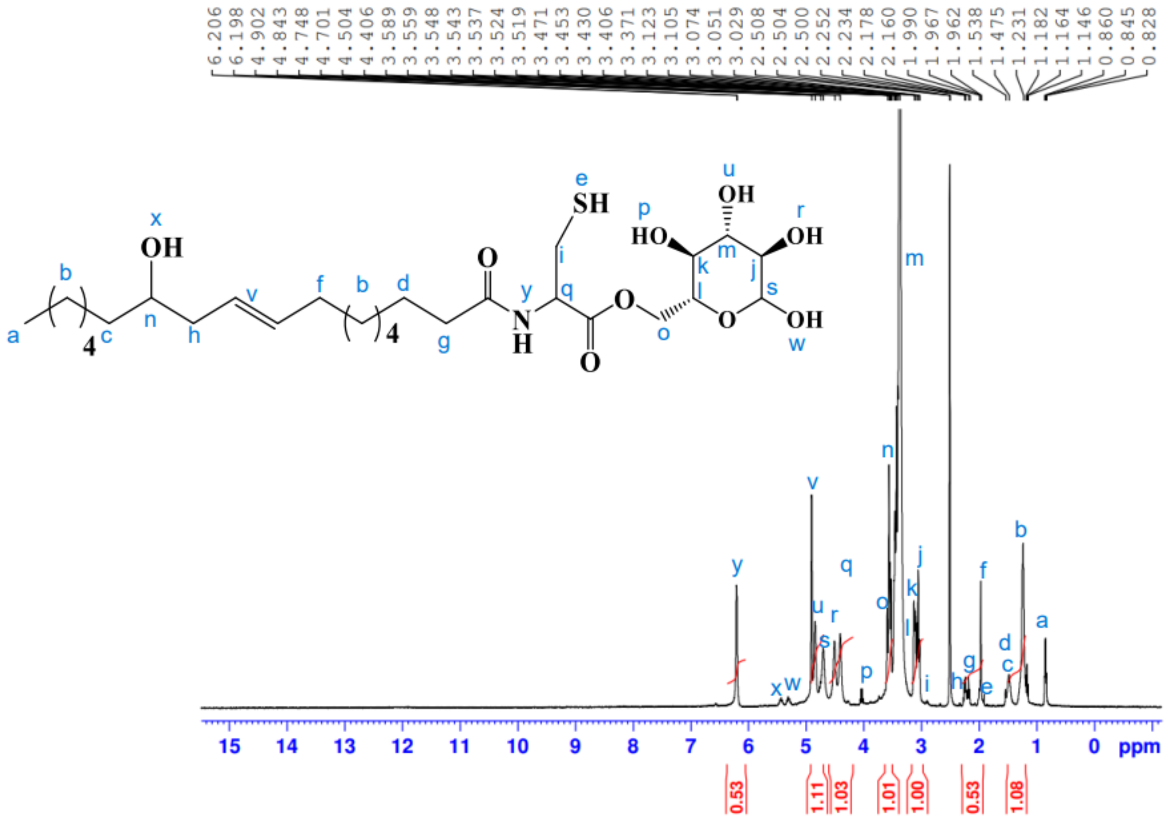

Figure S24. ^{1}H-NMR Spectra for 6-*O*-(*N*-12-hydroxy-9-octadecenoyl cysteine)-glucopyranose

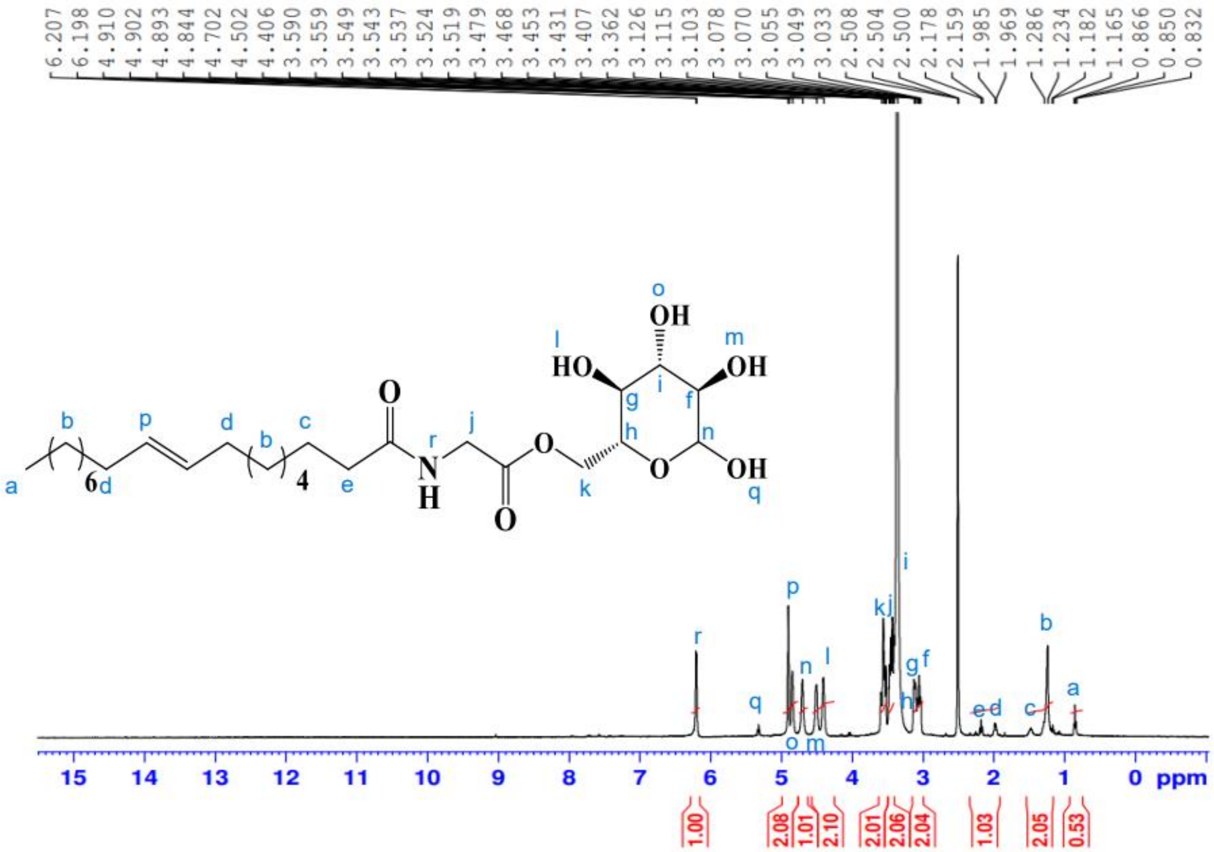

Figure S25. ^{1}H-NMR Spectra for 6-*O*-(*N*-9-octadecenoyl glycine)-glucopyranose

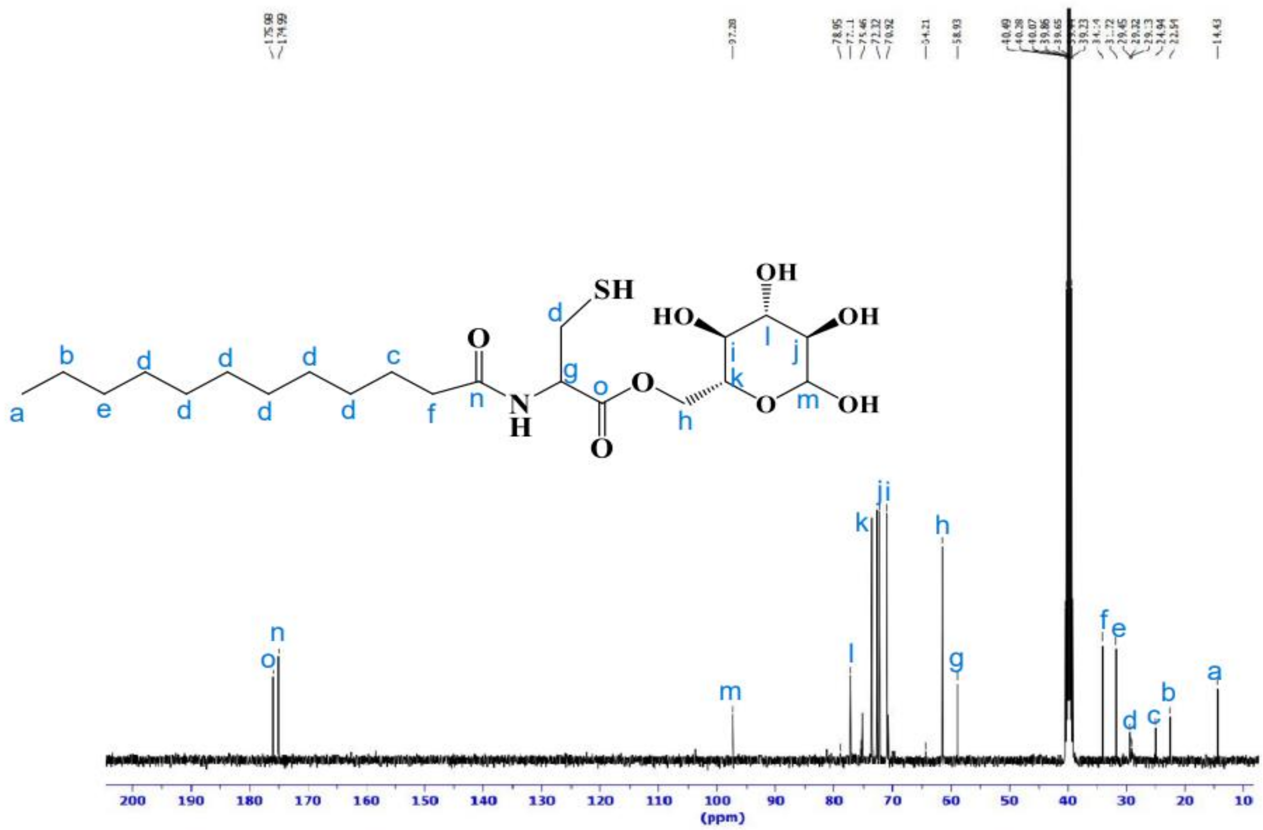

Figure S 26. ^{13}C-NMR spectrum of 6-*O*- (*N*-dodecanoyl cysteine)-glucopyranose
